# PLATERO: A calibration protocol for plate reader green fluorescence measurements

**DOI:** 10.3389/fbioe.2023.1104445

**Published:** 2023-01-20

**Authors:** Alba González-Cebrián, Joan Borràs-Ferrís, Yadira Boada, Alejandro Vignoni, Alberto Ferrer, Jesús Picó

**Affiliations:** ^1^ Multivariate Statistical Engineering Group, Department of Applied Statistics and O.R. and Quality, Universitat Politècnica de València, València, Spain; ^2^ Synthetic Biology and Biosystems Control Lab, Instituto de Automática e Informática Industrial, Universitat Politècnica de València, València, Spain

**Keywords:** plate reader, fluorescence measurements, units conversion model, measurement data standardization, measurement system analysis

## Abstract

One of the most common sources of information in Synthetic Biology is the data coming from plate reader fluorescence measurements. These experiments provide a measure of the light emitted by a certain fluorescent molecule, such as the Green Fluorescent Protein (GFP). However, these measurements are generally expressed in arbitrary units and are affected by the measurement device gain. This limits the range of measurements in a single experiment and hampers the comparison of results among experiments. In this work, we describe PLATERO, a calibration protocol to express fluorescence measures in concentration units of a reference fluorophore. The protocol removes the gain effect of the measurement device on the acquired data. In addition, the fluorescence intensity values are transformed into units of concentration using a Fluorescein calibration model. Both steps are expressed in a single mathematical expression that returns normalized, gain-independent, and comparable data, even if the acquisition was done at different device gain levels. Most important, the PLATERO embeds a Linearity and Bias Analysis that provides an assessment of the uncertainty of the model estimations, and a Reproducibility and Repeatability analysis that evaluates the sources of variability originating from the measurements and the equipment. All the functions used to build the model, exploit it with new data, and perform the uncertainty and variability assessment are available in an open access repository.

## 1 Introduction

The transition of Synthetic Biology from a trial-and-error process to an engineering discipline embracing more formal methods requires standards. These facilitate the Design-Build-Test-Learn (DBTL) lifecycle by enabling the integration of inherently different tools and methods into coherent workflows (Carbonell et al., 2018). The DBTL cycle requires a complete description of the components in a biological system, data to describe the system function and interconnections, and computational models to predict the impact of environmental parameters on the system behavior. In this context, data standards describing genetic constructs and their mathematical models foster the sharing of information, key to overcoming characterization and reproducibility issues across laboratories (Aldulijan et al., 2022).

Reproducibility can be ensured by establishing an unbroken chain of calibrations to specified reference standards (Gaigalas et al., 2005) and quality control of the reference materials used for calibration. Using calibrated absolute standard units and protocols allows for bringing measurements and estimations results from different sources or measurement device settings into a common domain so that they can be integrated and compared faithfully (Boada et al., 2019).

The expression of fluorescent reporters is commonly used for quantifying gene expression levels. Fluorescent dyes are also used for quantifying a wide range of other biological properties. Two main classes of devices are used for measuring fluorescence: flow cytometers and plate readers (Lakowicz, 1999). A measure of the light emitted by a certain fluorescent molecule, e.g. the Green Fluorescent Protein (GFP), is used to estimate the amount of GFP molecules expressed by the cell. Thus, by linking the expression of a gene of interest to that of GFP, the measurement of fluorescence can be used as an indirect measure of the expression level of the first one (Beal et al., 2021).

Two main problems affect the proper characterization of gene expression using measurements of fluorescence (Boada et al., 2019). On the one hand, the values obtained are affected by the measurement device setup. In particular, the gain of the device is set so that measurements do not saturate. As a consequence, for a series of related experiments spanning a wide range of fluorescence intensities, it is common that different device gains must be used. This makes a comparison of results a difficult task, as the relationship between the actual fluorescence and the gain-affected measurement may be non-linear. On the other hand, fluorescence measurements are usually expressed in arbitrary units.

Some studies have been trying to normalize fluorescence measurements with a biological sample cultured in parallel with the experimental samples (Kelly et al., 2009). However, such normalization may produce less precise measurements than normalization using an independent calibrant, due to the ill-defined potential variability of the biological samples used for normalization (Beal et al., 2018). The most similar attempt to deal with the same issues as PLATERO, is the FlopR software by Fedorec et al. (2020), which provides solutions for the standardization of plate reader and flow cytometry data. Non-etheless, there are three main aspects worth mentioning about this approach. First, the FlopR approach gives several models as options for the data correction, which might invalidate the comparison of different plate readers *via* their correction coefficients if these have been fitted assuming different correction models. Secondly, the validation of the results relies on a qualitative comparison between the corrected fluorescence values of well-known strong promoters and the values reported in the literature, without any concluding statistical assessment. Thirdly, FlopR does not account for any measurement system analysis quantifying the uncertainty for the model’s predictions or the plate reader’s reproducibility and repeatability. On a related note on the issue of comparing and assessing measurements from different plate readers, it is worth mentioning as well the software FlowCal from Castillo-Hair et al. (2016). Despite working with flow cytometry data instead of plate readers, authors considered in this work the data transfer between two different machines. However, this is done by considering a linear relationship between the corrected data from each machine, and it lacks any measurement system’s analysis.

For all these reasons, it seems reasonable to propose an approach to convert fluorescence data from plate readers using a unified mathematical framework for the assumptions of the normalization steps, and with a set of statistical tools providing a validation of the mathematical assumptions, a quantification of the uncertainty in the model predictions, and an assessment on the plate reader’s measuring quality that also enables the comparison between different machines.

**FIGURE 1:**
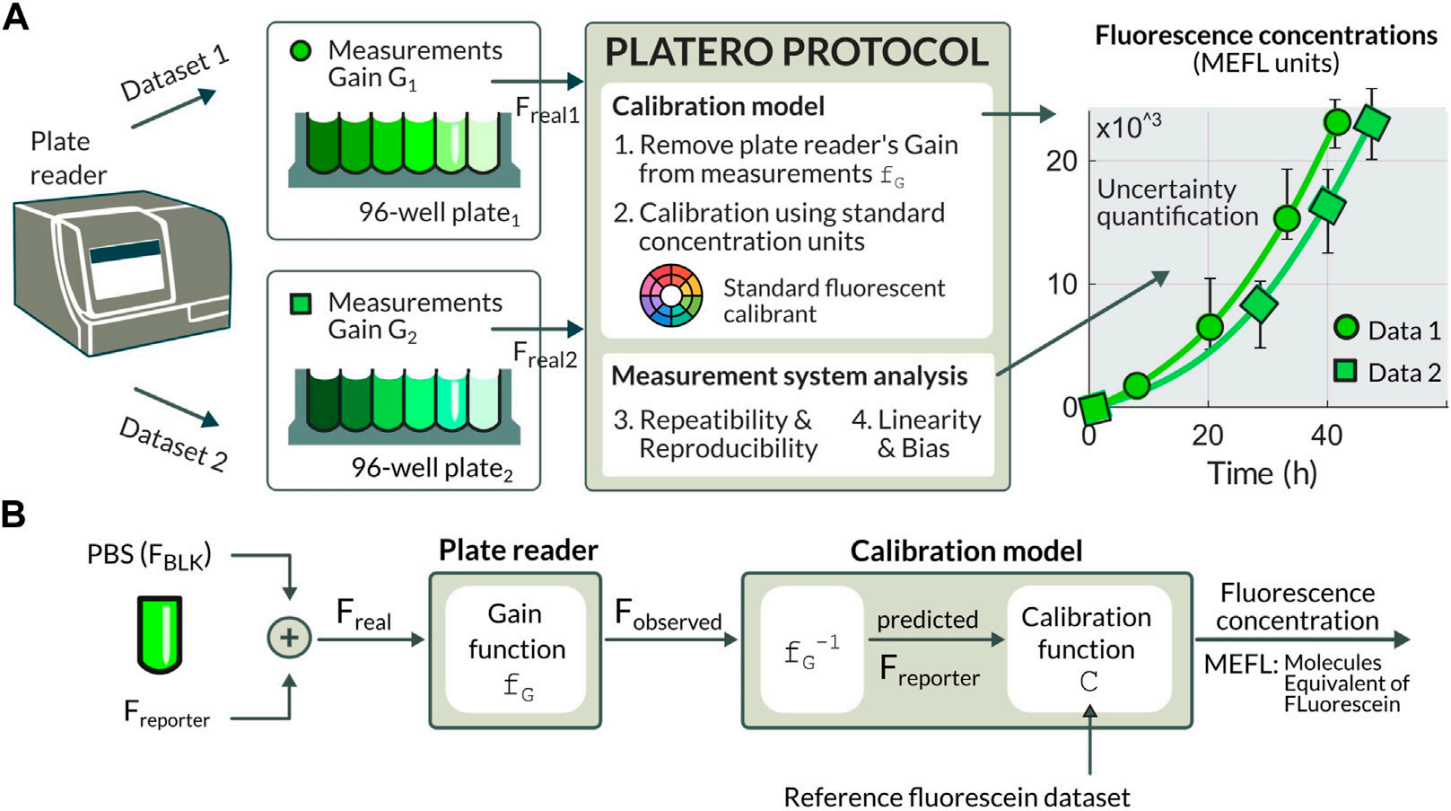
**(A)** The PLATERO calibration model brings the experimental measurements into a common gain-independent domain using standard concentration MELF units. The calibration protocol embeds a Measurement System Analysis providing estimation for the uncertainty that can be expected on the predicted concentration value, and an assessment of the plate reader being used and the sources of uncertainty. **(B)** Diagram of the procedure to retrieve concentration values from observed fluorescence (*F*_*observed*_). The *F*_*observed*_ values are a function (*f*_*G*_) of the medium fluorescence (*F*_*BLK*_), the fluorescence of the reporter (*F*_*reporter*_), and the gain (*G*) at which fluorescence values are measured. Once the gain and background effects are removed, the *F*_*reporter*_ values are retrieved. The units conversion function (*f*_*UC*_) transforms these corrected fluorescence values into standard concentration units.

The model used by PLATERO transforms from fluorescence measurements, which are relative to the plate reader setting and expressed in arbitrary units, to units of calibrant concentration, which are absolute, comparable, and independent of the measurement device setup. As for the measurement device setup, we propose a correction of the fluorescence readings by using a gain-effect model. To address the problem of the arbitrariness of units, we use already established protocols (Beal et al., 2018; Boada et al., 2019; Beal et al., 2021) using fluorecein as calibrant that can be used to produce precise estimates of molecules equivalent of fluorescein (MEFL), and fluorescein concentration from fluorescence measurements. The resulting units calibration model enables users of fluorescence plate readers to bring experimental measurements into a common gain-independent domain. This allows for a comparison of results obtained from different plate readers possibly located at different laboratories (Figure 1A). However, not only PLATERO can be used for the calibration of green fluorescent proteins such as GFP by using fluorescein as a calibrant, but also it is extensible to any kind of fluorescence calibration by using the appropriate calibrant. For instance, with Cascade Blue it is possible to calibrate blue fluorescent proteins such as mTagBFP, and using Sulphorhodamine 101 red fluorescent proteins can be calibrated as well (Beal et al., 2022).

A key aspect of any measurement device and its associated measurement protocol is the analysis of uncertainty of the calibration, and the analysis of variability and its sources associated to the protocol operations and the measurement device. PLATERO’s calibration protocol embeds a Measurement System Analysis (MSA) that provides both an estimation of the uncertainty that we can expect on the predicted concentration value, and an assessment of the plate reader being used and the sources of uncertainty.

PLATERO has been implemented as a Matlab toolbox, and it is freely available at https://github.com/sb2cl/PLATERO.

The remaining paper is organized as follows. In Section 2 we describe the materials used to carry out the protocol, and give a precise list of instructions. In Section 2.3 we explain the methods underlying the calibration model and the associated analysis of uncertainty. In Section 3 we describe the results that can be obtained using the protocol and how they can be assessed using the embedded Measurement System Analysis. Finally, a brief discussion is given in Section 4.

## 2 Materials and methods

### 2.1 Experimental procedure

The PLATERO protocol requires preparing serial dilutions of a reference calibrant solution to perform the fluorescence calibration. This reference solution can be prepared from the calibrant power by weighing and dissolving in a known volume. The concentration of this reference solution can be further confirmed by measuring its absorbance at maximum absorbance wavelength and calculating concentration using an extinction coefficient of the calibrant, the appropriate pathlength from your spectrophotometer (normally *ℓ* = 1 cm), and the law of Beer-Lambert as follows:

$$C=\frac{Abs_{mw}}{\varepsilon \cdot \ell }$$ (1)

The selection of the appropriate calibrant for the desired fluroescent reporter protein is not within the scope of this work, but the reader is advised to check the literature on the topic such as some previous work of some of the authors in Beal et al. (2022). The selection of the calibrant together with the fluorescent reporter properties will define the excitation and emission wavelengths to be used in the plate reader setup, which must be consistent and remain unchanged in both calibration and measurement. In addition, when obtaining fluorescent measurements from plate readers, it is normal to acquire the fluorescent signal by measuring only at the wavelength of maximum emission, which is a good representative of the entire fluorescent signal (the area under the emission spectra) (Lakowicz, 2006). Not only this practice is common but also is necessary since the time available to perform a measurement with a plate reader, possibly considering a time series experiment, is very short. Moreover, measuring the area under the emission spectra is only possible with a spectrofluorometer (not the time of equipment of interest for this work) or with a plate reader which has a monochromator (Lakowicz, 1999).

Once the calibrant is selected according to the characteristics of the fluorescent reporter of interest, a key aspect to be taken into account is its photostability. For the spectral range we are interested in this work, i.e. green fluorescent proteins, fluorescein has been extensible studied and used (Zhang et al., 2014). For instance, in (Song et al., 1995), a solution of 0.01uM of fluorescein was exposed to a 100 W lamp with 450–490 nm filter for 10 min presented a reduction in the fluorescence due to photobleaching of 6.6%. While, most plate readers have Xenon flash light sources, with at most 20 W of power and an exposition sample time of 20 ms per read, making any photobleaching effect to be very small and comparable with the other sources of variability in the measurement.

As a test and demonstration on how to use PLATERO we will work with a fluorescein sodium salt solution (Sigma-Aldrich *#*46970) (Beal et al., 2021) to be used as calibrant for green fluorescent proteins such as GFP. The reference solution is prepared from the fluorescein sodium salt power by weighing and dissolving in a known volume. The concentration of this reference solution was further confirmed by measuring its absorbance at 492 nm and calculating concentration using an extinction coefficient of 68.029 mM^−1^ cm^−1^, the pathlength the spectrophotometer (*ℓ* = 1 cm).

Starting from 1 ml of the 10 *μ*M reference solution of fluorescein in Phosphate Saline Buffer (PBS), the experimental protocol described in (Vignoni and Boada, 2022), modified from (Boada et al., 2019; Beal et al., 2022)), has to be carried out to get serial dilutions.

In short, by following the protocol, one obtains a serial dilution of fluorescein with five increasing concentrations plus an only PBS solution for blanks (*F*_*BLK*_). In our case, we used the concentrations 0.0391, 0.0781, 0.1562, 0.3125, and 0.625 *μ*M. Samples of this serial dilution have to be randomly transferred into a 96-well black/clear flat bottom microplate with 16 replicates per concentration. An example of such a random distribution can be seen in the Supplementary Material—Experimental Protocol. Therefore, the 96-well plate ends up containing 16 technical replicates per each of the five fluorescein concentrations, and 16 technical replicates of the blank *F*_*BLK*_. Fluorescence measurements of the 96-well plate using a plate reader have to be repeated 8 times. The plate reader has to be configured so as to cover a wide range of the spectrum of gains of the plate reader. In our case, to show the capabilities and benefits of using PLATERO, we used the Agilent BioTeK Cytation 3 Cell Imaging Multi-Mode Reader, and configured it using an excitation/emission wavelength of 488/530 nm. In addition, arranged it at four different detection gain levels, *G* = 50, 60, 70, 80.

### 2.2 Description of the database

The concentrations and repetitions obtained in the experimental protocol above, were arranged in a database using the following Design Of Experiments (DOE): for a crossed design with two factors involved (Well and Gain), the measurement instrument measured *I* wells, at *J* gains for *K* repetitions or replicas. This way, one set of *I* × *J* × *K* measurements was obtained for each one of the *L* concentration levels. Particularly, our database had 2048 observations. That is, *L* = 4 concentrations (3 fluorescein + 1 empty) × 16(*I*) wells × 4(*J*) gains × 8(*R*) measurement repetitions. Thus, each observation combined a concentration level, a well, the gain used for its acquisition, and the number of replicas. The resulting data and test software is publicly available as a Zenodo repository (González-Cebrían et al., 2022).

### 2.3 Calibration model

In this section, we first describe the calibration model used by PLATERO, which enables the conversion from fluorescence arbitrary units to concentration expressed as the equivalent concentration of fluorescein. Next, we show how the estimation of the uncertainty was included as the final step of the calibration model fitting so as to validate the conversion model. We show how the Linearity and Bias analysis (L&BA) is applied to obtain the uncertainty of the estimated concentrations provided by the protocol model. We used a test based on the confidence interval built around the estimation for the true concentration of reporter within a well. If this confidence interval contains the true concentration value, we will consider the estimation valid. Finally, we describe how to apply the Reproducibility and Repeatability analysis (R&RA) to assess the different sources of the observed variability in the estimations.

A detailed list of the steps required to apply the full calibration protocol is included in Supplementary Appendix S4. In addition, the Matlab functions performing each step can be found within the PLATERO toolbox available from the GitHub repository https://github.com/sb2cl/PLATERO. Throughout the following subsections, some references to the corresponding functions of the toolbox are provided.

Figure 1B depicts the different factors involved in the measurement of fluorescence provided by a plate reader. To obtain the calibration model used by PLATERO we have to 1) compensate for the background fluorescence and correct the effect of plate reader gain on the fluorescence observations, and 2) convert the arbitrary units of fluorescence of these observations to standard fluorescein concentration units.

#### 2.3.1 Device gain and background fluorescence

The plate reader gain is one of the key parameters to set up before measuring the fluorescence of a reporter. If the gain is too low, the lower limit of the measured fluorescence range will not be correctly detected by the instrument. Conversely, if the gain is too high, the upper limit of the fluorescence range will saturate, so it cannot be measured. The relationship between the actual fluorescence in a sample (*F*_*real*_), and the fluorescence measured by the plate reader (*F*_*observed*_) is a non-linear function of the gain, as depicted in Figure 1.

To obtain the relationship between *F*_*real*_ and *F*_*observed*_, we carried out an iterative model search looking at the experimental relation between fluorescence *F*_*real*_ and the gain *G* (see Supplementary Appendix S1). From this, we inferred an exponential relationship between *F*_*real*_ and *F*_*observed*_ with a gain-dependent quadratic term in the exponent:

$$F_{\mathrm{observed}}=f_{G}\left(F_{\mathrm{real}},G\right)=F_{\mathrm{real}}\cdot e^{b_{1}\cdot G+b_{2}\cdot G^{2}}$$ (2)

where *b*_1_ y *b*_2_ are the coefficients of the linear and the quadratic terms, respectively, modeling the exponential effect of the gain on the fluorescence. This exponential gain effect on fluorescence data was also reported by Castillo-Hair et al. (2016). We assumed that the gain correction (Eq. 2) does not depend on the values of measured fluorescence. That is, its structure and the values of the coefficients depend on the measurement device but not on the range of the fluorescence.

Next, we considered a simple additive relation between the fluorescence signal in a well *F*_*real*_, the actual reporter fluorescence *F*_*reporter*_, and the inherent fluorescence background *F*_*BLK*_:

$$F_{\mathrm{real}}=F_{\mathrm{reporter}}+F_{\mathrm{BLK}}$$ (3)

**FIGURE 2:**
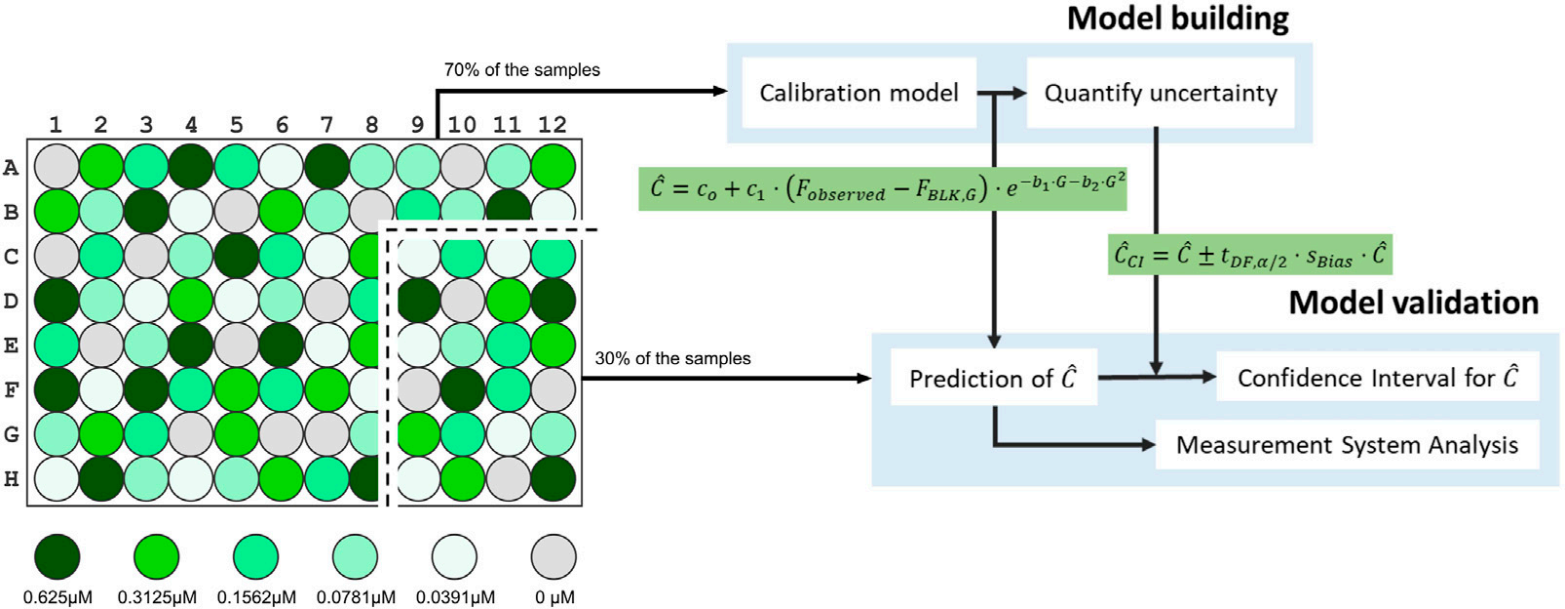
Schema representing the assessment on the proposed model done by a model building and a model validation step. Particularly, eleven out of the sixteen wells (≈70%) for each concentration level were randomly selected for the Model Building step, and the rest were used for the Model Validation.

As depicted in Figure 2, *F*_*real*_ is the input signal to the plate reader. In practice, it is not feasible to separate the *F*_*reporter*_ signal from the *F*_*BLK*_ noise for each well. The background term *F*_*BLK*_ is usually estimated by having some wells with culture medium but no fluorescent reporter, and obtaining their averaged measured fluorescence. This common estimate is then used to retrieve the *F*_*reporter*_ value for each of the wells in the plate. In our case, *F*_*BLK*_ was estimated as the median fluorescence value of wells containing only PBS buffer, acquired at the same gain (Section 2.1). The function *checkblk.m* in the PLATERO toolbox provides fast analysis of the blank wells, and also prevents including potential outliers which could distort the estimation of *F*_*BLK*_.

Replacing Eq. 2 in Eq. 3, we can obtain the true fluorescence value of the signal of interest *F*_*reporter*_:

$$F_{\mathrm{reporter}}=\left(F_{\mathrm{observed}}-F_{\mathrm{BLK,G}}\right)\cdot e^{-b_{1}\cdot G-b_{2}\cdot G^{2}}$$ (4)

where *F*_*BLK,G*_ refers to the *F*_*BLK*_ estimate at a certain gain level *G*.

The function *gaincfs.m* in the PLATERO toolbox computes the coefficients *b*_1_ and *b*_2_ in Eq. 4. Further details about the use of this function can be found in the toolbox documentation.

#### 2.3.2 Conversion of concentration units

To convert the arbitrary units of fluorescence to standard fluorescein concentration units, we assumed a linear model between the reporter fluorescence (*F*_*reporter*_) and a concentration, *C*:

$$C=f_{UC}\left(F_{\mathrm{reporter}}\right)=c_{0}+c_{1}\cdot F_{\mathrm{reporter}}$$ (5)

where *f*_*UC*_ is the units conversion function, *F*_*reporter*_ is obtained from Eq. 4, *c*_0_ is the intercept term of the linear model and *c*_1_ is the slope of the linear model. Notice one might expect a calibration curve containing the (0,0) point (no fluorescence measured at 0 nM concentration, i.e. *c*_0_ = 0). However, the coefficient *c*_0_ is important to capture offset biases introduced by the plate reader.

The estimation of the coefficients *c*_0_ and *c*_1_ in Eq. 5 is implemented in the function *cfcoeff.m* in the PLATERO toolbox. This function returns the estimated values, together with further information about the quality of the fitting.

Finally, the calibration model for the fluorescence concentration can be expressed as in Eq. 6, where the correction of the gain effect (Eq. 4) and the conversion of units to equivalent fluorescein concentrations (Eq. 5) are included.

$$C=c_{0}+c_{1}\cdot \left(F_{\mathrm{observed}}-F_{\mathrm{BLK,G}}\right)\cdot e^{-b_{1}\cdot G-b_{2}\cdot G^{2}}$$ (6)

### 2.4 Measurement system analysis

Determining the quality of the measurement system is a critical aspect to trust the readings of any measurement system. This is done by evaluating the Repeatability and Reproducibility (R&RA) and the Linearity and Bias (L&BA) analyses. On one hand, the L&BA assesses the variability of the predictions yielded by Eq. 6 along the range of concentration values, i.e., how much variability should be expected in the predictions.

On the other hand, the R&RA allows us to quantify and decompose the uncertainty as the sum resulting from the different sources of variability, i.e., where is that variability in the predictions coming from. Since the (R&R) analysis will be performed with data already expressed as the predicted concentration (Eq. 6), assessing the variability of the measurement system will include:

• variability due to lack of repeatability: “do we get the same predicted concentration value if we measure the same well several times under identical conditions?”
• variability due to lack of reproducibility: “do we get the same predicted concentration value if we compare values of the same well but measured with different gains?”

Thus, performing a Measurement System Analysis (MSA) that integrates both L&BA and R&RA, lets PLATERO not only to measure the uncertainty that should be expected from the measurements, but also check and validate the calibration model, comparing the reproducibility and repeatability terms.

#### 2.4.1 Linearity and bias analysis

The accuracy of a measurement system (more specifically referred to as *bias*) reflects the difference between the observed measurements and the corresponding *true* values. Besides, the linearity of the measurement system reflects differences in bias over the range of measurements made by the system. We consider a simple model for bias:

$$Bias=\hat{C}-C_{T}=d_{0}+d_{1}\cdot C_{T}$$ (7)

where $\hat{C}$ is the predicted concentration value given by Eq. 6, *C*_*T*_ is the *true* value of the concentration obtained from a master or gold standard, *d*_0_ the intercept, and *d*_1_ the slope of the model.

PLATERO evaluates Eq. 7 for *I* wells measured *K* times each one, using *J* different device gains for each well, and *L* different concentration levels. Therefore, there will be *I* × *J* × *K* × *L* individual measurements in an experiment. Using these values, the equation parameters are estimated using the functions provided by PLATERO (Section 3.1). The number of degrees of freedom (*DF*) of the error after fitting Eq. 7 is also stored as part of the model estimates, because it will be used in Eq. 11 to obtain the t-Student statistics (*t*_*DF*,*α*/2_).

Once the parameters from Eq. 7 have been estimated, the linearity and bias contributions, *%Linearity* and *%Bias* respectively, are calculated to evaluate their relevance as:

$$linearity=Variation\cdot d_{1}$$ (8)

$$\%Linearity=\frac{linearity}{Variation}\cdot 100=d_{1}\cdot 100$$ (9)

$$\%Bias=\overset{L}{\underset{l=1}{\sum }}\frac{Bias_{l}}{Variation_{l}}/L$$ (10)

where *Bias*_*l*_ is the average of Bias for the *l*th concentration level, *Variation*_*l*_ is the $6\cdot {\hat{\sigma }}_{\mathrm{total}}$ for the *l*th concentration level (from the R&R analysis in Section 2.4.2), and *L* is the total number of concentration levels. All terms from Eqs 7–10 can be estimated by the function *biasanalysis.m*.

Modeling the bias as in Eq. 7 is also a necessary step in order to consider uncertainty in the predictions. The (1 − *α*) ⋅ 100% confidence interval (*CI*_*C*_) for a given concentration prediction $\hat{C}$ is calculated by function *cipred.m*, using the expression:

$$CI_{C}=\hat{C}\pm t_{DF,\alpha /2}\cdot s_{\mathrm{Bias}}$$ (11)

where *t*_*DF*,*α*/2_ is the (1 − *α*) percentile of a t-Student distribution with *DF* degrees of freedom (degrees of freedom of the error from the linear model in Eq. 7) and *s*_*Bias*_ is the estimated standard deviation of *Bias* (Eq. 7).

Note that Eq. 11 is assuming that the uncertainty for the predictions is the same for all concentrations (i.e., homoscedasticity). However, it is usual to find that the variance for the predictions is different across concentrations (i.e., heteroscedasticity). This proportional relationship between the bias and the magnitude of the measurements was reported as well in Fedorec et al. (2020). In the case of having a proportional relationship between the error and the magnitude being measured, the heteroscedasticity can be easily neutralized by normalizing the bias values with the observed concentration level in the calibration data set using the following equation:

$$Bias\cdot \frac{1}{C_{T}}=d_{0}\cdot \frac{1}{C_{T}}+d_{1}$$ (12)

The aforementioned scaling affects the calculation of the confidence intervals. The Eq. 11 is rewritten as:

$$CI_{C}=\hat{C}\pm t_{DF,\alpha /2}\cdot s_{\mathrm{Bias}}\cdot \hat{C}$$ (13)

where *s*_*Bias*_ is the estimated standard deviation of the scaled bias, calculated with Eq. 7. The last term in Eq. 13 is the concentration value that undoes the scaling of the bias, and gives to the confidence interval, the amplitude corresponding to a particular concentration level. Ideally, this should be the true concentration level *C*_*T*_. However, in a context of model exploitation, when the true concentration values will remain unknown, the predicted concentration $\left(\hat{C}\right)$ is used.

#### 2.4.2 R & R analysis

Generally, the total observed experimental variability $\sigma_{T}^{2}$ is the sum of the *part-to-part* variability of the measured magnitude $\left(\sigma_{\mathrm{P2P}}^{2}\right)$ , and the inherent variability arising from measurement errors $\left(\sigma_{MS}^{2}\right)$ . In our case, $\sigma_{\mathrm{P2P}}^{2}$ arises when different plate wells containing the same concentration yield different measurements of fluorescence. This can be explained by the stochastic component of the biochemical reactions taking place within the well, and also by the intrinsic experimental variability introduced during the preparation of the well plate. By contrast, $\sigma_{MS}^{2}$ comes from the measurement device (plate reader) Zanobini et al. (2016); Montgomery (2020). In turn, the measurement system has two sources of variability: *i*) the variance due to lack of repeatability $\sigma_{\mathrm{Repeat}}^{2}$ (observed variability when repeating the same measurement), and *ii*) the variance coming from the lack of reproducibility, $\sigma_{\mathrm{Reprod}}^{2}$ (observed variability when the same well is measured under different gains). This can be expressed mathematically as:

$$\sigma_{T}^{2}=\sigma_{\mathrm{P2P}}^{2}+\sigma_{MS}^{2}=\sigma_{\mathrm{P2P}}^{2}+\sigma_{\mathrm{Repeat}}^{2}+\sigma_{\mathrm{Reprod}}^{2}$$ (14)

The purposes of the R& R analysis (R& RA) are:

1. Determine how much of the total variability is generated by the measurement instrument.
2. Isolate the measurement system components of variability (i.e. $\sigma_{\mathrm{Repeat}}^{2}$ and $\sigma_{\mathrm{Reprod}}^{2}$ ).
3. Assess whether the measurement instrument is suitable for the intended application or not.

The R& RA isolates all the components of variability from Eq. 14 and estimates them individually using Design of Experiments (DOE) and Analysis of Variance (ANOVA).

In our work, the analyzed data come from a DOE with two factors involved: the well (W) and the device gain (G) at which the fluorescence values were measured. Consider the measurement instrument measures *I* wells, at *J* gains for *K* repetitions. The statistical model that describes the sources of variability is represented as follows:

$$y_{\mathrm{ijk}}=\mu +W_{i}+G_{j}+{\left(WG\right)}_{ij}+\varepsilon_{\mathrm{ijk}}\left\{\begin{array}{c} i=1,2,\ldots ,I\;\\ j=1,2,\ldots ,J\;\\ k=1,2,\ldots ,K\; \end{array}\right.$$ (15)

where *y*_*ijk*_ is an individual measurement of fluorescence, *μ* denotes the general mean, *W*_*i*_, *G*_*j*_ and ${\left(WG\right)}_{ij}$ are independent random variables accounting for the effect of the well, the gain, and the interaction between well and gain, respectively, and *ɛ*_*ijk*_ is an independent random variable that represents the random error.

If each variable *W*_*i*_, *G*_*j*_, ${\left(WG\right)}_{ij}$ and *ɛ*_*ijk*_ is normally distributed variables with zero mean and variance defined as:

$$\mathrm{var}\left(W_{i}\right)=\sigma_{W}^{2}$$ (16)

$$\mathrm{var}\left(G_{j}\right)=\sigma_{G}^{2}$$ (17)

$$\mathrm{var}\left(WG_{ij}\right)=\sigma_{WG}^{2}$$ (18)

$$\mathrm{var}\left(y_{\mathrm{ijk}}\right)=\sigma_{T}^{2}=\sigma_{W}^{2}+\sigma_{G}^{2}+\sigma_{WG}^{2}+\sigma^{2}$$ (19)

It is possible to estimate each of the variance components using ANOVA as shown in Zanobini et al. (2016). The variability of the wells $\sigma_{\mathrm{P2P}}^{2}$ corresponds to $\sigma_{W}^{2}$ , $\sigma_{\mathrm{Repeat}}^{2}$ corresponds to the random error *σ* ^2^, and $\sigma_{\mathrm{Reprod}}^{2}$ corresponds to $\sigma_{G}^{2}+\sigma_{WG}^{2}$ . Thus, the total variability $\sigma_{T}^{2}$ is estimated as:

$${\hat{\sigma }}_{T}^{2}={\hat{\sigma }}_{\mathrm{P2P}}^{2}+{\hat{\sigma }}_{MS}^{2}={\hat{\sigma }}_{\mathrm{P2P}}^{2}+{\hat{\sigma }}_{\mathrm{Repeat}}^{2}+{\hat{\sigma }}_{\mathrm{Reprod}}^{2}={\hat{\sigma }}_{W}^{2}+{\hat{\sigma }}_{G}^{2}+{\hat{\sigma }}_{WG}^{2}+{\hat{\sigma }}^{2}$$ (20)

Once we have estimated the variability of each isolated component, it is possible to calculate the respective contribution to the total variability:

$$Cont\left({\hat{\sigma }}_{MS}^{2}\right)={\hat{\sigma }}_{MS}^{2}/{\hat{\sigma }}_{T}^{2}$$ (21)

$$Cont\left({\hat{\sigma }}_{\mathrm{Repeat}}^{2}\right)={\hat{\sigma }}_{\mathrm{Repeat}}^{2}/{\hat{\sigma }}_{T}^{2}$$ (22)

$$Cont\left({\hat{\sigma }}_{\mathrm{Reprod}}^{2}\right)={\hat{\sigma }}_{\mathrm{Reprod}}^{2}/{\hat{\sigma }}_{T}^{2}$$ (23)

$$Cont\left({\hat{\sigma }}_{\mathrm{P2P}}^{2}\right)={\hat{\sigma }}_{\mathrm{P2P}}^{2}/{\hat{\sigma }}_{T}^{2}$$ (24)

Note that the R& R analysis will be carried out on the predicted concentration values from Eq. 6. Hence, when we report the performance of the measurement system, we will be including the unit conversion operation as part of the measurement system, as illustrated in Figure 2. Thus, the results obtained in this analysis will serve as a part of the validation of the units conversion model proposed in Eq. 6.

## 3 Results

This section goes through the different steps of PLATERO’s calibration protocol in a tutorial-like style, showing how to apply it. To this end, we used two different plate readers. Notice the values of parameters in this section, and the results of the evaluation of the variability obtained, are particular to the plate readers we used. The goal of the section is to show how PLATERO is applied, and the kind of results and analysis that can be drawn for its application.

The sections is divided into four main parts. Section 3.1 describes the results obtained from using PLATERO with a fluorescein calibration dataset, estimating the coefficients in Eq. 6 and Eq. 11 to predict the fluorescein concentration of the plate wells. Next, Section 3.2 assesses on the validity of the gain effect function *f*_*G*_ in Eq. 2, showing the results obtained from an hypothetical scenario where an incorrect gain effect function *f*_*G*_ is assumed. Finally, Section 3.3 describes the results when the expressions fitted in Section 3.1 are applied to predict the concentration of samples with fluorescein concentration out of the calibration range. Finally, Section 3.4 gives the results obtained for a second plate reader, showing how in this case PLATERO warned of problems related to the consistency of measurements caused by the device. The datasets we used in all cases, and the Matlab scripts running PLATERO on these datasets can be obtained from the Zenodo repository González-Cebrían et al. (2022).

### 3.1 Model building and validation

This is a two-step procedure, as depicted in Figure 2. It is the first task a user of PLATERO must carry out before exploiting the calibration model with fluorescence measurements from cells expressing any fluorescent reporter, in this case, Green Fluorescent Protein (GFP). The calibration experimental protocol was executed following the steps detailed in Section 2.1, and yielding a data set with fluorescein measurements.

We used the dataset obtained from the first plate reader González-Cebrían et al. (2022). This dataset was divided into two subsets: one for the Model Building process, and another one for the Model Validation. In the following sections, we will show the results obtained with our particular data set in the Model Building (Section 3.1.1) and Model Validation (Section 3.1.2) steps.

#### 3.1.1 Model building

The first step a user of PLATERO must carry out is the Model Building. The calibration model can be obtained by executing the function *fit*_*calibration*_*model.m* of the PLATERO toolbox on the model building dataset. In our case, the model building dataset contained approximately 70% of the wells with fluorescein, chosen by random selection. That is, we used 1,056 of *F*_*observed*_ (3 concentrations × 4 gains × 11 wells × 8 repetitions). The random selection prevents potential location effects due to the selection of wells in a specific rows or columns order.

First, the gain effect model (*f*_*G*_) was fitted for each set of four *F*_*observed*_ values acquired from each well at each repetition. Thus, 264 estimates (N = 8 repetitions×11 wells×3 concentrations) for the coefficients *b*_1_ and *b*_2_ in Eq. 4 were obtained. This approach was preferred instead of having one single estimate for each parameter in *f*_*G*_ because we wanted to assess their stability across the different wells and concentrations. Further details about the results on this assessment can be seen in Supplementary Appendix S2.

Median values for *b*_1_ and *b*_2_ (Table 1) were finally considered as the global estimates of the gain effect in the fluorescence measurements *F*_*reporter*_ from Eq. 4. These values are part of the final calibration model (Eq. 25).

**TABLE 1:** Coefficients of the units conversion model.

***F***_***BLK,G***_				***b***_***1***_	***b***_***2***_	***c***_***0***_	***c***_***1***_	***s***_***Bias***_	***DF***
_***G = 50***_	_***G = 60***_	_***G = 70***_	_***G = 80***_						
4	10	26	65	0.24298	−0.0933 ⋅ 10^−3^	−1.1185 ⋅ 10^−3^	1.0576	0.0225	1,054

**FIGURE 3:**
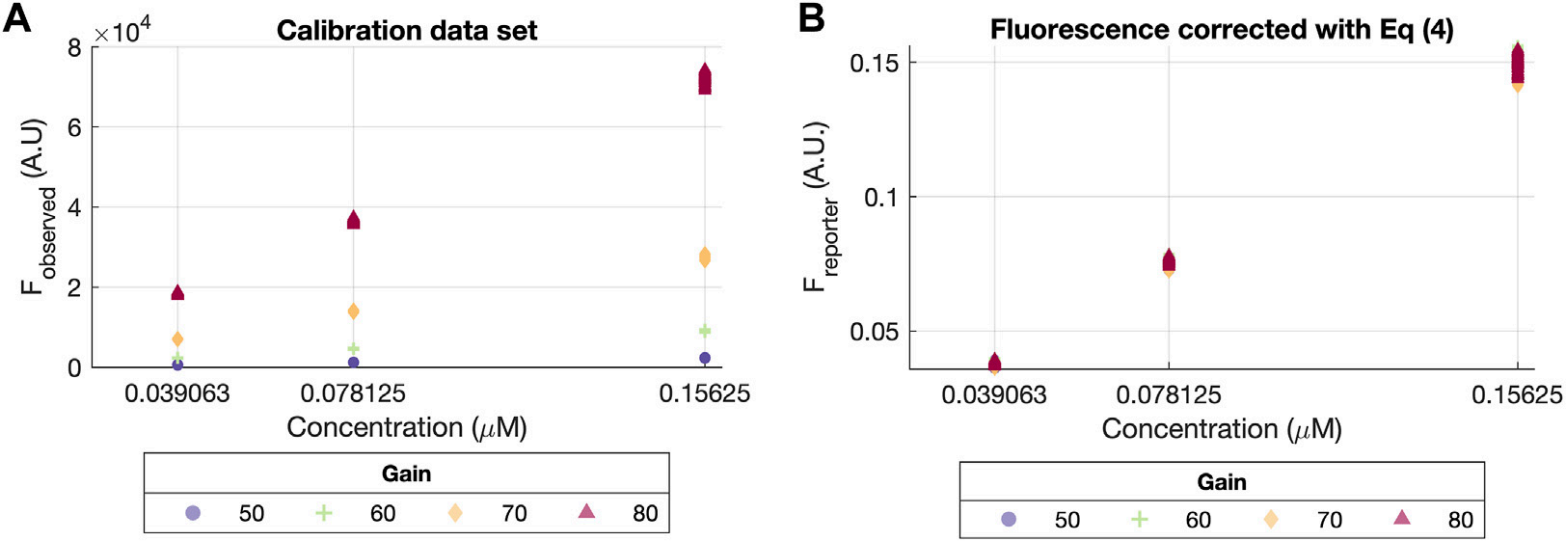
Calibration data set before and after correction with Eq. 4. **(A)** Scatter plot of concentration vs. the raw original values (*F*_*observed*_). **(B)** Scatter plot of concentration vs. corrected fluorescence values (*F*_*reporter*_) using Eq. 4, assuming the exponential gain effect from Eq. 2, with a dashed line indicating the linear relationship described by the corrected values.

Figure 3 depicts the difference between the data before and after removing the gain effect *f*_*G*_ (left and right plots, respectively). In Figure 3A, the observed fluorescence (*F*_*observed*_) at different gain values (ranging from G = 50 to G = 80) has a clear non-linear effect with respect to each fluorescein concentration. On the contrary, this effect disappears in Figure 3B, where all data points describe the same linear relationship between the fluorescence *F*_*reporter*_ at each concentrations values, regardless of the gain.

The second step is to fit the units conversion model, *f*_*UC*_ to obtain fluorescence data expressed in standard fluorescent units. PLATERO automatically fits the units conversion model using the function *f*_*UC*_ and the estimated parameters for Eq. 5. The resulting model for this first plate reader was:

$$\hat{C}=-1.1185\cdot 10^{-3}+1.0576\cdot \left(F_{\mathrm{observed}}-F_{\mathrm{BLK,G}}\right)\cdot e^{-0.24298\cdot G+9.933\cdot 10^{-4}\cdot G^{2}}$$ (25)

where *F*_*BLK,G=50*_ = 4, *F*_*BLK,G=60*_ = 10, *F*_*BLK,G=70*_ = 26 and *F*_*BLK,G=80*_ = 65.

**FIGURE 4:**
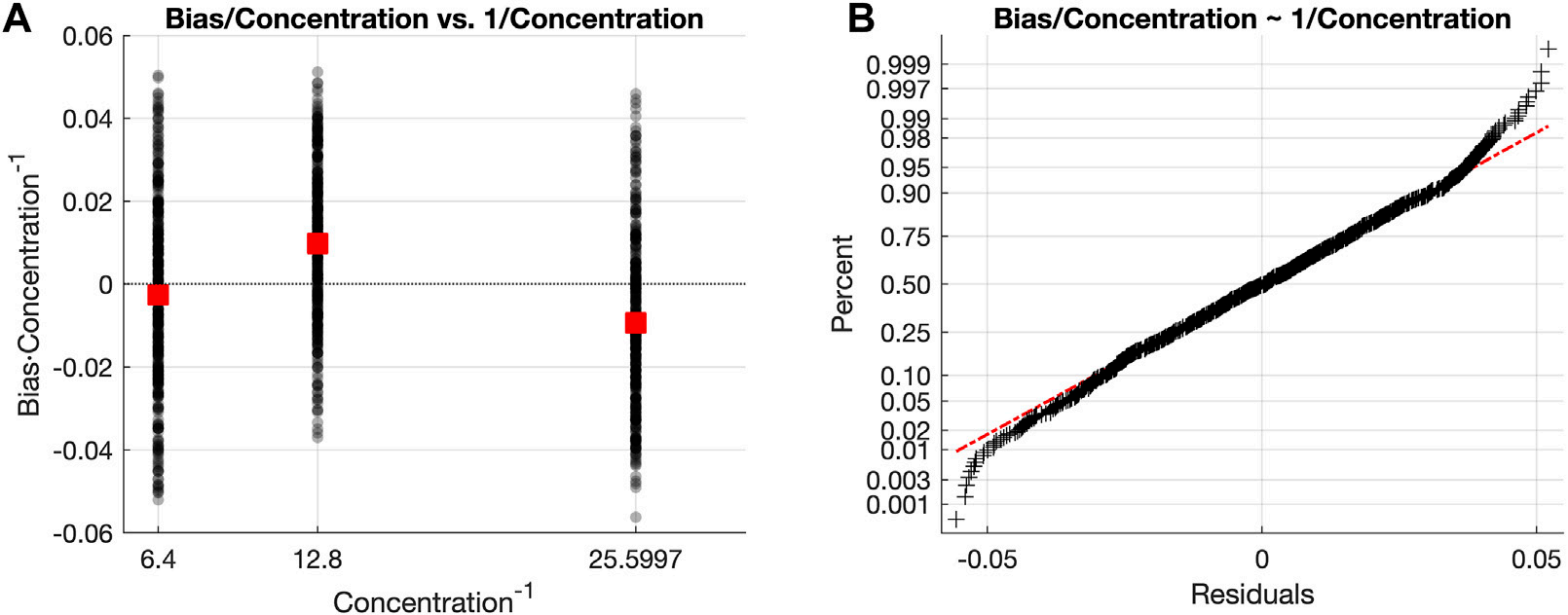
**(A)** Scatter plot of the scaled bias values, where black dots represent every scaled bias value and red squares are the mean scaled bias value for each concentration level. **(B)** Normal probability plot of the residuals quantifying the uncertainty using the scaling of the residuals (Eq. 12) by the concentration values. The red dashed line represents the ideal curve described by residuals perfectly following a normal distribution. Black crosses are the scaled residuals for every data point in the Model Building subset.

Now, for a given value of fluorescence (*F*_*observed*_) measured at any gain (*G*), we can provide a prediction of the fluorescence concentration $\hat{C}$ in standard units. However, the fluorescence values in Figure 3 show some variability despite belonging to the same concentration or gain level, for either *F*_*observed*_ (A) or *F*_*reporter*_ (B). This is a consequence of the inherent experimental variability, and we have to take it into account to provide more confident predictions. To do so, we applied Eq. 25 to the Model Building dataset and analyzed the error (bias) between the estimated concentration $\hat{C}$ and the true concentration values *C*_*T*_. The Supplementary Appendix S3 contains a further assessment comparing the results obtained with the bias model from Eq. 7, and with the scaled bias model from Eq. 12. Figures 4A, 4B illustrate the dispersion and the normal probability plot of the scaled residuals, respectively, obtained with Eq. 12. As it can be seen, the normal probability plot fits the line of the normal distribution, validating the use of the scaled bias model from Eq. 12.

The last step is to estimate the *s*_*Bias*_ term as the standard deviation of the scaled bias. This resulted in the following expression (for the case of our particular calibration dataset) to compute the confidence intervals for the estimated concentration $\hat{C}$ , at a (1 − *α*) ⋅ 100% confidence level:

$$CI_{C}=\hat{C}\pm t_{\mathrm{1054,}\alpha /2}\cdot 0.0225\cdot \hat{C}$$ (26)

where $\hat{C}$ is the concentration prediction from Eq. 25, and *t*_*1054,α∕2*_ is a t-Student statistic automatically calculated by the *cipred.m* function.

To sum up, Table 1 contains all the estimates for the parameters obtained in the Model Building step.

#### 3.1.2 Model validation

The calibration model fitted in the previous Section 3.1.1 was used to predict the fluorescein concentration levels using the Model Validation data set (Figure 2). Particularly, these predicted concentration values were used:

• to carry out an R&R analysis assessing the sources of variability affecting the predictions for each concentration level;
• to perform a B&L analysis assessing the variability of the scaled residuals across the range of concentration levels; and
• to validate if the confidence intervals of such predictions contained the true fluorescein concentration value.

##### 3.1.2.1 R&R analysis

These analyses (one for each concentration level) seek to evaluate how the experimental conditions (well location, well volume, the gain of the device, or the number of repetitions of a measurement) relate to the differences observed between the values of the preictions of concentration, $\hat{C}$ .

The R&R analysis decomposes the total variability seen in $\hat{C}$ into different sources of variability as the result of different experimental factors: (1) the “Part-to-Part” component $\left(\sigma_{\mathrm{P2P}}^{2}\right)$ associated with differences among the wells, (2) the “Reproducibility” component $\left(\sigma_{\mathrm{Reprod}}^{2}\right)$ generated by the differences related to measuring at different gain values, and (3) the “Repeatability” component $\left(\sigma_{\mathrm{Repeat}}^{2}\right)$ that is associated to differences seen between repetitions of the measurements.

**FIGURE 5:**
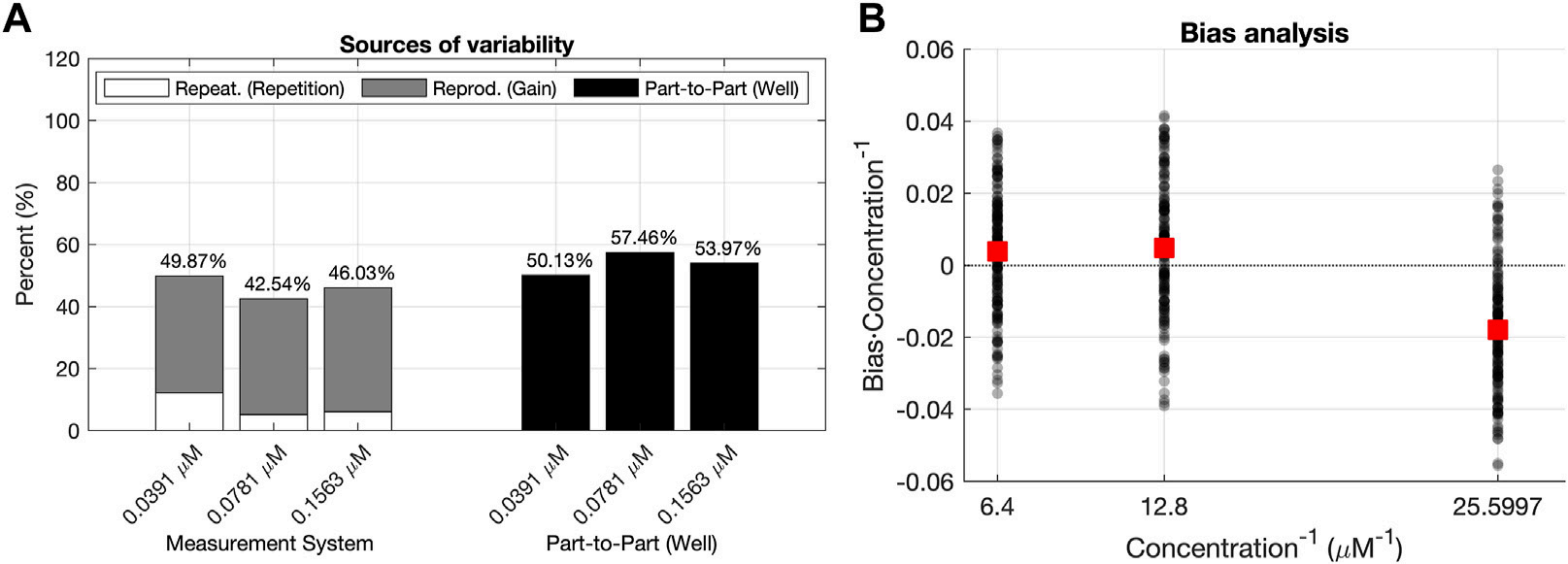
Analyses of the validation data set. **(A)** R&R analysis for the validation dataset. The total variability is decomposed into the Part–to–Part $\left(\sigma_{\mathrm{P2P}}^{2}\right)$ and the Measurement System contribution. In turn, the Measurement System contribution contains both Repeatability plus Reproducibility values. The Part-to-Part variability represents the differences between $\hat{C}$ values for different wells with the same fluorescein concentration. The Reproducibility (Gain) variability $\left(\sigma_{\mathrm{Reprod}}^{2}\right)$ represents the differences between concentration values for the same measurement recorded at different gain levels. **(B)** L&B analysis for the validation data set, illustrating the scattering of the scaled bias values (black dots) for each Concentration^−1^ level. The plot illustrates also if there is a tendency between average values of the scaled bias (red squares) and the Concentration^−1^.

Figure 5 shows the distributions of these three sources of variability for the concentrations analyzed with the data corresponding to the Plate Reader 1. For all concentrations, the part-to-part variability had a higher contribution to the total variability than the reproducibility component. This means that the variability of the predictions of concentration $\hat{C}$ for the same well, even if taken at different gain levels, is lower than the variability of the predictions obtained for different wells measured at the same gain. In other words, the gain effect has been successfully modeled and removed. Interestingly, after the unit conversion process, we are still able to distinguish among the wells that had the same fluorescein concentrations.

##### 3.1.2.2 L&B analysis

The L&B analysis considers the relationship between the scaled *Bias* (Eq. 12), and the predicted fluorescence concentration $\hat{C}$ . This is necessary to evaluate the model proposed in Eq. 7. Studying the statistical features of the prediction error across all concentration levels is a needed step in order to consider any corrections to the proposed model that might be required, as shown in the following results. Figure 4B plots how, for the Plate Reader 1 we used, the *Bias*/*Concentration* values are approximately symmetrically scattered around zero. As seen from the analysis, there is no linear relationship between the scaled bias and the inverse of the concentration level. This means that the relation between the real concentration and the predicted one has been properly captured by the model. Hence, the bias term does not contain any relevant information missed by the model, but noise. This is quantitatively represented by the low contribution (in percentage) of both the linearity (1.5218%) and the bias (7.5792%) terms on the total variability of the scaled residuals (see the outcome of the B& L Analysis in Supplementary Appendix S4). In summary, the linearity and bias of the residuals are not relevant to the total residual variability. Therefore, there is not important information remaining on the prediction errors, i.e.: the model used for the prediction is valid. This is also an indicator of the consistency of the functions *f*_*UC*_ and *f*_*G*_ for all the concentrations within the experimental range of values.

##### 3.1.2.3 Confidence intervals

Finally, it is important to assess the prediction error. Table 2 lists some common metrics for the prediction error: the means squared error (MSE), and the minimum and maximum relative errors. These metrics can be compared to the results obtained from different data sets or with different proposals of unit conversion models.

**TABLE 2:** Performance metrics using the calibration and validation sets after using the exponential and linear *f*_*G*_ (Eqs 4, 27, respectively).

Data set	MSE	MinRE (%)	MaxRE (%)
Calibration	5.6728 ⋅ 10^−6^	0.0040 ⋅ 10^−2^	5.6197
Validation (exponential *f*_*G*_)	3.4285 ⋅ 10^−6^	0.0017 ⋅ 10^−5^	5.5619
Validation (linear *f*_*G*_)	0.0028	9.12	81.76

However, the metrics used in Table 2 are relative metrics and do not explicitly validate if such error values are small enough to assume that the concentration predictions $\hat{C}$ are close enough to the ones known *a priori* *C*_*T*_. To this end, PLATERO’s last step for the validation process is the analysis of the confidence intervals for the predictions. Specifically, the confidence intervals of every predicted concentration $\hat{C}$ were obtained from Eq. 26, using the *t*_*DF*,*α*/2_ and *s*_*Bias*_ from the Model Building step.

**FIGURE 6:**
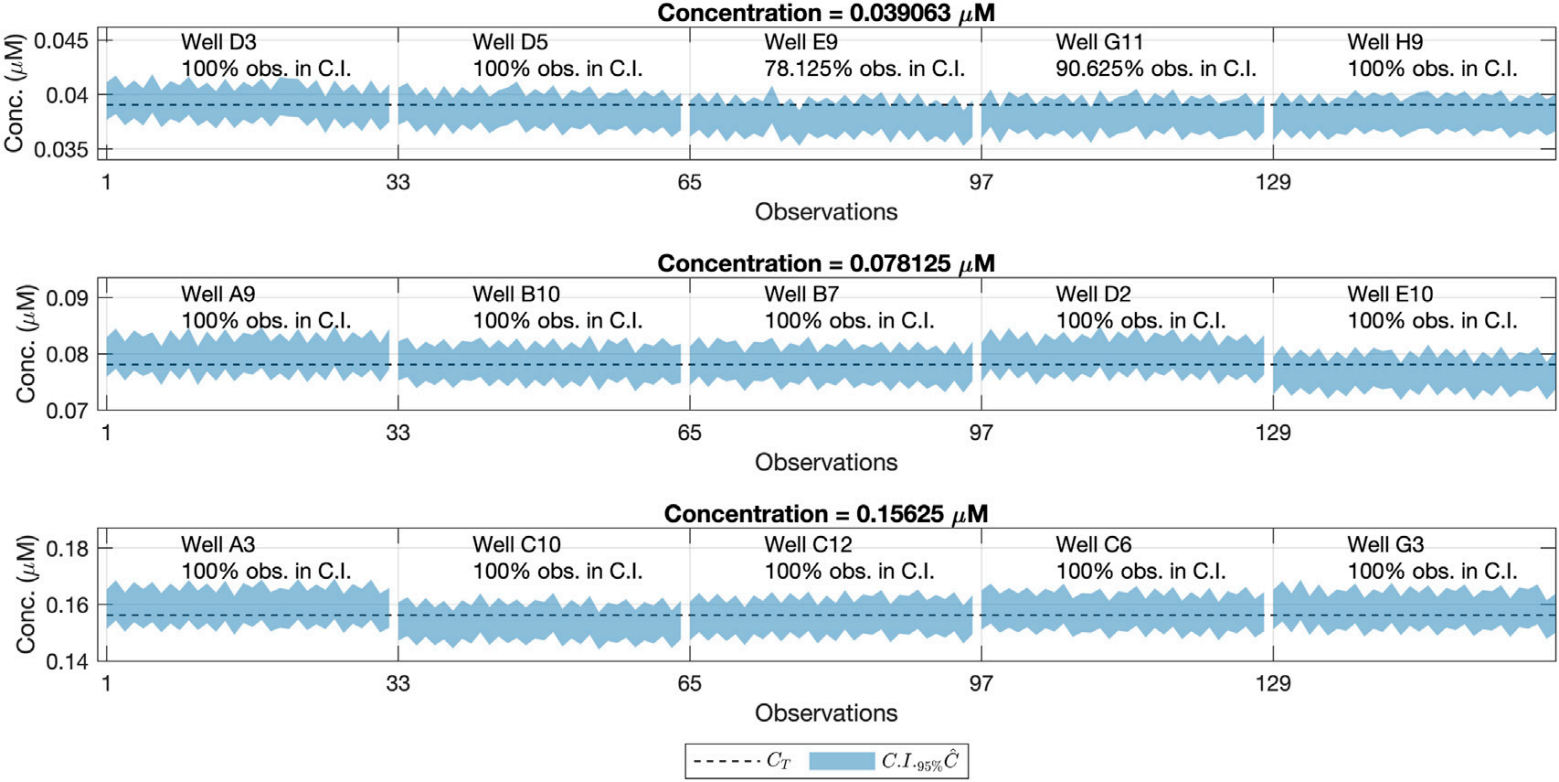
Confidence Intervals (95%) for the concentration values (shaded area) and reference concentration values (dashed line). Each row refers to one concentration level, the same ones as in the Model Building step, measured also at the same gains but from different wells. Each column corresponds to a different well and its location on the 96-well plate. For each well, we had a total of 32 (8 repetitions × 4 gains) predictions of $\hat{C}$ .

If the confidence interval contains the true concentration value *C*_*T*_, the prediction will be valid, i.e., the predicted $\hat{C}$ value is close enough to the true concentration. Figure 6 depicts the true concentration values for every data as dashed lines with the confidence region (blue shaded area) limited by the extremes of the confidence intervals for the predictions. These values were calculated at a 95% confidence level (*α* = 0.05). Figure 6 shows that the confidence intervals for the predictions contain the true concentration value for nearly 95.3125% of the observations in the validation data set.

Notice the wells E9 and G11 (corresponding to *C*_*T*_ = 0.039063) had less than 95% of their confidence intervals containing the true concentration value. The key aspect to deal with this variability is to assess if the differences between the theoretical concentration value and the obtained one, are big enough to consider that a certain well is not a valid replicate for a given concentration level.

We did not consider the results obtained in the wells E9 and G11 invalidated the calibration model. Instead, we hypothesized that the real concentration on those wells was not exactly the theoretical one (*C*_*T*_). This was probably the result of the inherent variability of the experimental procedure. The introduction of the human factor leads to small differences between the theoretical and the real fluorescein concentration that is deposited in the wells.

**FIGURE 7:**
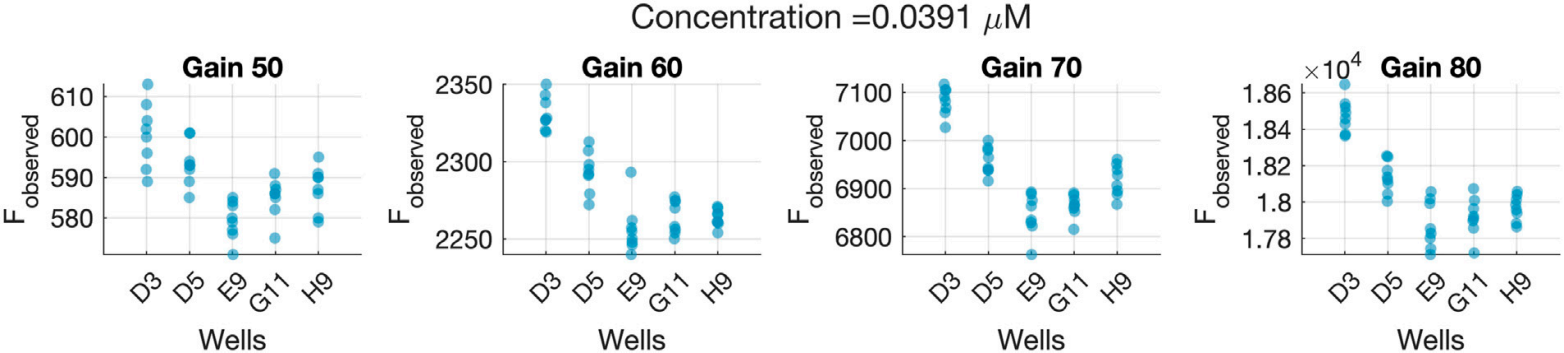
Observed fluorescence values (*F*_*observed*_) for all wells of the validation data set containing the *C*_*T*_ = 0.039063 *μM* concentration level, measured at four different gain levels. The horizontal axes indicate the specific well yielding the *F*_*observed*_ measurements.

This hypothesis was supported by the fact that the same bias was seen in the confidence intervals from wells E9 and G11 in Figure 6. This was also appreciated in the raw fluorescence values *F*_*observed*_ obtained for those wells, as seen in Figure 7. This means that the difference between the real and the assumed concentration was already present in the sample, and it was not introduced by the calibration model.

It is worth mentioning that this variability between wells of the same concentration was already pointed out by the high contribution of the part-to-part $\left(\sigma_{\mathrm{P2P}}^{2}\right)$ variability source in the R&R analysis (Figure 5A). Moreover, this also explains the lower average bias for the lowest concentration level, which in turn, is the highest 1/*Concentration* level obtained in the L&B analysis (Figure 5B).

Finally, the low variability of the reproducibility term $\left(\sigma_{\mathrm{Reprod}}^{2}<\sigma_{\mathrm{P2P}}^{2}\right)$ , together with the low contributions of the linearity and bias terms, and the high percentage of confidence intervals for the expected concentration containing the true concentration value statistically validate the proposed units conversion model and the assumptions from Section 2.3.

### 3.2 Assessment of the gain effect function

In this section, we will address the following question: what if we were using an incorrect *f*_*G*_ expression? This is a very legitimate question since the formal expression of the gain effect is not provided by the plate reader manufacturer. In this section we assumed a widespread correction expression, assuming a linear effect of the gain in the fluorescence (Eq. 27).

$$F_{\mathrm{reporter}}=\frac{F_{\mathrm{observed}}-F_{\mathrm{BLK}}}{G}$$ (27)

**FIGURE 8:**
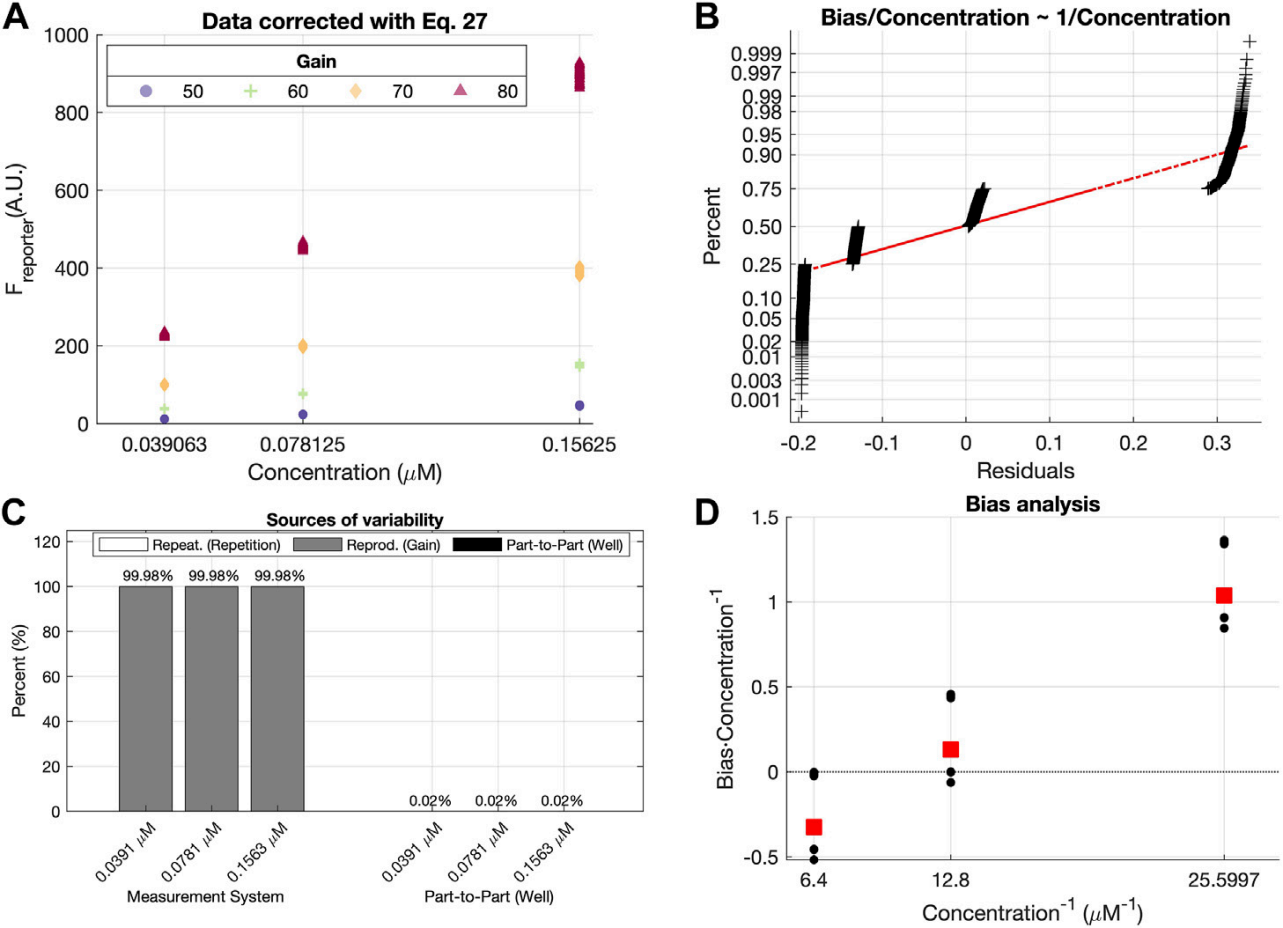
Results obtained with after using Calibration data set before and after correction with Eq. 27. **(A)** *F*_*reporter*_ values assuming a linear effect of the gain on the fluorescence measurements. Marker shapes and colors depend on the Gain level *G* used. **(B)** Normal probability plot of the scaled residuals after using the *f*_*G*_ expression from Eq. 27 to predict the concentration. R&R **(C)** and L&B **(D)** analyses of the validation data set after using the *f*_*G*_ correction from Eq. 27.

Figure 8A shows the result after applying Eq. 27 to the *F*_*reporter*_ values. There are still large differences (up to 3 orders of magnitude) among the values for the same concentration caused by the fact of measuring them at different gain levels. The result seen in Figure 4B is very different from the one in Figure 8B, where values acquired at different gain values are almost indistinguishable after correcting them with the model Eq. 4. Therefore, the gain effect and its linear form remain on the data after using Eq. 27.

The same conclusion is attained by inspecting Figure 8B which illustrates the normal probability plot of the residuals after assuming a linear effect of the gain on the measurements. As it can be seen, residuals are forming small groups, contrary to the distribution seen in Figure 8B, indicating that the gain effect has not been completely removed from the observations.

Both results would be enough to validate the calibration function *f*_*G*_ given by Eqs 2, 4. However, as for the exponential gain effect, R&R and B&L analysis was carried out.

Figure 8C illustrates that the measurement system is the main source of variability among measurements. Specifically, the “Reproducibility (Gain)” term is the one constituting almost 100% of the total variability, indicating that the gain effect correction is not appropriate.

**FIGURE 9:**
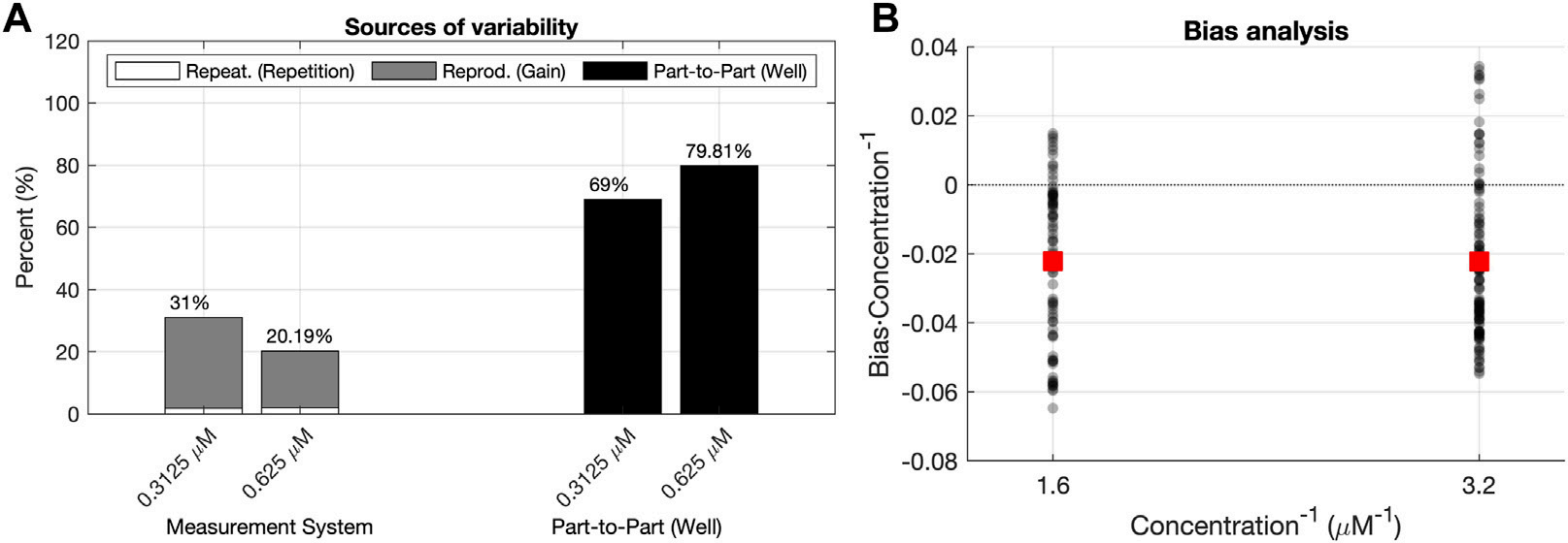
R&R **(A)** and L&B analysis **(B)** for a dataset with concentration values outside the calibration range used for the model building.

The L&B plot (Figure 8D) clearly shows non-null bias values, and also a linear relationship between the scaled bias and the inverse of the concentration. This outcome differs substantially from the one seen in Figure 9, suggesting an incorrect assumption of the gain function *f*_*G*_.

Finally, the metrics shown in Table 2 also provide a comparative corroboration that concludes the improvement of modeling the gain effect as an exponential relationship among the observed fluorescence (*F*_*observed*_), the one emitted by the reporter (*F*_*reporter*_), and the gain used for the measurements (*G*) (Eq. 4). All these results consistently prove that using Eq. 27 is not modeling properly the gain effect on the measurements.

### 3.3 Extrapolation to other concentrations

We also addressed the issue of the goodness of the calibration model outside the concentration range used for the Model Building and Validation steps. Therefore, we used different and higher concentrations of fluorescein as a new dataset. The concentrations we used to test the extrapolation ability of the model were 0.3125 *μM* and 0.625 *μM*. They were not part of the calibration procedure because the fluorescence measurements saturated at the gains of 70 and 80. Figures 9A, 9B show the results of the R&R and L&B analysis for these concentrations.

**FIGURE 10:**
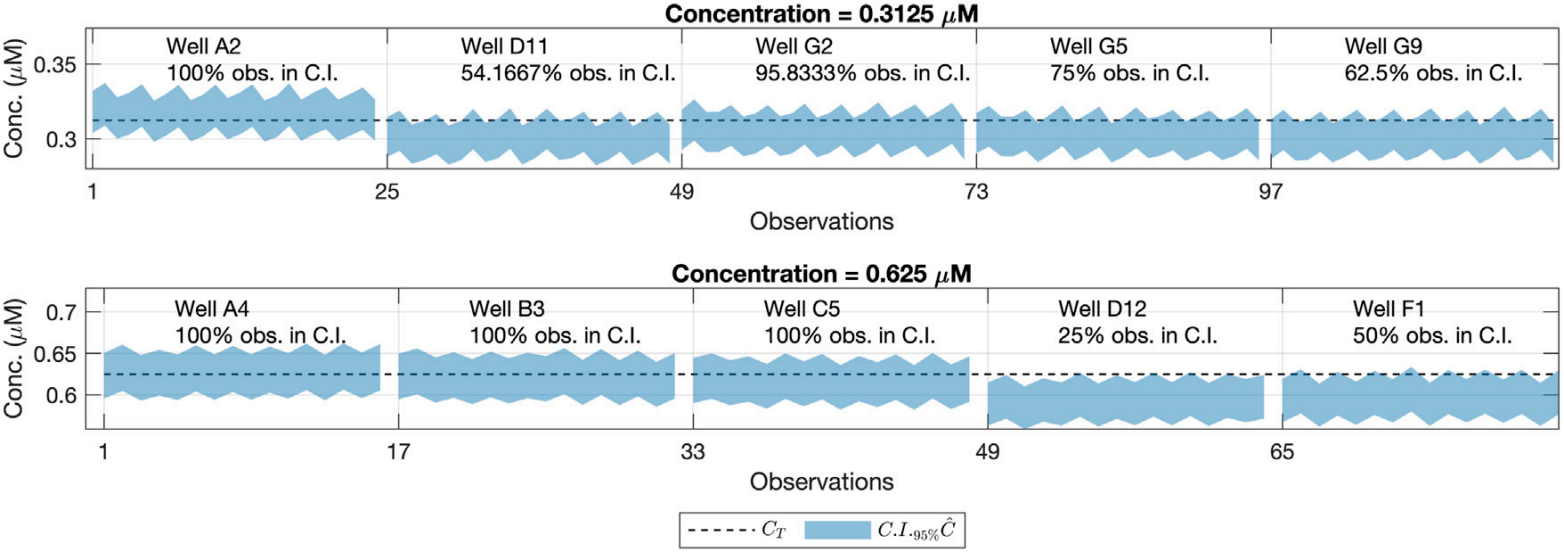
95% Confidence Intervals for the concentration values outside the model buildings’ concentration range (shaded area) and reference concentration values (dashed line). Each row refers to one concentration level. Each column corresponds to a well’s location on the 96-well plate.

As we can see in Figure 9A, the reproducibility (Gain) contribution is still lower than the part-to-part (Well) contribution for both concentrations. Thus, the proposed *f*_*G*_ and *f*_*UC*_ functions are still valid to perform the conversion from fluorescence arbitrary units to standard concentration ones. However, Figure 9B shows a negative bias on average for both concentrations. The same negative bias is also noticed in the confidence intervals for the predicted concentrations of each well (Figure 10). This means that the predicted concentration is consistently lower than the theoretical concentration.

**FIGURE 11:**
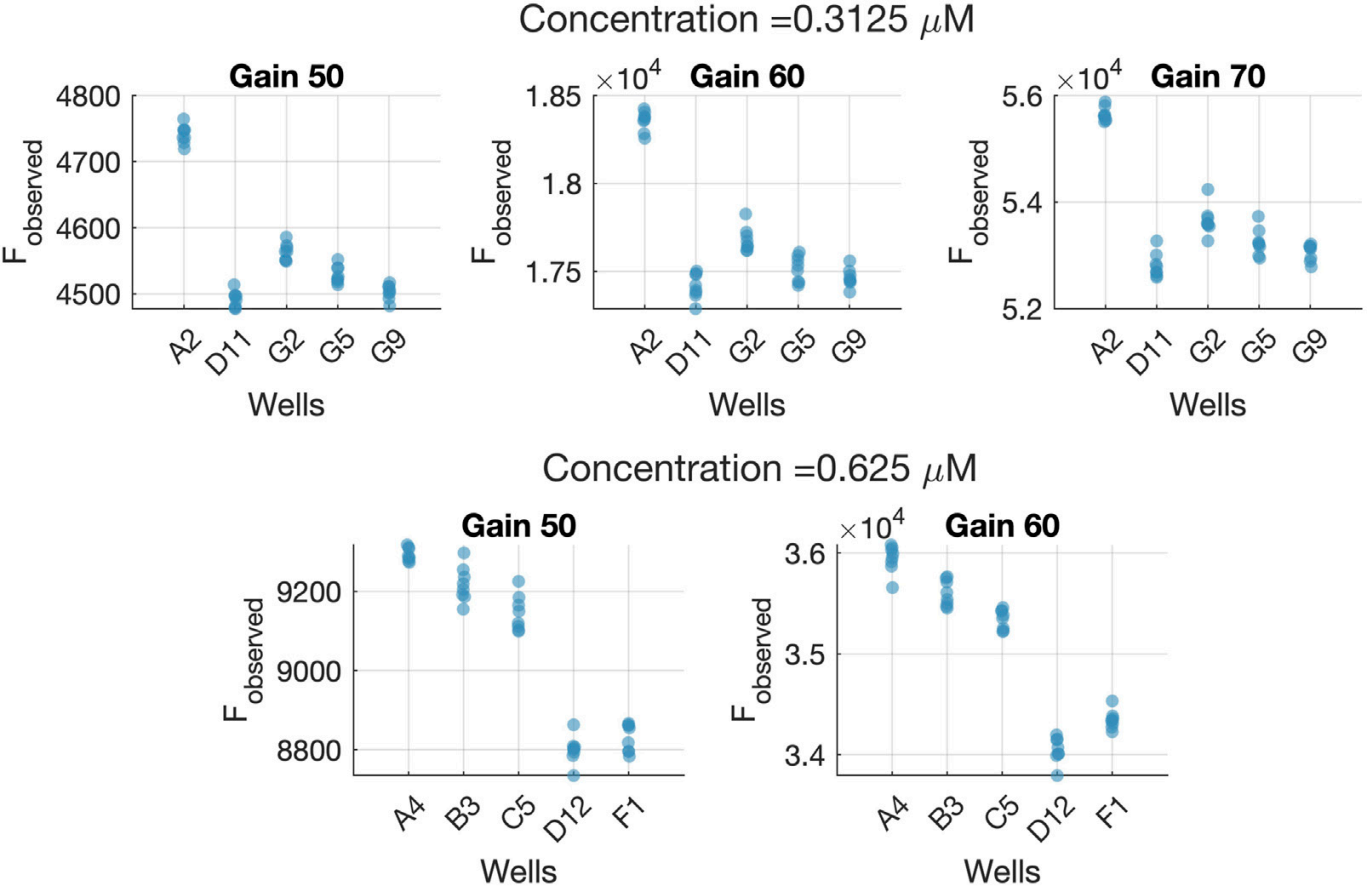
Observed fluorescence values (*F*_*observed*_) for all wells in the 96-well plate with concentration values outside the calibration range used for the model building. Each row refers to one concentration level. Each plot contains the *F*_*observed*_ values recorded at a certain Gain level. The horizontal axes indicate the specific well identity (ID) yielding the *F*_*observed*_ measurements.

In Figure 10, we can see that some of the wells in the 96-well plate with less than 95% of the confidence intervals contained the reference concentration value from the fluorescein pattern. Nevertheless, as said before, the same bias affecting the predictions can be appreciated as well as in the raw fluorescence values (Figure 11).

**FIGURE 12:**
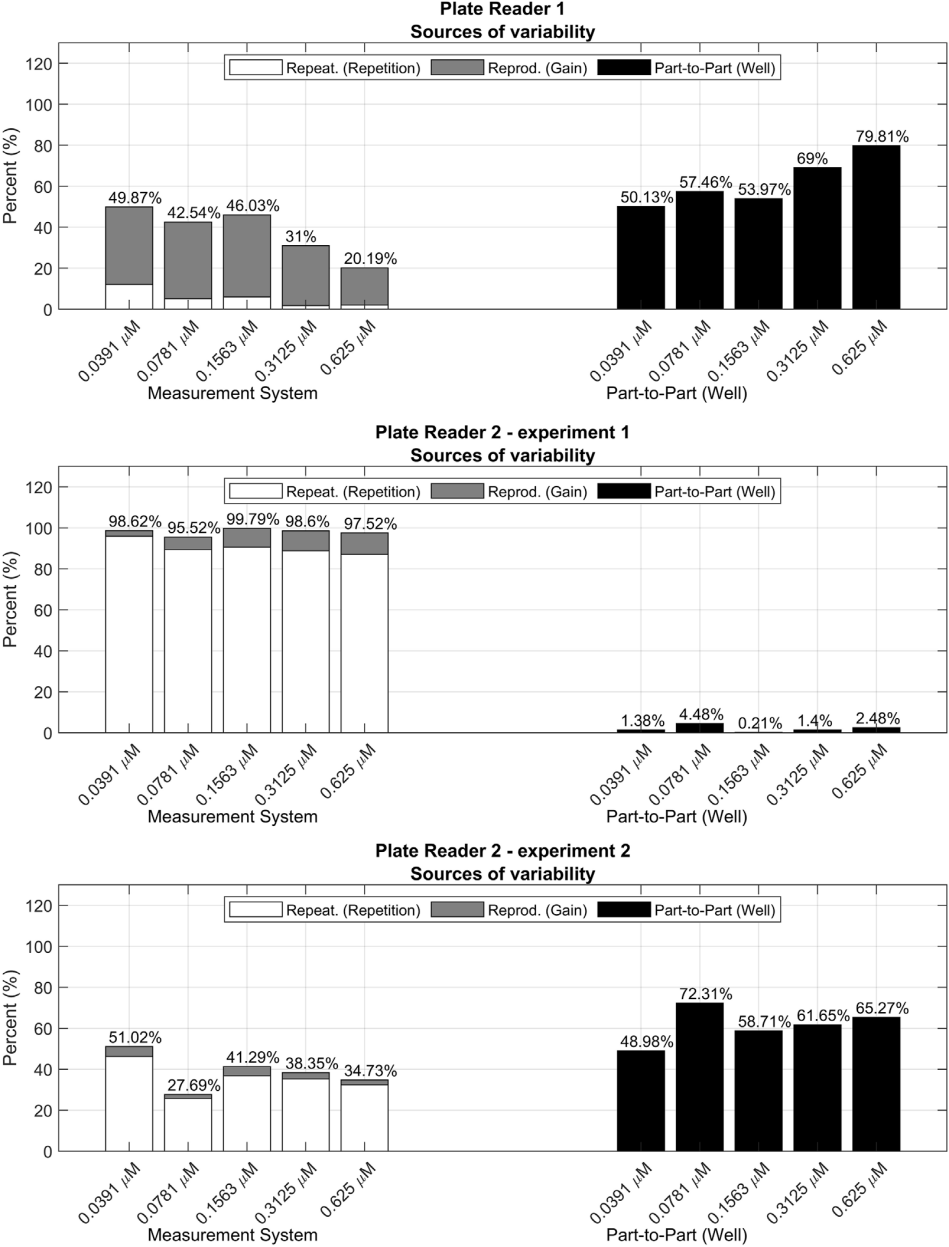
R&R analyses for the validation data sets measured with different plate readers and experimental procedures.

This result may be explained by the fact that most of the variability is due to the part-to-part term (Figure 12), which is close to the 80%, whereas for the concentrations used for the model calibration it was below the 60%. This increment in the part-to-part variability could be due to the presence of outlying wells whose concentration differs from the rest of the wells with the same expected concentration.

In such a case, one would expect a behavior similar to one of wells D12 and F1, with less than the 50% of confidence intervals containing the reference concentration value. This consistent bias seen for wells D12 and F1, remains independent of the gain associated with the measurements, which suggests that the error is associated with the real fluorescein concentration deposited in those specific wells. Note that, this result also illustrates that confidence intervals for the predicted concentration could be used to detect atypical wells in the plate during the calibration step. This also might help to establish statistically significant differences between the predicted concentration within different wells.

In summary, from the results above, we could say that the theoretical expression for the units conversion model could be valid to extrapolate to concentration values outside the calibration range. However, as expected when a model extrapolates, this has some drawbacks and limitations. Consequently, we would recommend including these concentrations for the model building and the estimation of the parameters to provide a better estimate of the uncertainty.

### 3.4 Comparison between plate readers

The following section includes a comparison between three different measurement setups:

• Plate reader 1 (*PR 1*): this setup yielded the data already used along the paper in Section 3.1.1, Section 3.1.2, Section 3.2 and Section 3.3. In this case, all concentrations, even those outside the calibration range used in Section 3.3, were included as part of the validation data set.
• Plate reader 2 experiment 1 (*PR 2 exp. 1*): fluorescence was measured with a different plate reader and modifying a part of the measurement procedure, i.e., the fluorescein dilutions were not mixed between repetitions, increasing the possible effects from photobleaching. The model was fitted with the 70% of the measurements and validated with the remaining 30%. In this case, the gains used to calibrate the model were between 60 and 90, and all concentrations were used for both training and validation.
• Plate reader 2 experiment 2 (*PR 2 exp. 2*): for the third setup PLATERO was executed with data from the plate reader 2, but following the same measurement procedure as in *PR 1.* The model was fitted with the 70% of the measurements and validated with the remaining 30%. In this case, the gains used to calibrate the model were between 60 and 90, and all concentrations were used for both training and validation.

The appendices contain the results obtained after fitting each one of the models. All the data and software to run the tests and obtain the results can be obtained from González-Cebrían et al. (2022). As it can be seen, an acceptable percentage of confidence intervals contained the theoretical concentration (91.62% for *PR 1*, 97.5% for *PR 2 exp. 1*, and 96.38% for *PR 2 exp. 2*). The contributions of the bias and linearity terms to the residuals model are also acceptable for all of them, being at maximum, of a 10%. However, the R&R results (Figure 12; Table 3) point out relevant differences between the measurement systems.

**Table 3:** Variance in measurements obtained with different plate readers.

Concentration	Source	PR 1	PR 2 exp. 1	PR 2 exp. 2
C = 0.0391	Reprod. (Gain)	2, 14*E* − 07	2, 96*E* − 07	6, 89*E* − 07
	Repeat. (Repetition)	6, 92*E* − 08	1, 05*E* − 05	6, 72*E* − 06
	Part-to-Part. (Well)	2, 85*E* − 07	1, 50*E* − 07	7, 12*E* − 06
C = 0.0781	Reprod. (Gain)	1, 00*E* − 06	2, 35*E* − 06	1, 33*E* − 06
	Repeat. (Repetition)	1, 39*E* − 07	3, 43*E* − 05	1, 71*E* − 05
	Part-to-Part. (Well)	1, 54*E* − 06	1, 72*E* − 06	4, 81*E* − 05
C = 0.1563	Reprod. (Gain)	3, 39*E* − 06	1, 15*E* − 05	5, 28*E* − 06
	Repeat. (Repetition)	5, 11*E* − 07	1, 13*E* − 04	4, 31*E* − 05
	Part-to-Part. (Well)	4, 58*E* − 06	2, 61*E* − 07	6, 88*E* − 05
C = 0.3125	Reprod. (Gain)	1, 89*E* − 05	4, 85*E* − 05	1, 65*E* − 05
	Repeat. (Repetition)	1, 16*E* − 06	4, 39*E* − 04	1, 91*E* − 04
	Part-to-Part. (Well)	4, 47*E* − 05	6, 93*E* − 06	3, 34*E* − 04
C = 0.625	Reprod. (Gain)	5, 03*E* − 05	2, 11*E* − 04	6, 69*E* − 05
	Repeat. (Repetition)	5, 41*E* − 06	1, 75*E* − 03	9, 11*E* − 04
	Part-to-Part. (Well)	2, 20*E* − 04	4, 99*E* − 05	1, 84*E* − 03

In Figure 12, the first contribution plot from *PR 1* is illustrating the expected and proper performance of the measurement system. This good quality is reflected by both Repetition’s contributions being the minimal sources of variability and by Measurement System’s contributions being smaller than Part-to-Part’s for all concentrations.

This desirable performance seen for *PR 1* measurements is not maintained in the *PR 2* ones. In general, *PR 2* shows a clear increase of the repeatability contribution to the total variability. In the first experiment (*PR 2 exp. 1*), the well plates were not stirred between repetitions, increasing the variability due to possible photobleaching effects, contrary to the indications of PLATERO’s experimental standard procedure. This apparently small difference, is clearly captured by the R&R analysis, showing an unacceptable contribution of the Measurement System to the total variability generated by a great variability between repetitions.

In the second experiment, *PR2, exp. 2*, the experimental protocol was executed correctly and the balance between Measurement System’s and Part-to-Part’s contributions, are reestablished resembling more the values obtained for *PR 1*. However, the Repeatability contribution to the total variability is still higher than for *PR 1*. Table 3 shows the absolute values of these contributions. As it can be seen, the absolute values of the Gain’s variance term are similar between *PR 1* and *PR 2 exp. 2* but the absolute value of the variance between repetitions is persistently higher for *PR 2*. This result clearly suggests that the plate reader *PR 2* would require a technical revision to evaluate if the low repeatability of its measurements is caused by a deficient maintenance. Otherwise, if this is the expected variability for measurements provided by *PR 2*, this outcome would be a quantitative measure of the difference in quality between plate readers.

In summary, the results yielded by the R&R analysis, may inform users about the need of maintenance for plate readers, and the relative quality of measurements between them. Moreover, as seen for the case of *PR 2 exp. 1*, the R&R analysis should always be included as part of the calibration model’s validation, for they may also detect problems with the experimental protocol.

## 4 Discussion

In this work, we propose a unit conversion model that enables users of fluorescence plate readers to obtain comparable results. The conversion model is a composition of two functions: the gain effect function (*f*_*G*_) and the units conversion function (*f*_*UC*_). The uncertainty around the estimates of the model and its parameters, may vary depending on the machine being used, and the user intervention during the experimental protocol to get experimental data. For this reason, the second pillar of this protocol consists of a Measurement System Analysis (MSA) *via* a R&R and a B&L analysis.

Three real data sets from two different plate readers were obtained following the proposed procedure and used to assess the performance of the calibration model. The results in Section 3.1, showed that over 95% of confidence intervals for the predicted concentration actually contained the true concentration value. Furthermore, as seen in Figure 6 and Figure 10, the confidence intervals can be used to detect wells differing from the expected concentration value, assessing on the quality of the experimental procedure quantitatively. Additionally, Section 3.2 illustrates how the protocol would warn users about the assumption of incorrect gain effect functions. Section 3.3 shows how the model performs when it is used with concentration values above the concentration range of values used to fit the calibration model. Although the model seems to extrapolate fairly well, we would encourage users to re-apply the protocol, and assess the differences in the model parameters when a different range of concentrations is being used. Finally, Section 3.4 illustrates the need to include the R&R analysis, informing users about the quality of the data and potential adjustments required to maintain the quality of plate readers’ measurements, or fix issues during the experimental protocols used to gather the data.

On one hand, the proposal of a single analytical expression for the gain effect (*f*_*G*_) and for the conversion to concentration units (*f*_*UC*_) differs from the FlopR software proposed by Fedorec et al. (2020). The unified mathematical framework proposed in our work is aligned with previous reporting an exponential gain effect as in Castillo-Hair et al. (2016), setting the basis for the comparability between plate readers *via* their conversion coefficients. Yet, beyond the assumption and validation of a universal conversion model, the main contribution of this work is the proposal of a whole methodological framework with a solid statistical basis that can and should be applied to test future proposed analytical expressions. This enables the quantification of the uncertainty in the converted measurements and gives tools for a validation that does not rely in a qualitative comparison between of the variability of the corrected values. This part could be carried to with any of the existing proposals for conversion models and still add value. For this reason, the use of alternative valid analytical proposals for the conversion of units would not devalue or invalidate the methodology and set of tools proposed in this paper. The goal of this work, rather than proposing a unique and universal expression usable with all plate readers and in all experimental conditions, is to pave the way towards the standardization of fluorescence data and to expand the limits of the comparable scenarios in posterior analyses.

In conclusion, the proposal of a single analytical expression to predict concentration from fluorescence values, the estimation of the uncertainty expected on such predictions, and the assessment of the variability of the plate reader’s measurements, bring a solid statistical foundation to the presented work. As far as we know, the integration of these statistical tools is a differential aspect in comparison to other existing proposals to correct the gain effect or to convert fluorescence values to concentration values. Moreover, the calculation of confidence intervals for the predicted concentration constitutes a validation tool for the proposed units conversion model and the overall assumptions underlying it.

Moreover, PLATERO’s approach is so general that it can be used to estimate fluorescence concentrations at any wavelength, provided that the appropriate reference fluorescence solution is chosen. In fact, the underlying model can be extended not only to deal with fluorescence measurements but to absorbance or luminescence measurements.

We believe that tools such as PLATERO (Plate Reader Operator) toolbox will play a key role in Synthetic Biology, enabling the proper comparison of databases coming from different experimental settings, the validation of the quality of the acquired experimental data, and the extension of the measurement system range to a broader one being able to detect more subtle signals but at the same time detecting strong signals.

## Data Availability

The original contributions presented in the study are included in the article/Supplementary Material, further inquiries can be directed to the corresponding author.
