# Supplementary material for: PLATERO: A calibration protocol for plate reader green fluorescence measurements
Source: Front Bioeng Biotechnol. 2023 Jan 20;11:1104445. doi: 10.3389/fbioe.2023.1104445 (PMC9895789; doi:10.3389/fbioe.2023.1104445)
Supplement: Supplementary file 1 [file DataSheet2.pdf]

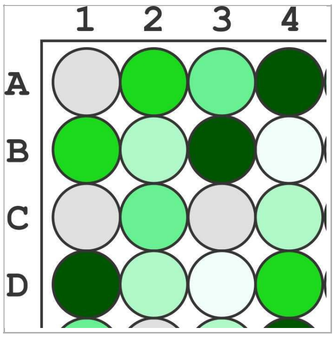

JAN 13, 2023

PLATERO: A Calibration Protocol for Plate Reader Green Fluorescence Measurements

Alejandro Vignoni[1], Yadira Boada[1]

[1]Universitat Politecnica de Valencia

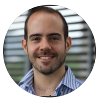

Alejandro Vignoni
Universitat Politecnica de Valencia

OPEN ACCESS

DOI:
dx.doi.org/10.17504/protocols.io.kxygxzjz4v8j/v1

Protocol Citation: Alejandro Vignoni, Yadira Boada 2023. PLATERO: A Calibration Protocol for Plate Reader Green Fluorescence Measurements. **protocols.io** https://dx.doi.org/10.17504/protocols.io.kxygxzjz4v8j/v1

MANUSCRIPT CITATION:
10.3389/fbioe.2023.1104445

License: This is an open access protocol distributed under the terms of the Creative Commons Attribution License, which permits unrestricted use, distribution, and reproduction in any medium, provided the original author and source are credited

Protocol status: Working
We use this protocol and it's working

Created: Aug 02, 2022

Last Modified: Jan 13, 2023

PROTOCOL integer ID:
68080

Keywords: fluorescence calibration, serial dilution

ABSTRACT

One of the most common sources of information in Synthetic Biology is the data coming from plate reader fluorescence measurements. These experiments provide a measure of the light emitted by certain fluorescent molecules, such as the Green Fluorescent Protein (GFP). However, these measurements are generally expressed in arbitrary units and are affected by the measurement device gain. This limits the range of measurements in a single experiment and hampers the comparison of results among experiments. In this work, we provide a calibration protocol to express fluorescence measures in concentration units of a reference fluorophore. The protocol removes the gain effect of the measurement device on the acquired data. In addition, the fluorescence intensity values are transformed to units of concentration using a Fluorescein calibration model. Both steps are expressed in a single mathematical expression which returns normalised, gain independent, and comparable data, even if the acquisition was done at different device gain levels. The protocol embeds a Linearity and Bias Analysis that provides an assessment of the uncertainty of the model estimations, and a Reproducibility and Repeatability analysis that evaluates the sources of variability originating from the measurements and the equipment. All the functions used to build the model, exploit it with new data, and perform the uncertainty and variability assessment are available in an open access repository.

MATERIALS

- Fluorescein Sodium Salt **Contributed by users**
- 1X PBS (Phosphate-buffered saline)

SAFETY WARNINGS

Make sure to record all information about your instrument to document your experiment. If your instrument has variable temperature settings, the instrument temperature should be set to room temperature (approximately 20-25 C) for all measurements.

BEFORE START INSTRUCTIONS

Before beginning these protocols, please ensure that you are familiar with the measurement modes and settings of your instrument. For all of these calibration measurements, you must use the same plates and volumes that you will use in your cell-based assays. You must also use the same settings (e.g., filters or excitation and emission wavelengths) that you will use in your cell-based assays. If you do not use the same plates, volumes, and settings, the calibration will not be valid.

Stock Reference Solution

1 Start from at least 1 mL of [M] 10 micromolar (µM) Fluorescein Sodium Salt **Contributed by users** solution in 1X PBS (Phosphate-buffered saline)

2 [OPTIONAL] If you have access to a spectrophotometer, you can calculate the concentration of your Fluorescein reference stock solution even more accurately using the Beer-Lambert law.

2.1 Measure the solution's absorbance at 492 nm and calculate concentration using an extinction coefficient of 68.029 mM-1 cm-1

2.2 If the concentration of your stock reference solutions is different than [M] 10 micromolar (µM) then you need to recalculate the dilution of the next step to obtain a Dilution 1 solution of [M] 0.625 micromolar (µM)

Prepare the starting dilution of Fluorescein solution

3 Dilute 250 µL of the stock reference solution into 3750 µL of 1X PBS (Phosphate-buffered saline) to obtain 4 mL of Dilution 1 @ [M] 0.625 micromolar (µM)

Prepare the serial dilutions of Fluorescein

4 Accurate pipetting is essential. Serial dilutions will be performed in 5 tubes. There will be a sixth tube that **must contain PBS buffer only**. Initially, you will set up 5 tubes labeled Dilution 2 to 5 and PBS, with 2 mL of

1X PBS (Phosphate-buffered saline) .

Then you will perform a serial dilution by consecutively transferring 2 mL from Dilution 1 to Dilution 2 and so on, always with good mixing (pipetting up and down three times or more).

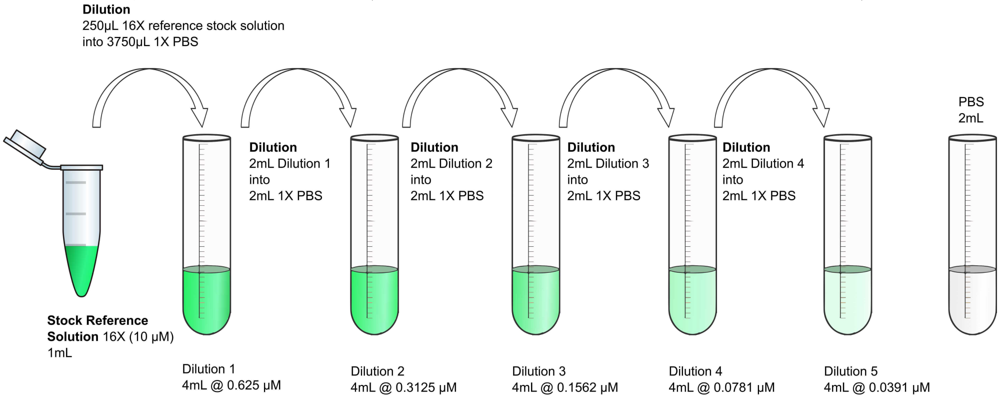

4.1 Label four tubes with the names Dilution 2 to 5, and one tube with the name PBS.

4.2 Add 2 mL of 1X PBS (Phosphate-buffered saline) to all tubes.

4.3 Transfer 2 mL of Dilution 1 into Dilution 2 tube. Mix thoroughly by pipetting up and down three times or more.

4.4 Transfer 2 mL of Dilution 2 into Dilution 3 tube. Mix thoroughly by pipetting up and down three times or more.

4.5 Transfer 2 mL of Dilution 3 into Dilution 4 tube. Mix thoroughly by pipetting up and down three times or more.

4.6 Transfer 2 mL of Dilution 4 into Dilution 5 tube. Mix thoroughly by pipetting up and down three times or more.

5 Transfer 2 mL of Dilution 5 into the liquid waste.

Micro-plate set up

6 Transfer 100 µL of each dilution into the corresponding well using the following plate map:

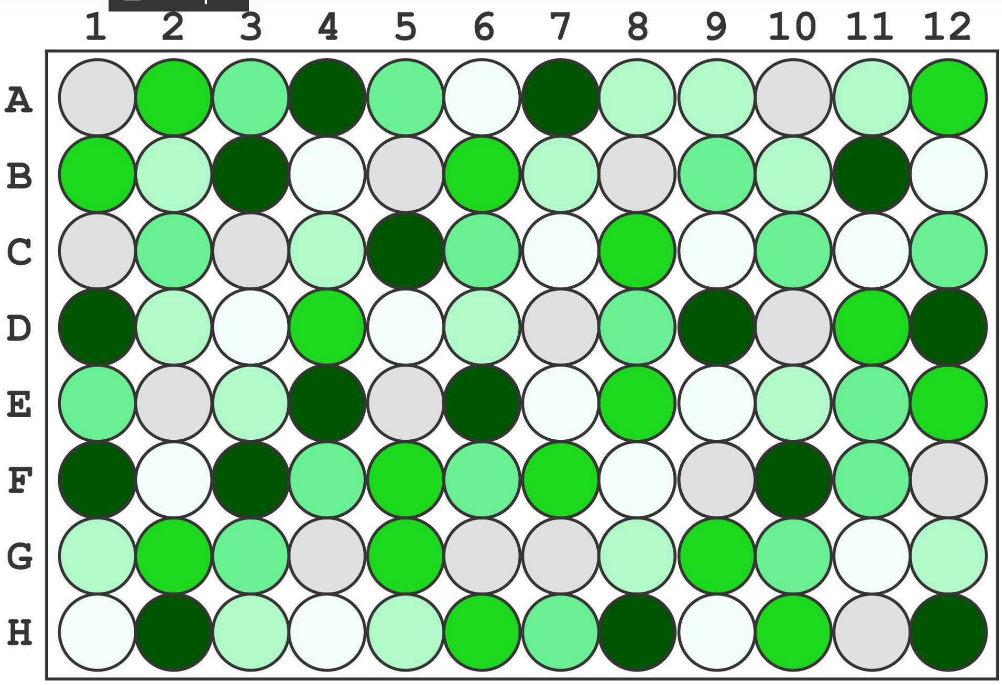

A	B
PBS	A1, A10, B5, B8, C1, C3, D10, D7, E2, E5, F12, F9, G4, G6, G7, H11
Dilution 1	A4, A7, B11, B3, C5, D1, D12, D9, E4, E6, F1, F10, F3, H12, H2, H8
Dilution 2	A12, A2, B1, B6, C8, D11, D4, E12, E8, F5, F7, G2, G5, G9, H10, H6
Dilution 3	A3, A5, B9, C10, C12, C2, C6, D8, E1, E11, F11, F4, F6, G10, G3, H7
Dilution 4	A11, A8, A9, B10, B2, B7, C4, D2, D6, E10, E3, G1, G12, G8, H3, H5
Dilution 5	A6, B12, B4, C11, C7, C9, D3, D5, E7, E9, F2, F8, G11, H1, H4, H9

Note

You can use any random distribution of well you like, this one is just the distribution we used as an example.

Measure Fluorescence

7 Measure the fluorescence of all samples in your plate reader, repeat measurements using 4 different gain settings, and then repeat everything 8 times.

Record data

8 Save your measurements into an excel file with each repetition on a different sheet using the template.

Platero_Data_Template.xlsx
