# Supplementary material for: PLATERO: A calibration protocol for plate reader green fluorescence measurements
Source: Front Bioeng Biotechnol. 2023 Jan 20;11:1104445. doi: 10.3389/fbioe.2023.1104445 (PMC9895789; doi:10.3389/fbioe.2023.1104445)
Supplement: Supplementary file 2 [file DataSheet1.PDF]

Supplementary Material

1 APPENDIX 1

This appendix describes the iterative model search that was carried out to propose the relation between Fluorescence values and gain. It is important to recall the first assumption of the gain effect model, represented by Eq. S1:

$$F_{real} = F_{reporter} + F_{BLK} \tag{S1}$$

Thus, this appendix focuses on inferring the analytical expression f_G: $F_{observed} = f_G(F_{real}, G)$.

At first glance, visualizing Figure 1a, it seems clear that there is not a linear relation between fluorescence values and gain. On the contrary, such non-linear relation appears to be exponential. For that reason, an exponential effect of the gain is initially proposed in Eq. S2.

$$F_{observed} = f_G(F_{real}, G) = F_{real} \cdot e^{b_1 \cdot G} \tag{S2}$$

Then, considering Eqs. S1 , S2 and the third assumption of the gain effect model (i.e., f_G is the same for all fluorescence values), Eq. S3 is deduced:

$$\log(F_{observed} - F_{BLK,G}) = log(F_{real}) + b_1 \cdot G \tag{S3}$$

Note that, Eq. S3 refers to the analytical expression of a linear model. Therefore, if it was an adequate approximation, one would expect to see a linear relation when representing $log(F_{observed} - F_{BLK})$ as a function of G. However, Figure 1b shows a slightly quadratic relation. Hence, it could conceivably be

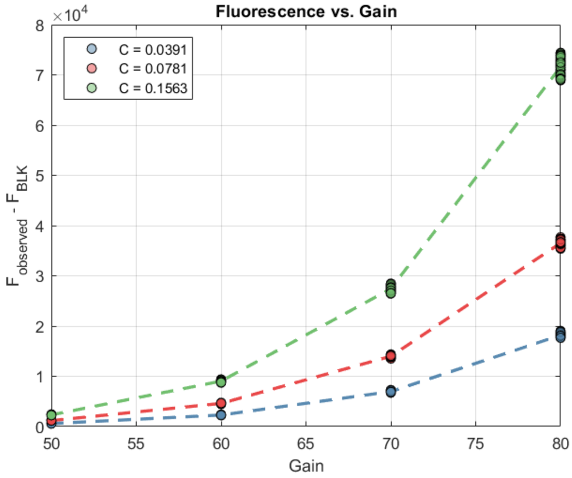

Figure 1a.

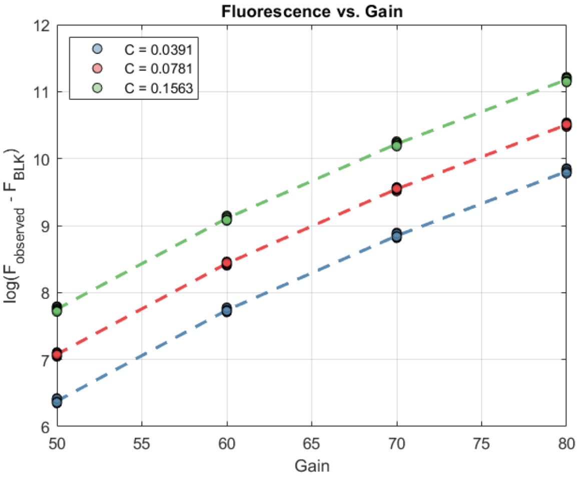

Figure 1b.

Figure 1. Fluorescence values of the calibration data subset for different gains **(A)** and considering a log transformation **(B)**.

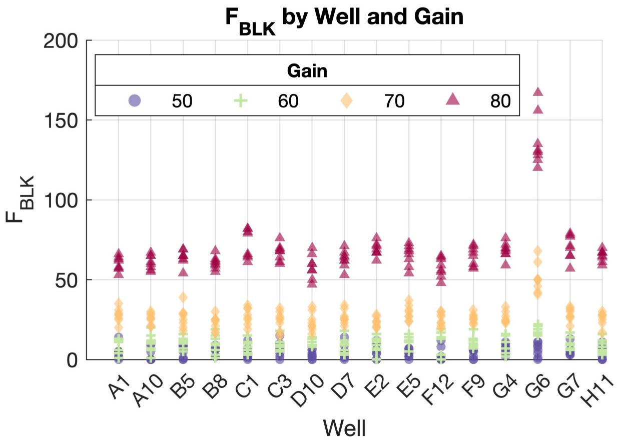

Figure 2a.

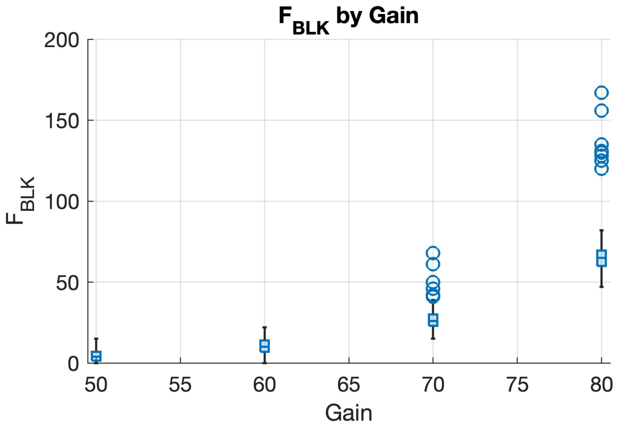

Figure 2b.

Figure 2. Fluorescence values for the wells without fluorescein ($F_{BLK,G}$) used to measure the reader bias by wells and gain **(A)** and just by gain levels **(B)**.

hypothesized of adding a quadratic effect to Eq. S3 in the exponential term given place to Eq. S4. Besides, as the Eq. S2, Eq. S4 can be deduced.

$$\log(F_{observed} - F_{BLK,G}) = log(F_{real}) + b_1 \cdot G + b_2 \cdot G^2 \tag{S4}$$

Thus, according to Eq. S4, a quadratic relation is expected between $log(F_{observed} - F_{BLK})$ and G as desired. For that reason, influence on the real value of fluorescence F_{real} is assumed to be an exponential effect of the gain, with a quadratic term in the exponent (Eq. S4).

2 APPENDIX 2

This appendix describes some intermediate results obtained along the Model Building stage of the Calibration model. They assess the stability of the $F_{BLK,G}$, b_1 and b_2 parameters from f_G (Eq. S4).

The first step is to estimate the $F_{BLK,G}$ terms for each gain level. A this point it is important to acknowledge that working with real data sets may imply the existence of potential outliers due to measurement errors or other issues.

As it can be seen in Figure 2 (A), some observations (well G6) had higher fluorescence measurements than the majority of the values acquired at the same gain. In order to prevent the influence of these potential outliers in the estimation of $F_{BLK,G}$ in Eq. S4, they were calculated as the median fluorescence of the empty wells for each gain level. These were used to correct the additive noise introduced by medium.

Afterwards, in order to check if b_1 and b_2 were significantly consistent for all levels of concentration, a nested arrangement employed to estimate components of variance is used, called hierarchical design George et al. (2005). Thus, the different wells are hierarchically subsumed under the levels of concentration. The associated ANOVA table is shown below.

Analysis of Variance

Source	Sum Sq.	d.f.	Mean Sq.	F	Prob>F
Concentration	0	2	1.1491e-06	0.13	0.874
Well(Concentration)	0.00035	30	1.16589e-05	1.37	0.1049
Error	0.00197	231	8.52553e-06		
Total	0.00232	263			

Constrained (Type III) sums of squares.

Figure 3a.

Analysis of Variance

Source	Sum Sq.	d.f.	Mean Sq.	F	Prob>F
Concentration	2.19985e-10	2	1.09993e-10	0.23	0.795
Well(Concentration)	1.92842e-08	30	6.42808e-10	1.34	0.1191
Error	1.10662e-07	231	4.79055e-10		
Total	1.30166e-07	263			

Constrained (Type III) sums of squares.

Figure 3b.

Figure 3. ANOVA table for b_1 **(A)** and b_2 **(B)** values.

Table S1. Estimated coefficients for the gain effect model (N = 264)

Coefficients	Median	Interquartile range
b_1	0.24298	0.0035
b_2	$-9.933 \cdot 10 - 4$	$2.5933 \cdot 10^{-5}$

In Figure 3 it can be seen that, assuming a 5% type I risk α, there are no statistically significant differences between coefficients fitted with data from different concentrations (p-values > 0.05 for the concentration factor). As a result, this validates the third assumption of the same gain effect for all concentration levels.

The final b_1 and b_2 values are calculated as the median values of the overall coefficients. The global coefficient values are shown in (Table S1).

3 APPENDIX 3

This appendix compares the output obtained when the bias model from Eq. S5 is used, and when the scaled bias model from Eq. S6 is used instead.

$$Bias = \hat{C} - C_T = d_0 + d_1 \cdot C_T \tag{S5}$$

$$Bias \cdot \frac{1}{C_T} = d_0 \cdot \frac{1}{C_T} + d_1 \tag{S6}$$

As it can be seen in Figure 4a there is a clear heteroscedasticity (i.e., unequal level of dispersion along the range of a variable) in the residuals. This is reflected by bias values being more dispersed for higher values of the concentration. Indeed, this could affect to the quantification of the uncertainty: an increased

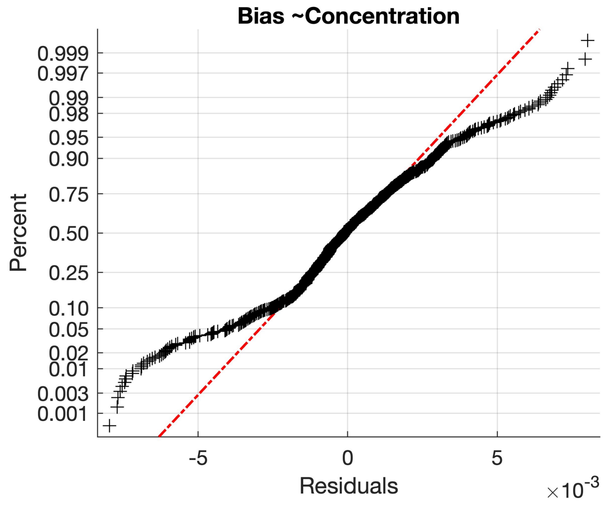

Figure 4a.

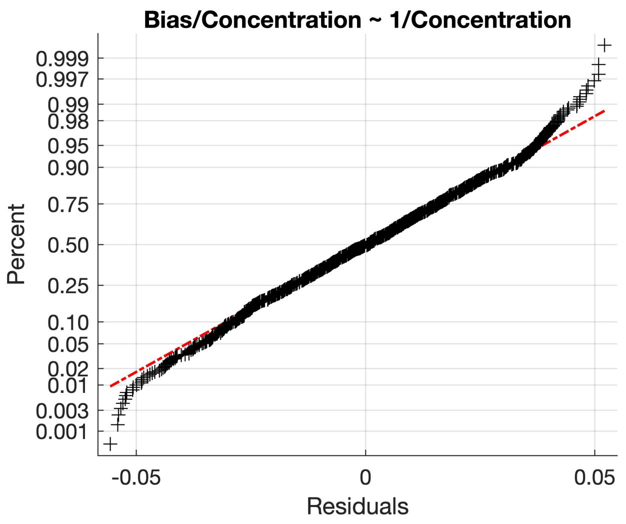

Figure 4b.

Figure 4. Residual analysis with Normal probability plots of the residuals quantifying the uncertainty without including **(A)** and including **(B)** the normalization by the concentration values from Eq. S6

dispersion of the residuals along with concentration values, should be reflected accordingly, yielding higher levels of uncertainty for the predictions of higher concentration values. Nonetheless, as relationship between the error and the magnitude being measured is proportional, heteroscedasticity can be easily neutralized by Equation S6 (Figure 4b).

Figure 4 illustrates the normal probability plots of the residuals after fitting the bias regression model using bias values without (Figure 4a) and with (Figure 4b) the scaling from Eq. S6.

As it can be appreciated, the right normal probability plot, which includes the aforementioned scaling of the residuals by the concentration, fits way better the line of the normal distribution. Thus, the s_{Bias} term will be estimated as the standard deviation of the scaled bias.

4 APPENDIX 4

This appendix contains the sequence of steps obtained when the *in silico* part of the protocol to fit the calibration model, is executed. This document can be found as a pdf and as a livescript in the GitHub repository with the set of functions to execute PLATERO.

PLATE Reader Operator pipeline

Step 1: data loading and preparation

Add PLATERO set of functions to your working directory:

```
my = version('-release');
if str2double(my(1:4))<2020
    addpath(genpath('rprev2020'))
else
    addpath(genpath('r2020'))
end
dirdatasave = pwd; % The directory where the database is stored.
% In this case, the working directory, extracted by the "pwd" command.
```

Step 0: Data read

Load the experimental data. In this case, the data is in the file : "Fluorescein_random_2020_rows_8rep_one_rep_per_sheet.xlsx". The data is organized by sheets. Each sheet has one repetition of the measurements.

```
filename = "Fluorescein_random_2020_rows_8rep_one_rep_per_sheet.xlsx";
colstable = "B102:I197";
gainlevels = 50:10:80;
colnames = {'WellID','Well','Concentration','G50','G60','G70','G80','OD'};

[data_cal, datagfp_val] = prep_data(filename,colstable,gainlevels, ...
    colnames);
```

```
ans = 1×2
        3072           6
This data set has 512 BLK observations and 2560 GFP observations.
The calibration data set has 1760 observations and the validation data set has 800 observations.
```

Step 1: Model fitting

load(strcat(pwd,'calibration.mat'))

```
[blk_data, flu_data] = explore_data(data_cal);
```

```
Explorative plot of missing data for each concentration level:
```

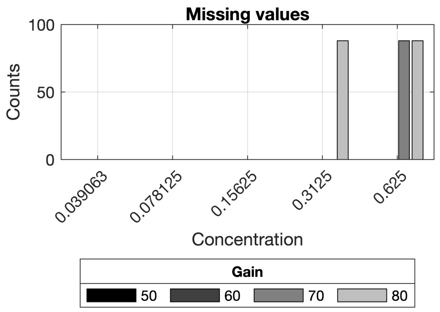

Explorative plot of the raw F_observed Fluorescein data:

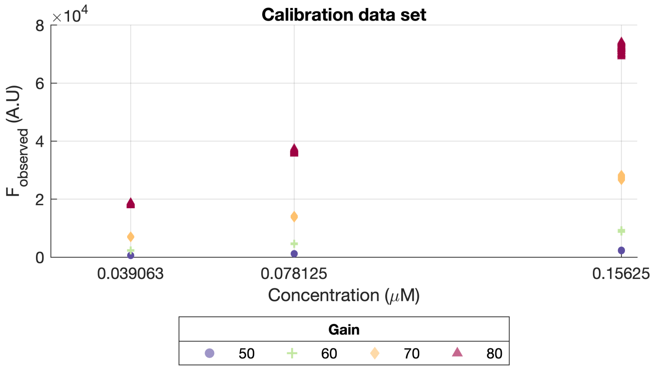

Explorative plot of the raw F_BLK data:

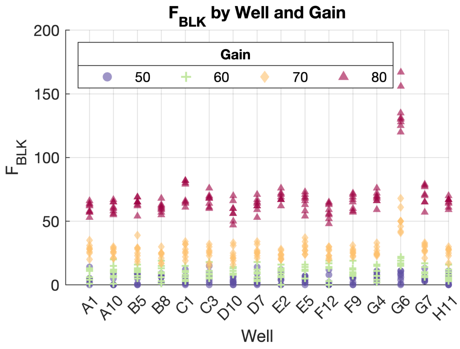

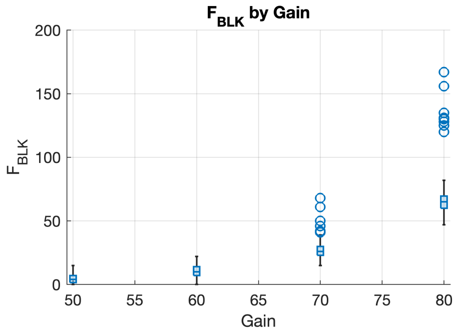


```
% Fit the units conversion equation (eq. X from paper) and return:
% - the predicted concentration as a new column of flu_data;
% - all model coefficients;
% - metrics evaluating the prediction of the concentration for the
% calibration set
[flu_data, model_parameters, calmetrics] = fit_platero_model(blk_data, flu_data);
```

```
Fit f_G and plot corrected data (F_reporter):
ANOVA on coefficient b_1 for all levels of concentration
```

Analysis of Variance

Source	Sum Sq.	d.f.	Mean Sq.	F	Prob>F
Concentration	0	2	1.1491e-06	0.13	0.874
Well(Concentration)	0.00035	30	1.16589e-05	1.37	0.1049
Error	0.00197	231	8.52553e-06		
Total	0.00232	263			

Constrained (Type III) sums of squares.

```
ANOVA on coefficient b_2 for all levels of concentration
```

Analysis of Variance

Source	Sum Sq.	d.f.	Mean Sq.	F	Prob>F
Concentration	2.19985e-10	2	1.09993e-10	0.23	0.795
Well(Concentration)	1.92842e-08	30	6.42808e-10	1.34	0.1191
Error	1.10662e-07	231	4.79055e-10		
Total	1.30166e-07	263			

Constrained (Type III) sums of squares.

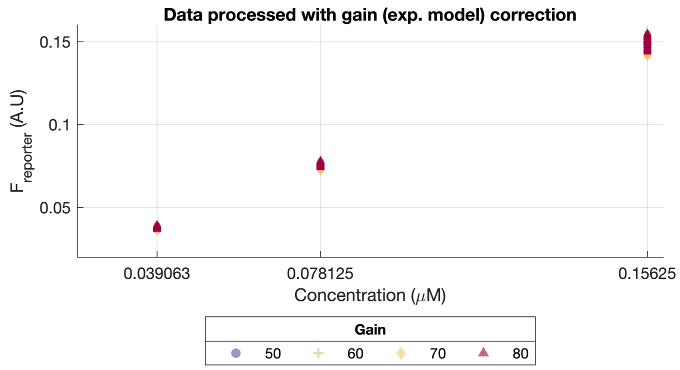


```
Fit f_UC and plot estimated concentration data (C):
```

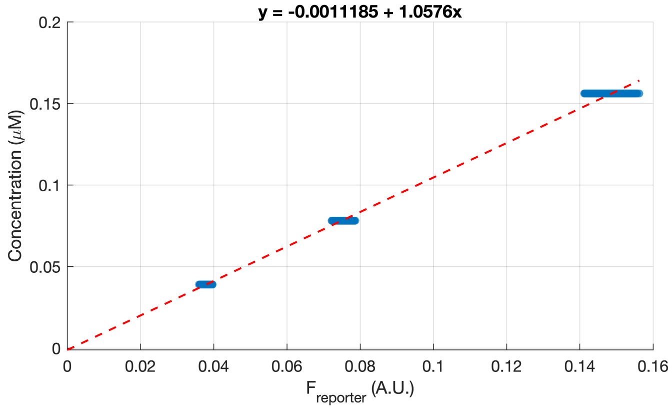


```
Analize the bias in the predictions and estimate the uncertainty in the predictions (s_Bias):
```

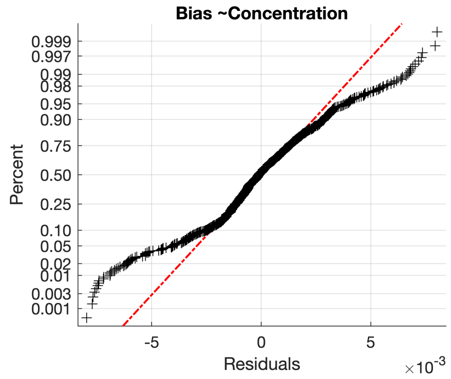

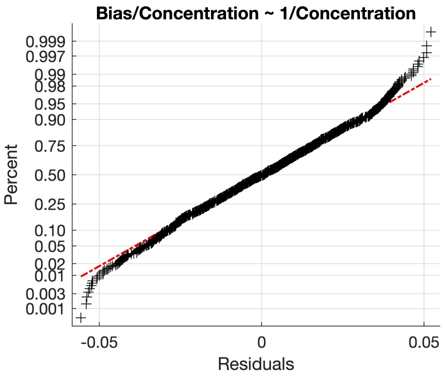

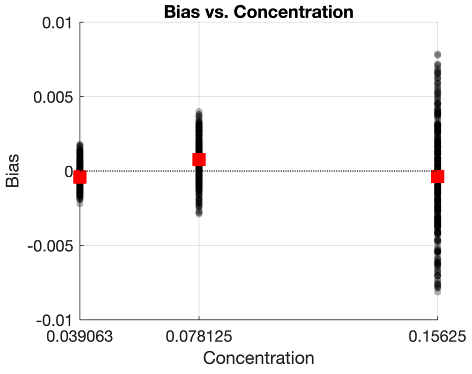

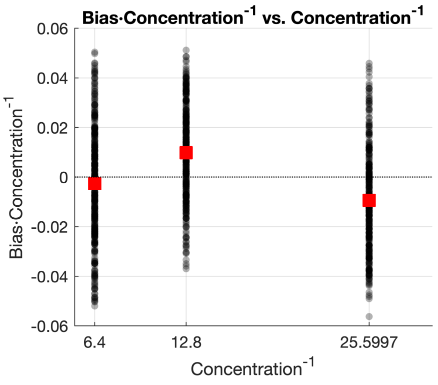

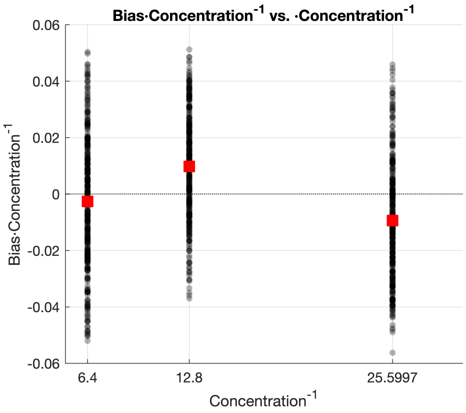

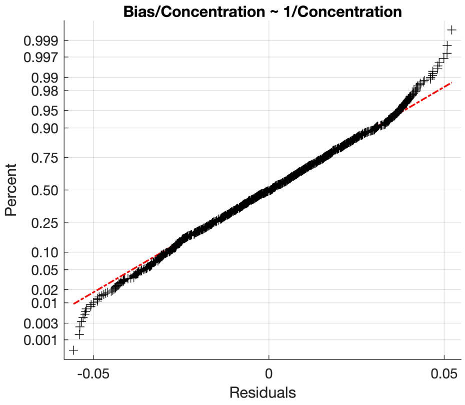


```
Compute error metrics for the Model Building step:
```

F_BLK (G = 50)	F_BLK (G = 60)	F_BLK (G = 70)	F_BLK (G = 80)	b1	b2	[illegible]
4	10	26	65	0.24298	−0.0009933	−0.0[illegible]

Step 3: Model Validation

load(strcat(dirdatasave,'validation.mat')) load(strcat(dirdatasave,'coefficients.mat')) Now, the coefficients obtained in the model fitting step (shown in the previous table), are used to predict the concentration values from the observed fluorescence values that were not used to fit the model.

```
% %%%%%%%%%%%%%%% This code is for the dataset used in the paper %%%%%%%%%%
% %%% The goal is no other than to achieve a table with the following columns:
%
% %%%  F_obs | Gain | F_BLK(G level 1) | ... | F_BLK(G level g)
%
flu_data_val = datagfp_val(datagfp_val.Concentration<0.3,:);
G = unique(flu_data_val.Gain);
% Assign the correspoding F_BLK values to each observation F_obs
flu_data_val.Fblk = repmat(model_parameters{:,["F_BLK (G = 50)", "F_BLK (G = 60)",...
   "F_BLK (G = 70)", "F_BLK (G = 80)"]}', size(flu_data_val,1)/length(G),1);
% %%%%%%%%%%%%%%% End of data preparation step %%%%%%%%%%%%%%%%%%%%%%%%%%%%
%
[flu_data_val, valmetrics_inrange, vprocv] = use_platero_model(flu_data_val, model_para
```

```
Plot the Validation dataset transformed to concentration units:
```

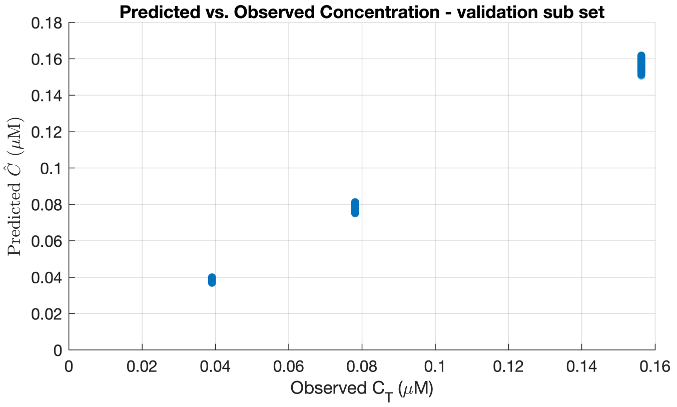


```
R&R Analysis:
R & R Analysis on measurements for C = 0.039063
```

Analysis of Variance

Source	Sum Sq.	d.f.	Mean Sq.	F	Prob>F
Reprod (Gain), C = (0.039063)	2.59442e-05	3	8.64806e-06	124.88	8.06005e-41
Replicates, C = (0.039063)	3.67816e-05	4	9.19539e-06	132.79	1.49776e-48
Error	1.05257e-05	152	6.92483e-08		
Total	7.32515e-05	159			

Constrained (Type III) sums of squares.

```
R & R Analysis on measurements for C = 0.078125
```

Analysis of Variance

Source	Sum Sq.	d.f.	Mean Sq.	F	Prob>F
Reprod (Gain), C = (0.078125)	0.00012	3	4.01839e-05	288.55	1.59411e-62
Replicates, C = (0.078125)	0.0002	4	4.94217e-05	354.89	5.51902e-76
Error	0.00002	152	1.39259e-07		
Total	0.00034	159			

Constrained (Type III) sums of squares.

R & R Analysis on measurements for C = 0.15625

Analysis of Variance

Source	Sum Sq.	d.f.	Mean Sq.	F	Prob>F
Reprod (Gain), C = (0.15625)	0.00041	3	0.00014	266.68	2.51245e-60
Replicates, C = (0.15625)	0.00059	4	0.00015	287.71	8.34823e-70
Error	0.00008	152	0		
Total	0.00107	159			

Constrained (Type III) sums of squares.

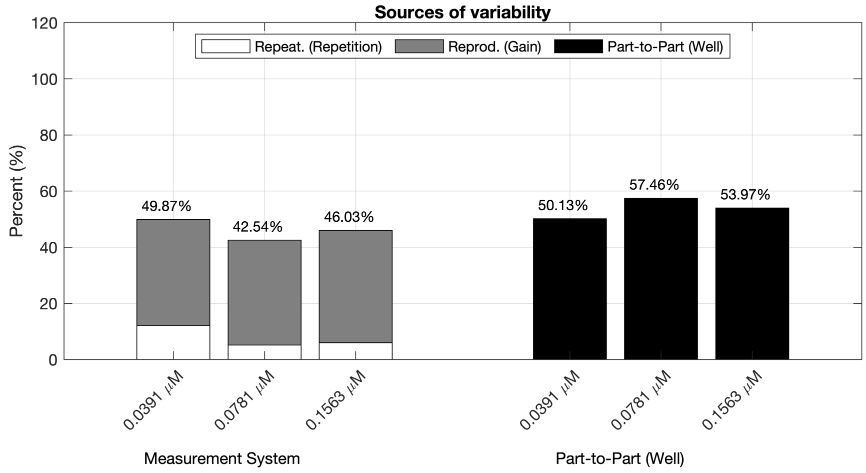

B&L Analysis:

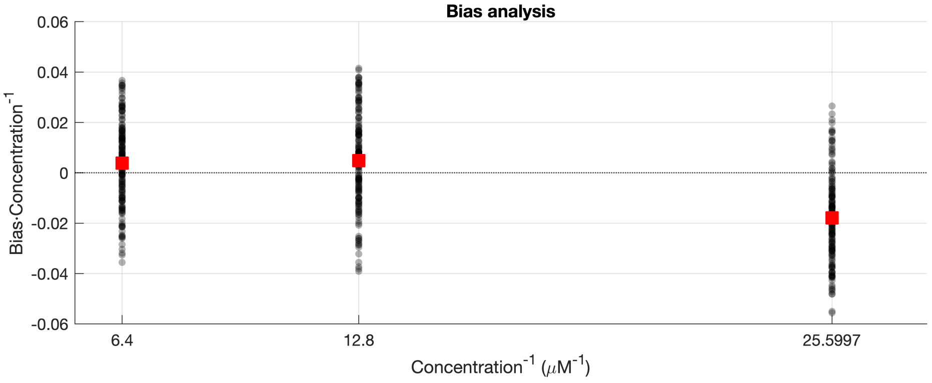

```
Linear regression model:
    y ~ 1 + x1

Estimated Coefficients:
                    Estimate        SE        tStat      pValue
                   __________    __________    _______    __________

    (Intercept)     0.015218     0.0017414     8.7389    4.0025e-17
    x1            -0.0012234    0.00010284    -11.896    9.1553e-29


Number of observations: 480, Error degrees of freedom: 478
Root Mean Squared Error: 0.018
R-squared: 0.228,  Adjusted R-Squared: 0.227
F-statistic vs. constant model: 142, p-value = 9.16e-29
-------------------------------------------------------------------
Contribution of model terms to the total bias variability:
Bias Model - linear term (%): 1.5218 %
Bias Model - bias term (%): 7.5792 %

Confidence Intervals and Error metrics:
        pctgeci: 97.9167
            mse: 3.4285e-06
         relerr: [480×1 double]
    minrelerror: 1.7022e-05
    maxrelerror: 0.0556
```

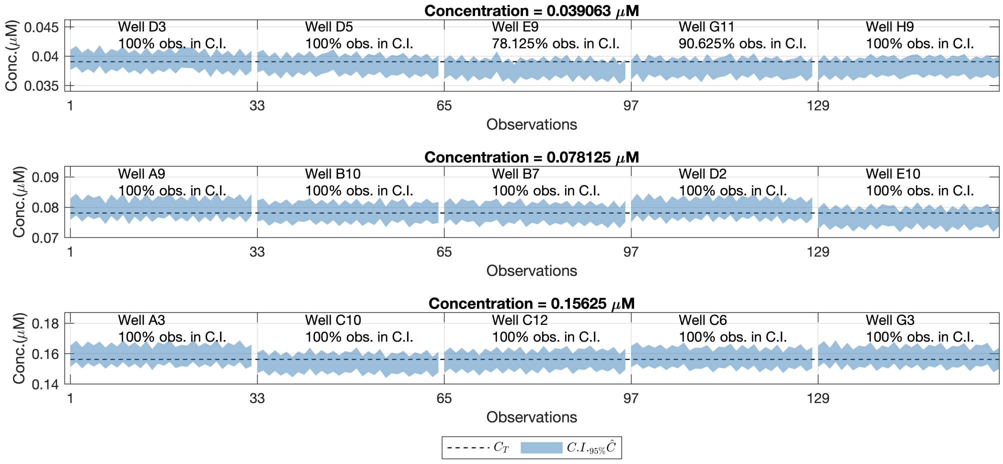


```
% Comparison between calibration-set and validation-set metrics
load(strcat(dirdatasave,'cal_results.mat'))
perftable = table([calmetrics.mse;valmetrics_inrange.mse],...
    [calmetrics.minrelerror;valmetrics_inrange.minrelerror]*100,...
    [calmetrics.maxrelerror;valmetrics_inrange.maxrelerror]*100,...
    'RowNames',{'Calibration', 'Validation (within range)'},...
    'VariableNames',{'MSE','Min.Rel.Error (%)','Max.Rel.Error (%)'});
display(perftable)
```

```
perftable = 2×3 table
```

	MSE	Min.Rel.Error (%)	Max.Rel.Error (%)
1 Calibration	5.6728e-06	0.0040	5.6197
2 Validation (within range)	3.4285e-06	0.0017	5.5619

5 APPENDIX 5

This appendix contains the output rendered when PLATERO is executed with three different measurement systems, as shown in Section 3.4. These documents can be found as a pdf and as a livescript in the GitHub repository with the set of functions to execute PLATERO.

5.1 Plate reader 1

PLATE Reader Operator pipeline

Step 1: data loading and preparation

Add PLATERO set of functions to your working directory:

```
my = version('-release');
if str2double(my(1:4))<2020
    addpath(genpath('rprev2020'))
else
    addpath(genpath('r2020'))
end
```

Now, load the data resulting from the calibration experiment. In this case, we are reading the data from the file "|Fluorescein_random_2020_rows_8rep_one_rep_per_sheet.xls|x". This data is organized by sheets, where each sheet has one repetition of the measurements.

```
filename = "Fluorescein_random_2020_rows_8rep_one_rep_per_sheet.xlsx";
colnames = {'WellID','Well','Concentration','G50','G60','G70','G80','OD'};
[dataPR, indgfp] = readexperiment(filename,"B102:I197",50:10:80,false,colnames);
size(dataPR)
```

```
datPRblk = dataPR(~indgfp,:);
datPRgfp = dataPR(indgfp,:);
disp(strcat("This data set has ", string(size(datPRblk,1)),...
    " BLK observations and ", string(size(datPRgfp,1)), ...
    " GFP observations."))
rng(0207)
[datagfp_cal, datagfp_val] = cvsplit(datPRgfp, 0.7);
disp(strcat("The calibration data set has ", string(size(datagfp_cal,1)),...
    " observations and the validation data set has ", ...
    string(size(datagfp_val,1)), " observations."))
```

```
data_cal_pr1 = [datPRblk; datagfp_cal];
save('calibration_PR2.mat',"data_cal")
save('validation_PR2.mat',"datagfp_val")
```

Test 1:Build model with new data

```
data_cal_pr1 = load("calibration_PR1.mat").data_cal;
[blk_data, flu_data_PR1] = explore_data(data_cal_pr1, nan);
```

```
Explorative plot of missing data for each concentration level:
```

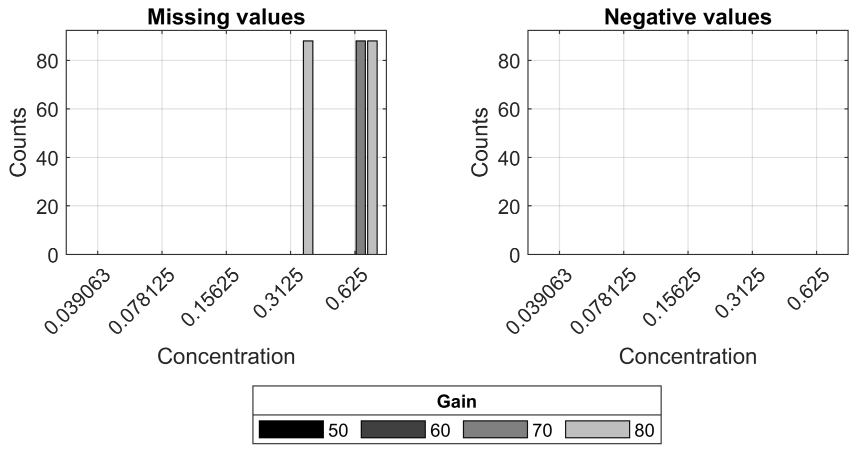


```
Concentration(s)0.3125, 0.625 had missing values
Concentration(s) had negative values
Gain(s)70, 80 had missing values
Gain(s) had negative values
Explorative plot of the raw F_observed Fluorescein data:
```

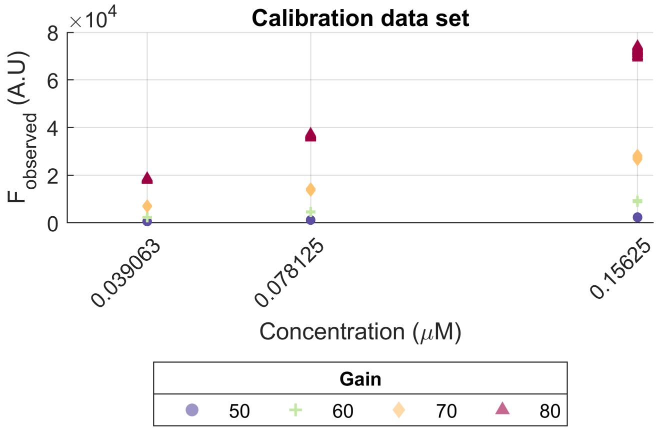


```
Explorative plot of the raw F_BLK data:
```

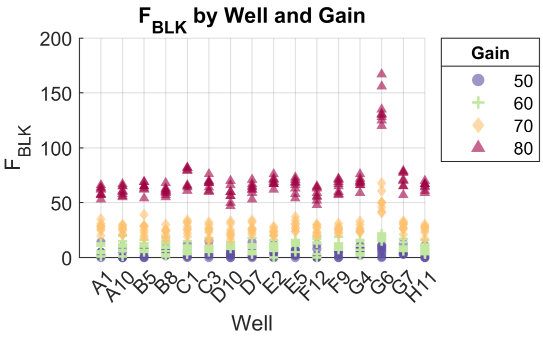

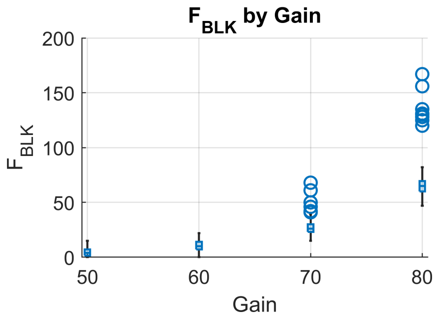


```
[flu_data_PR1, modelPR1, calmetrics_PR1] = fit_platero_model(blk_data, flu_data_PR1);
```

```
Fit f_G and plot corrected data (F_reporter):
ANOVA on coefficient b_1 for all levels of concentration
```

Analysis of Variance

Source	Sum Sq.	d.f.	Mean Sq.	F	Prob>F
Concentration	0	2	1.1491e-06	0.13	0.874
Well(Concentration)	0.00035	30	1.16589e-05	1.37	0.1049
Error	0.00197	231	8.52553e-06		
Total	0.00232	263			

Constrained (Type III) sums of squares.

```
ANOVA on coefficient b_2 for all levels of concentration
```

Analysis of Variance

Source	Sum Sq.	d.f.	Mean Sq.	F	Prob>F
Concentration	2.19985e-10	2	1.09993e-10	0.23	0.795
Well(Concentration)	1.92842e-08	30	6.42808e-10	1.34	0.1191
Error	1.10662e-07	231	4.79055e-10		
Total	1.30166e-07	263			

Constrained (Type III) sums of squares.

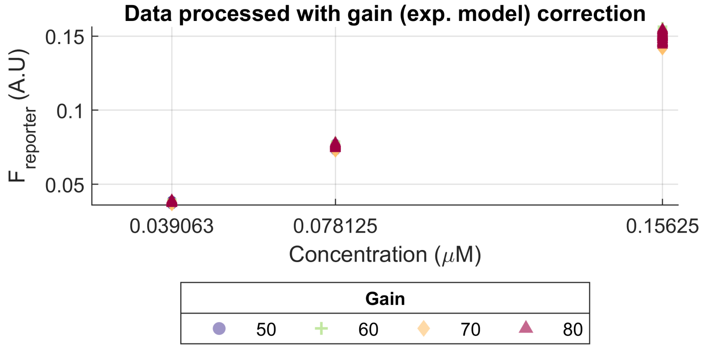

```
Fit f_UC and plot estimated concentration data (C):
```

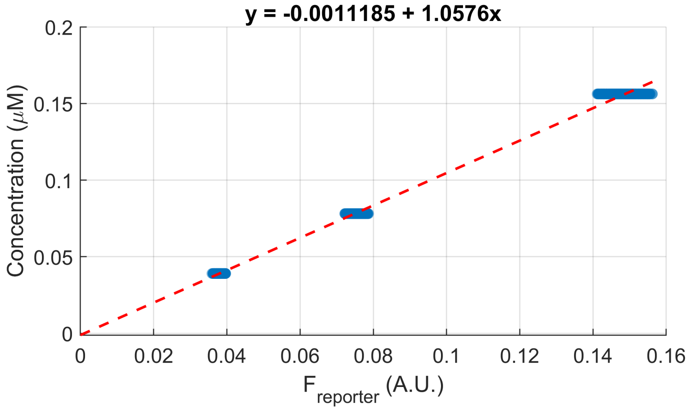


```
Analize the bias in the predictions and estimate the uncertainty in the predictions (s_Bias):
```

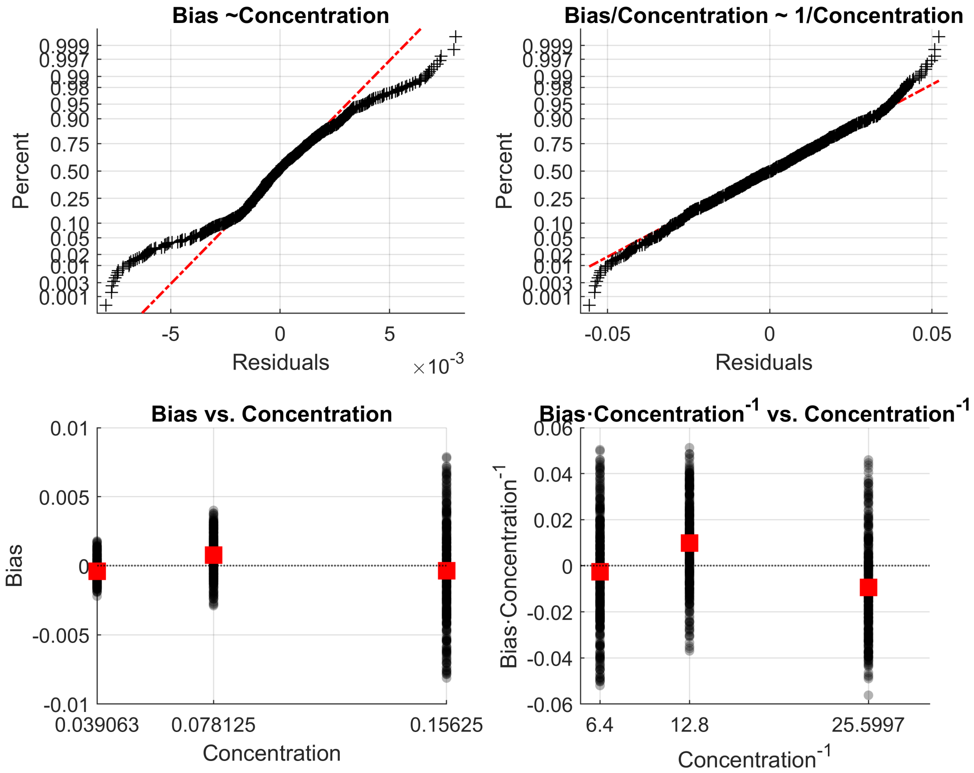

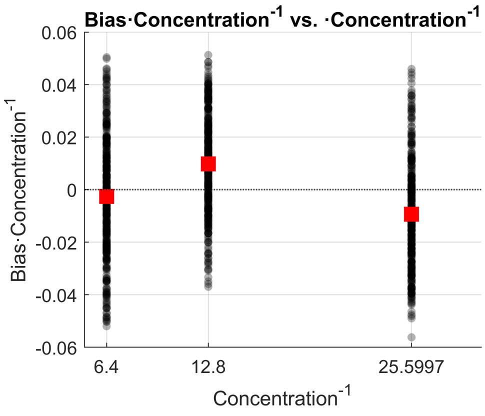

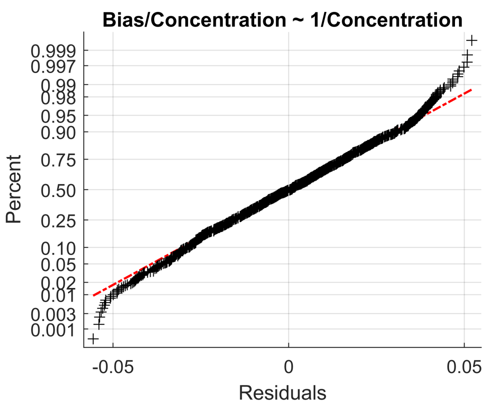


```
Compute error metrics for the Model Building step:
```

F_BLK (G = 50)	F_BLK (G = 60)	F_BLK (G = 70)	F_BLK (G = 80)	b1	b2	c0	[illegible]
4	10	26	65	0.24298	-0.0009933	-0.0011185	[illegible]

Model Validation

Now, the coefficients obtained in the model fitting step (shown in the previous table), are used to predict the concentration values from the observed fluorescence values that were not used to fit the model.

```
% %%%%%%%%%%%%%%% This code is for the dataset used in the paper %%%%%%%%%%
% %%% The goal is no other than to achieve a table with the following columns:
%
% %%%  F_obs | Gain | F_BLK(G level 1) | ... | F_BLK(G level g)
%
datagfp_val = load("validation_PR1.mat").datagfp_val;
uG = unique(flu_data_PR1.Gain);
uC = unique(flu_data_PR1.Concentration);
data_val_pr1 = datagfp_val(ismember(datagfp_val.Gain, uG),:);
%flu_data_val = datagfp_val(datagfp_val.Concentration<0.3,:);
G = unique(data_val_pr1.Gain);
% Assign the correspoding F_BLK values to each observation F_obs
data_val_pr1.Fblk = repmat(modelPR1{:,1:4}', size(data_val_pr1,1)/length(G),1);
% %%%%%%%%%%%%%%% End of data preparation step %%%%%%%%%%%%%%%%%%%%%%%%%%%
%
[data_val_pr1, valmetrics_inrange, vprocv] = use_platero_model(data_val_pr1, modelPR1, "PR1_",
```

```
A 15 % of the observations was missing.
Plot the Validation dataset transformed to concentration units:
```

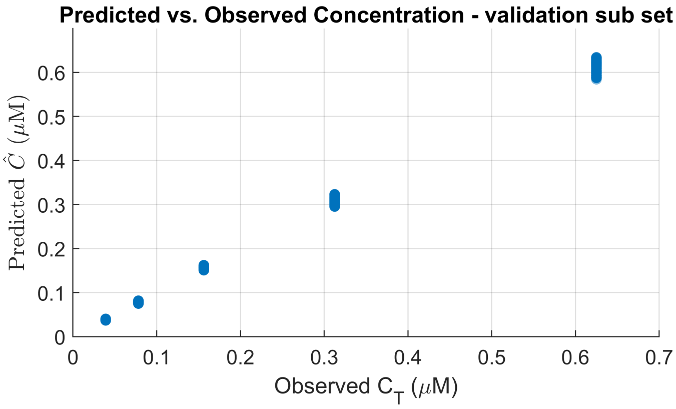

R&R Analysis:
R & R Analysis on measurements for C = 0.039063

Analysis of Variance

Source	Sum Sq.	d.f.	Mean Sq.	F	Prob>F
Reprod (Gain), C = (0.039063)	2.59442e-05	3	8.64806e-06	124.88	8.06005e-41
Replicates, C = (0.039063)	3.67816e-05	4	9.19539e-06	132.79	1.49776e-48

Constrained (Type III) sums of squares.

R & R Analysis on measurements for C = 0.078125

Analysis of Variance

Source	Sum Sq.	d.f.	Mean Sq.	F	Prob>F
Reprod (Gain), C = (0.078125)	0.00012	3	4.01839e-05	288.55	1.59411e-62
Replicates, C = (0.078125)	0.0002	4	4.94217e-05	354.89	5.51902e-76
Error	0.00002	152	1.39259e-07		
Total	0.00034	159			

Constrained (Type III) sums of squares.

R & R Analysis on measurements for C = 0.15625

Analysis of Variance

Source	Sum Sq.	d.f.	Mean Sq.	F	Prob>F
Reprod (Gain), C = (0.15625)	0.00041	3	0.00014	266.68	2.51245e-60
Replicates, C = (0.15625)	0.00059	4	0.00015	287.71	8.34823e-70
Error	0.00008	152	0		
Total	0.00107	159			

Constrained (Type III) sums of squares.

R & R Analysis on measurements for C = 0.3125

Analysis of Variance

Source	Sum Sq.	d.f.	Mean Sq.	F	Prob>F
Reprod (Gain), C = (0.3125)	0.00152	2	0.00076	655.78	6.5467e-63
Replicates, C = (0.3125)	0.0043	4	0.00108	929.06	1.99528e-85
Error	0.00013	113	0		
Total	0.00595	119			

Constrained (Type III) sums of squares.

R & R Analysis on measurements for C = 0.625

Analysis of Variance

Source	Sum Sq.	d.f.	Mean Sq.	F	Prob>F
Reprod (Gain), C = (0.625)	0.00202	1	0.00202	372.82	1.29067e-30
Replicates, C = (0.625)	0.01411	4	0.00353	652.13	7.47094e-57
Error	0.0004	74	0.00001		
Total	0.01653	79			

Constrained (Type III) sums of squares.

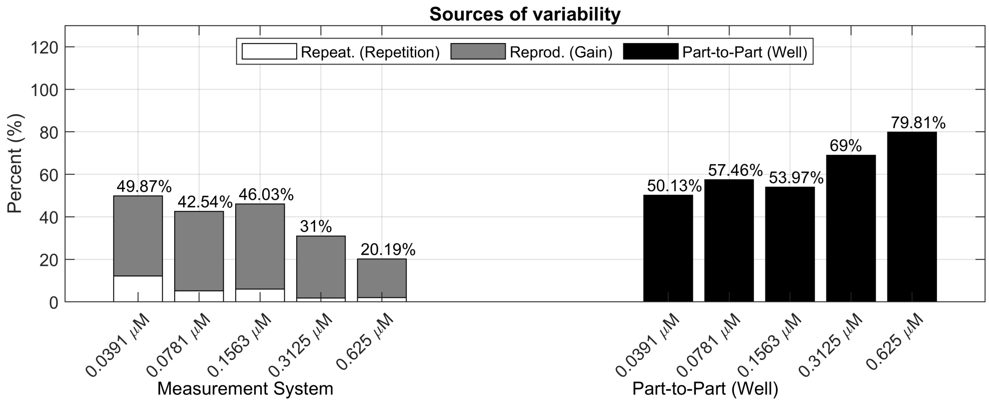

B&L Analysis:

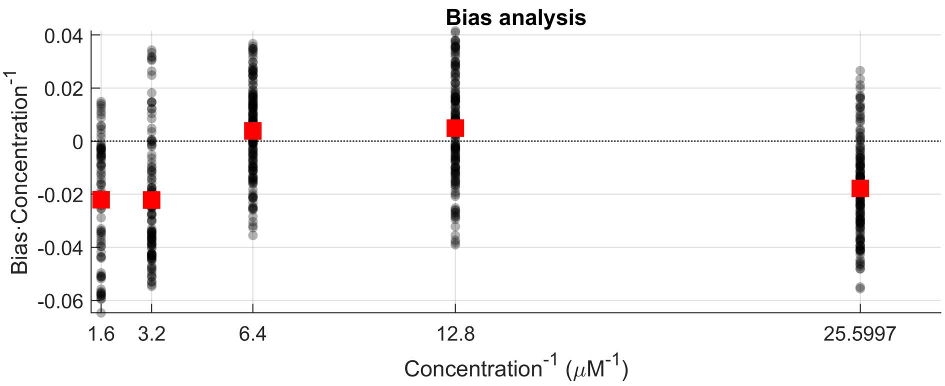


```
Linear regression model:
    y ~ 1 + x1
```

```
Estimated Coefficients:
                   Estimate        SE         tStat       pValue
                  __________    _________    ________    __________

    (Intercept)   -0.0078207    0.0014307     -5.4665    6.4573e-08
    x1            -7.6231e-05   0.00010005   -0.76196       0.44635


Number of observations: 680, Error degrees of freedom: 678
Root Mean Squared Error: 0.0229
R-squared: 0.000856,  Adjusted R-Squared: -0.000618
F-statistic vs. constant model: 0.581, p-value = 0.446
-------------------------------------------------------------------
Contribution of model terms to the total bias variability:
Bias Model - linear term (%): 0.7821 %
Bias Model - bias term (%): 10.1993 %

Confidence Intervals and Error metrics:
        pctgeci: 91.6176
            mse: 6.6503e-05
         relerr: [680×1 double]
    minrelerror: 1.7022e-05
    maxrelerror: 0.0647
```

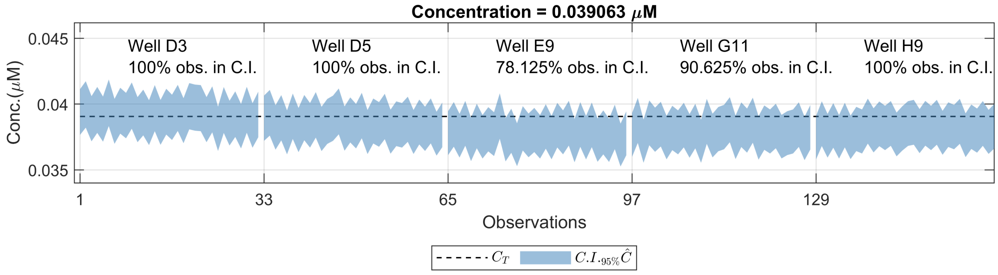

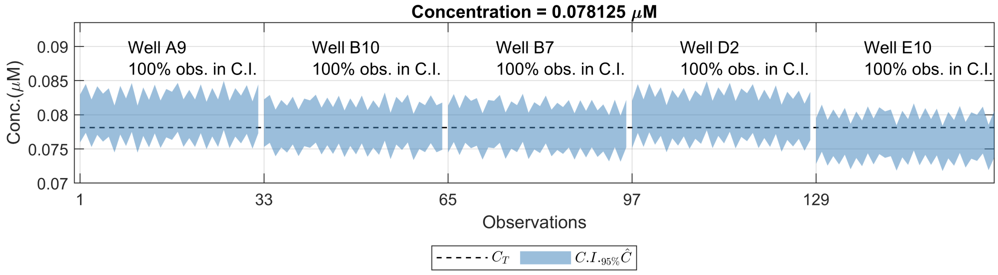

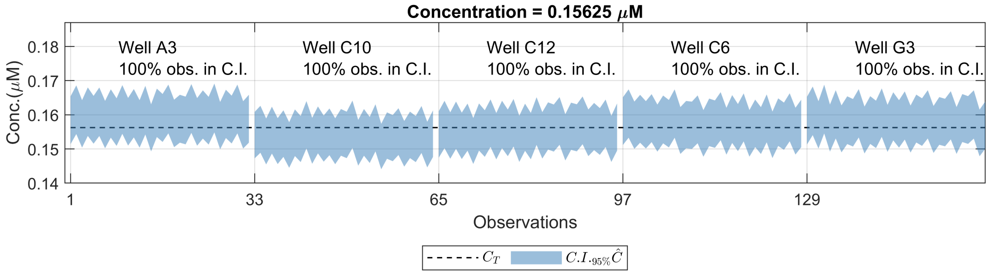

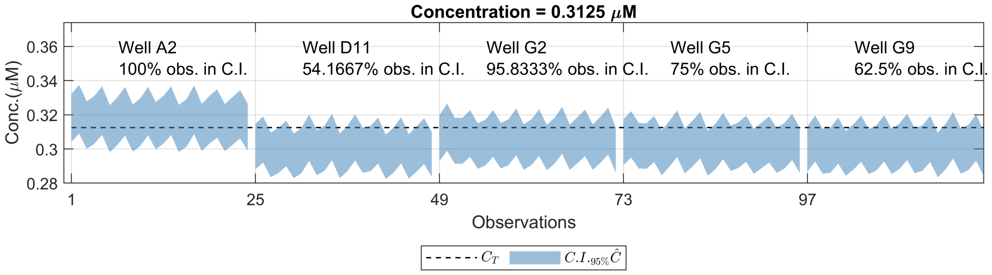

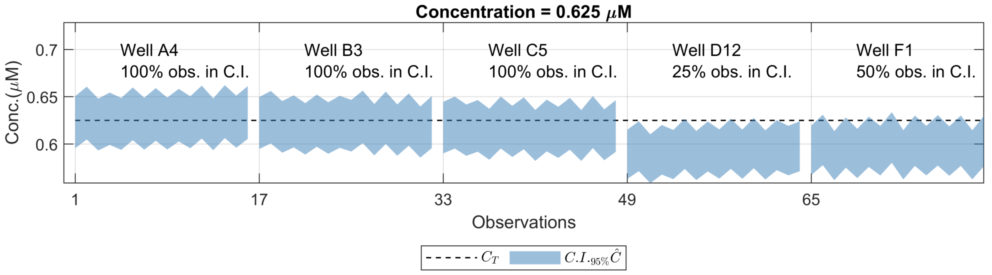


```
% Comparison between calibration-set and validation-set metrics
% load(strcat(dirdatasave,'cal_results.mat'))
perftable = table([calmetrics_PR1.mse;valmetrics_inrange.mse],...
    [calmetrics_PR1.minrelerror;valmetrics_inrange.minrelerror]*100,...
    [calmetrics_PR1.maxrelerror;valmetrics_inrange.maxrelerror]*100,...
    'RowNames',{'Calibration', 'Validation (within range)'},...
    'VariableNames',{'MSE','Min.Rel.Error (%)','Max.Rel.Error (%)'});
display(perftable)
```

perftable = 2×3 table

	MSE	Min.Rel.Error (%)	Max.Rel.Error (%)
1 Calibration	5.6728e-06	0.0040	5.6197
2 Validation (within range)	6.6503e-05	0.0017	6.4732

5.2 Plate reader 2 experiment 1

PLATE Reader Operator pipeline

Step 1: data loading and preparation

Add PLATERO set of functions to your working directory:

```
my = version('-release');
if str2double(my(1:4))<2020
    addpath(genpath('rprev2020'))
else
    addpath(genpath('r2020'))
end
```

Now, load the data resulting from the calibration experiment. In this case, we are reading the data from the file "|Fluorescein_random_2020_rows_8rep_one_rep_per_sheet.xls|x". This data is organized by sheets, where each sheet has one repetition of the measurements.

```
filename = "210922_Tecan_Infinite200_8rep.xlsx";
colnames = {'WellID','Well','Concentration','G50','G60','G70','G80','G90','G120'};
[dataPR, indgfp] = readexperiment(filename,"A7:I103",[50:10:90,120],false,colnames,0);
size(dataPR)
```

```
datPRblk = dataPR(~indgfp,:);
datPRgfp = dataPR(indgfp,:);
disp(strcat("This data set has ", string(size(datPRblk,1)),...
    " BLK observations and ", string(size(datPRgfp,1)), ...
    " GFP observations."))
rng(0207)
[datagfp_cal, datagfp_val] = cvsplit(datPRgfp, 0.7);
disp(strcat("The calibration data set has ", string(size(datagfp_cal,1)),...
    " observations and the validation data set has ", ...
    string(size(datagfp_val,1)), " observations."))
```

```
data_cal_pr2 = [datPRblk; datagfp_cal];
save('calibration_PR2.mat',"data_cal")
save('validation_PR2.mat',"datagfp_val")
```

Test 1:Build model with new data

```
data_cal_pr2 = load("calibration_PR2.mat").data_cal;
[blk_data, flu_data_PR2] = explore_data(data_cal_pr2, 0);
```

```
Explorative plot of missing data for each concentration level:
```

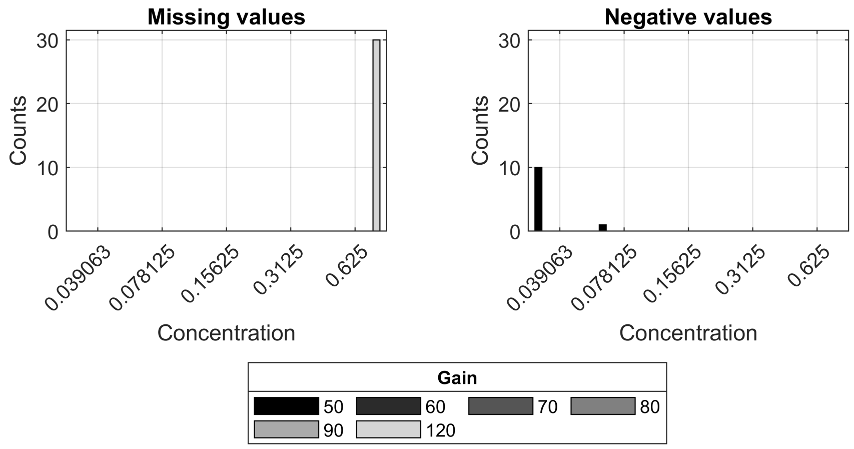


```
Concentration(s)0.625 had missing values
Concentration(s)0.0391, 0.0781 had negative values
Gain(s)120 had missing values
Gain(s)50 had negative values
Explorative plot of the raw F_observed Fluorescein data:
```

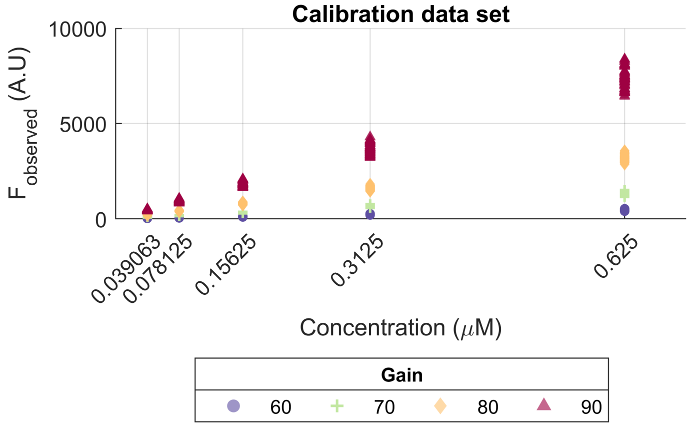


```
Explorative plot of the raw F_BLK data:
```

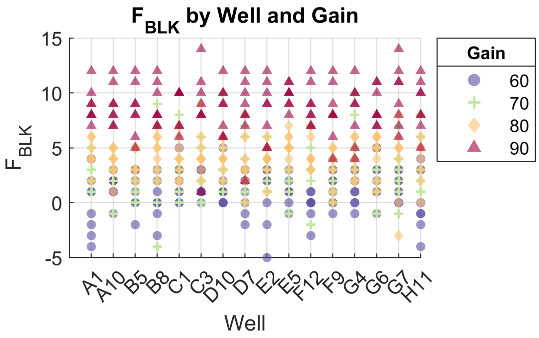

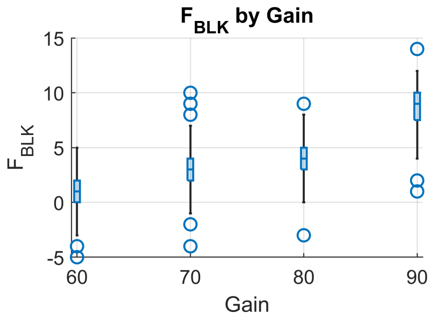


```
[flu_data_PR2, modelPR2_g, calmetrics_PR2] = fit_platero_model(blk_data, flu_data_PR2);
```

```
Fit f_G and plot corrected data (F_reporter):
ANOVA on coefficient b_1 for all levels of concentration
```

Analysis of Variance

Source	Sum Sq.	d.f.	Mean Sq.	F	Prob>F
Concentration	0.00102	4	0.00026	0.33	0.8598
Well(Concentration)	0.01214	50	0.00024	0.31	1
Error	0.30114	385	0.00078		
Total	0.3143	439			

Constrained (Type III) sums of squares.

```
ANOVA on coefficient b_2 for all levels of concentration
```

Analysis of Variance

Source	Sum Sq.	d.f.	Mean Sq.	F	Prob>F
Concentration	4.0355e-08	4	1.00888e-08	0.34	0.8477
Well(Concentration)	4.78955e-07	50	9.57909e-09	0.33	1
Error	1.12713e-05	385	2.92761e-08		
Total	1.17906e-05	439			

Constrained (Type III) sums of squares.

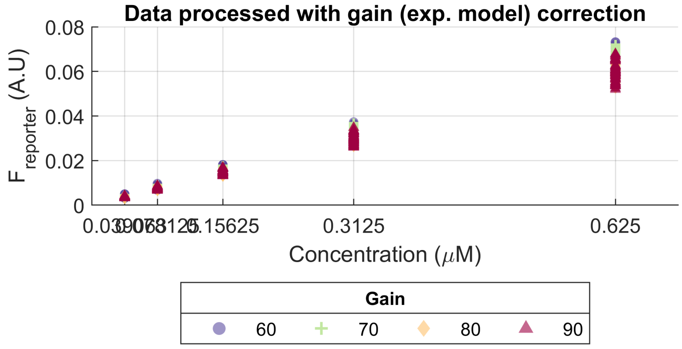

Fit f_UC and plot estimated concentration data (C):

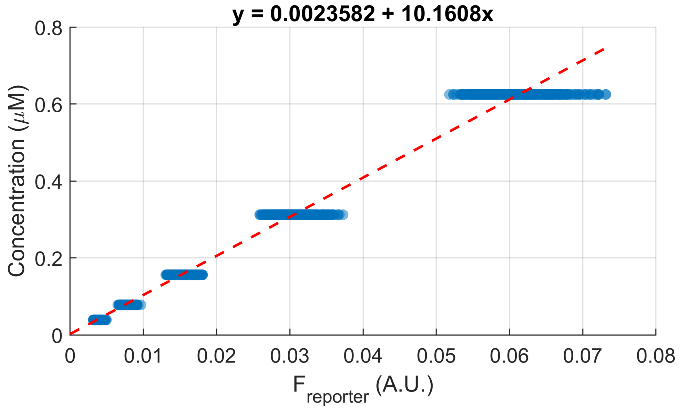

Analize the bias in the predictions and estimate the uncertainty in the predictions (s_Bias):

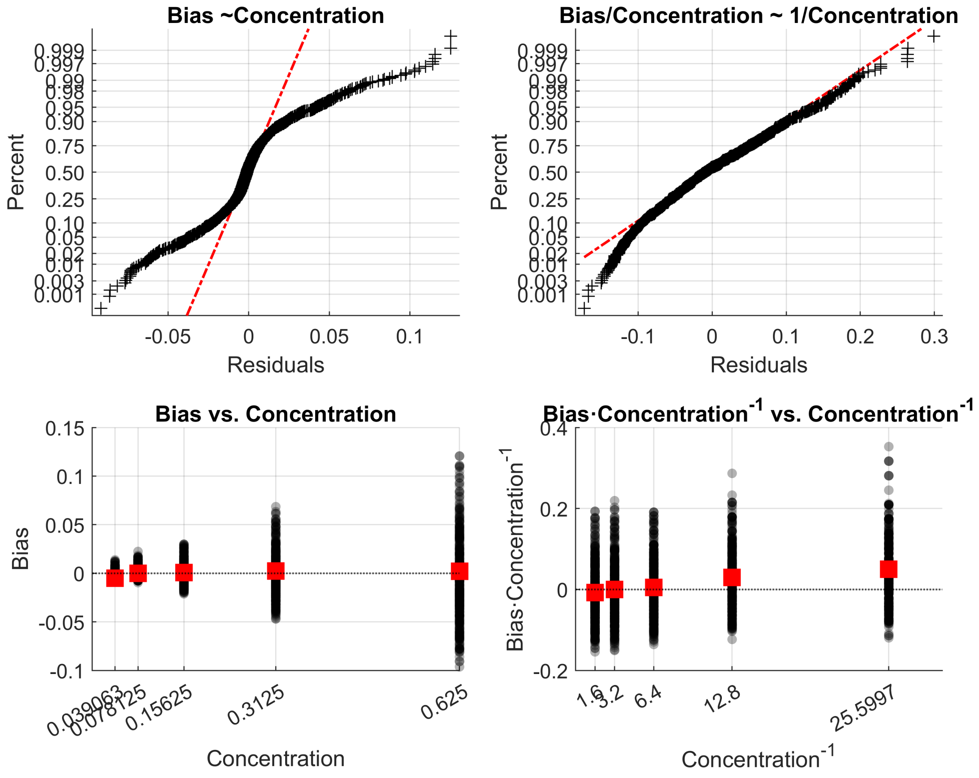

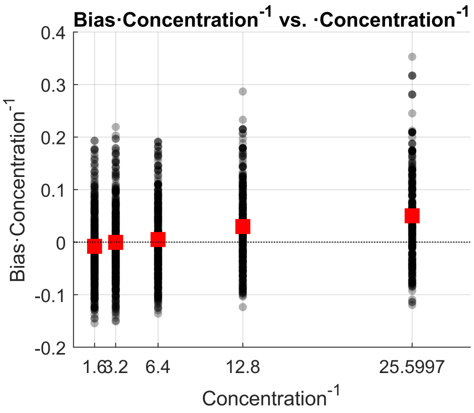

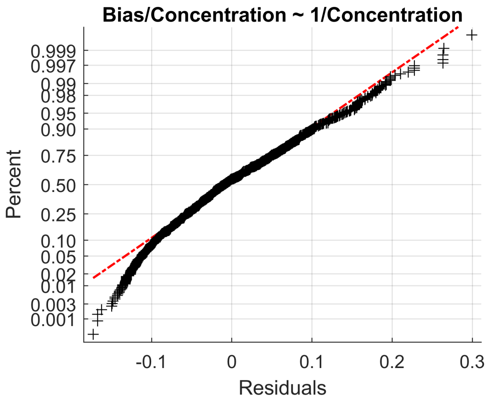


```
Compute error metrics for the Model Building step:
```

F_BLK (G = 60)	F_BLK (G = 70)	F_BLK (G = 80)	F_BLK (G = 90)	b1	b2	c0	[illegible]
1	3	4	9	0.18389	-0.00059598	0.0023582	[illegible]

Model Validation

Now, the coefficients obtained in the model fitting step (shown in the previous table), are used to predict the concentration values from the observed fluorescence values that were not used to fit the model.

```
% %%%%%%%%%%%%%%% This code is for the dataset used in the paper %%%%%%%%%%
% %%% The goal is no other than to achieve a table with the following columns:
%
% %%%  F_obs | Gain | F_BLK(G level 1) | ... | F_BLK(G level g)
%
datagfp_val = load("validation_PR2.mat").datagfp_val;
uG = unique(flu_data_PR2.Gain);
uC = unique(flu_data_PR2.Concentration);
data_val_pr2 = datagfp_val(ismember(datagfp_val.Gain, uG),:);
%flu_data_val = datagfp_val(datagfp_val.Concentration<0.3,:);
G = unique(data_val_pr2.Gain);
% Assign the correspoding F_BLK values to each observation F_obs
data_val_pr2.Fblk = repmat(modelPR2_g{:,1:4}', size(data_val_pr2,1)/length(G),1);
% %%%%%%%%%%%%%%% End of data preparation step %%%%%%%%%%%%%%%%%%%%%%%%%%
%
[data_val_pr2, valmetrics_inrange, vprocv] = use_platero_model(data_val_pr2, modelPR2_g, "PR2_'
```

```
A 0 % of the observations was missing.
Plot the Validation dataset transformed to concentration units:
```

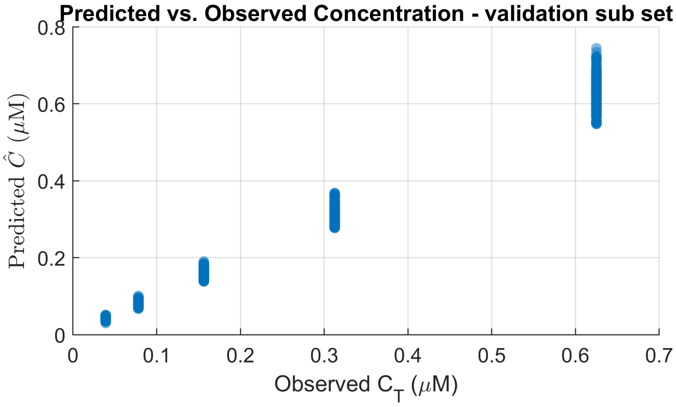


```
R&R Analysis:
R & R Analysis on measurements for C = 0.039063
```

Analysis of Variance

Source	Sum Sq.	d.f.	Mean Sq.	F	Prob>F
Reprod (Gain), C = (0.039063)	0.00007	3	2.2289e-05	2.13	0.0986
Replicates, C = (0.039063)	0.00006	4	1.52638e-05	1.46	0.2172
Error	0.00159	152	1.04565e-05		
Total	0.00172	159			

Constrained (Type III) sums of squares.

```
R & R Analysis on measurements for C = 0.078125
```

Analysis of Variance

Source	Sum Sq.	d.f.	Mean Sq.	F	Prob>F
Reprod (Gain), C = (0.078125)	0.00039	3	0.00013	3.75	0.0124
Replicates, C = (0.078125)	0.00036	4	0.00009	2.6	0.0382
Error	0.00521	152	0.00003		
Total	0.00595	159			

Constrained (Type III) sums of squares.

```
R & R Analysis on measurements for C = 0.15625
```

Analysis of Variance

Source	Sum Sq.	d.f.	Mean Sq.	F	Prob>F
Reprod (Gain), C = (0.15625)	0.00172	3	0.00057	5.08	0.0022
Replicates, C = (0.15625)	0.00042	4	0.0001	0.93	0.4504
Error	0.01719	152	0.00011		
Total	0.01933	159			

Constrained (Type III) sums of squares.

```
R & R Analysis on measurements for C = 0.3125
```

Analysis of Variance

Source	Sum Sq.	d.f.	Mean Sq.	F	Prob>F
Reprod (Gain), C = (0.3125)	0.00713	3	0.00238	5.41	0.0014
Replicates, C = (0.3125)	0.00087	4	0.00022	0.49	0.7395
Error	0.06675	152	0.00044		
Total	0.07476	159			

Constrained (Type III) sums of squares.

R & R Analysis on measurements for C = 0.625

Analysis of Variance

Source	Sum Sq.	d.f.	Mean Sq.	F	Prob>F
Reprod (Gain), C = (0.625)	0.03055	3	0.01018	5.81	0.0009
Replicates, C = (0.625)	0.0134	4	0.00335	1.91	0.1112
Error	0.26629	152	0.00175		
Total	0.31024	159			

Constrained (Type III) sums of squares.

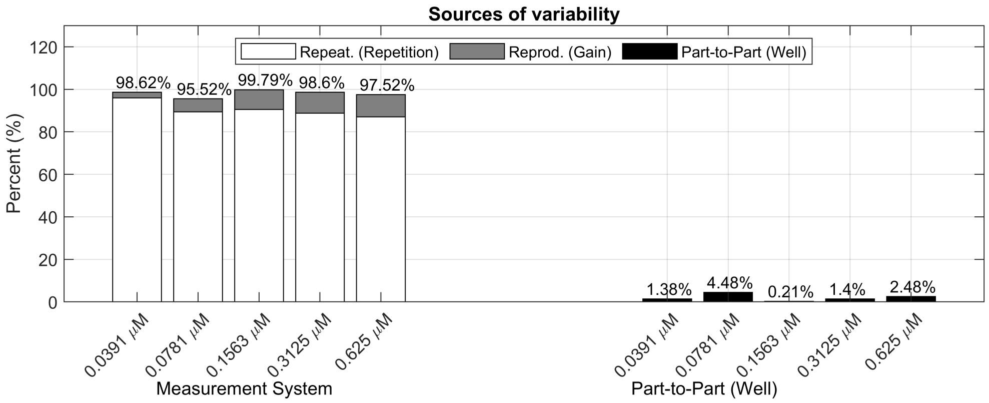

B&L Analysis:

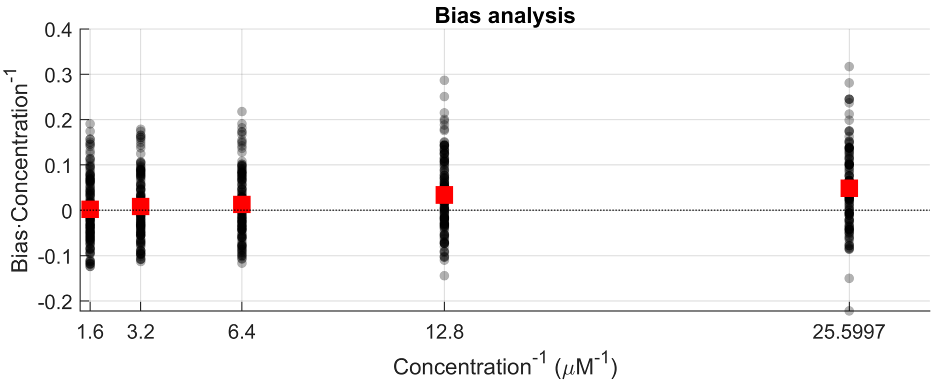


```
Linear regression model:
    y ~ 1 + x1
```

```
Estimated Coefficients:
                   Estimate        SE         tStat       pValue  
                   _________    __________    ______    __________

    (Intercept)    0.0019598     0.0040028    0.4896       0.62455
    x1             0.0019459    0.00030294    6.4233    2.2893e-10


Number of observations: 800, Error degrees of freedom: 798
Root Mean Squared Error: 0.0748
R-squared: 0.0492,  Adjusted R-Squared: 0.048
F-statistic vs. constant model: 41.3, p-value = 2.29e-10
-------------------------------------------------------------------
Contribution of model terms to the total bias variability:
Bias Model - linear term (%): 0.196 %
Bias Model - bias term (%): 4.4521 %

Confidence Intervals and Error metrics:
        pctgeci: 97.5000
            mse: 5.1973e-04
         relerr: [800×1 double]
    minrelerror: 8.3940e-06
    maxrelerror: 0.3169
```

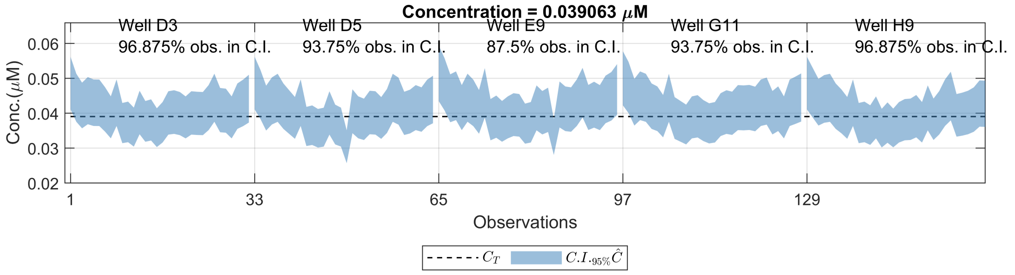

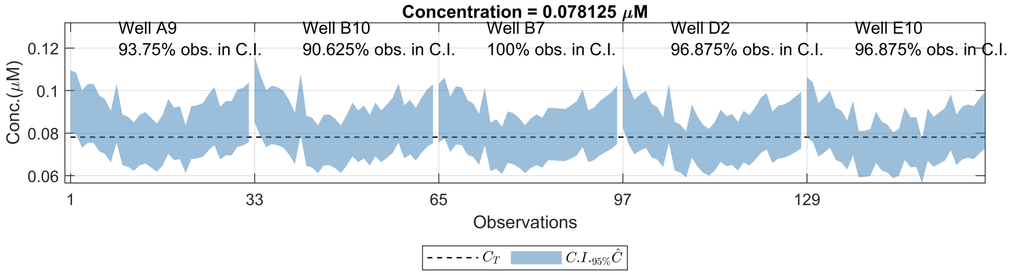

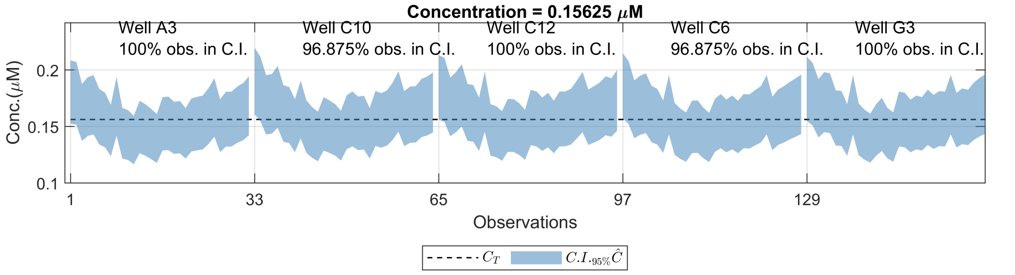

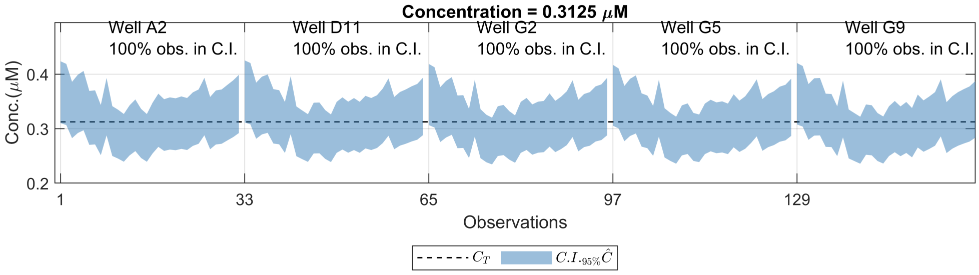

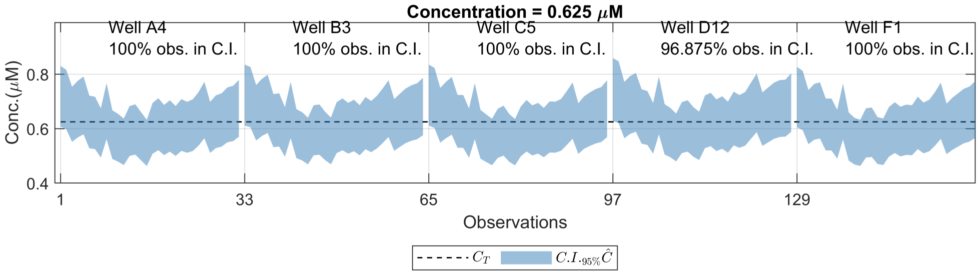


```
% Comparison between calibration-set and validation-set metrics
% load(strcat(dirdatasave,'cal_results.mat'))
perftable = table([calmetrics_PR2.mse;valmetrics_inrange.mse],...
    [calmetrics_PR2.minrelerror;valmetrics_inrange.minrelerror]*100,...
    [calmetrics_PR2.maxrelerror;valmetrics_inrange.maxrelerror]*100,...
    'RowNames',{'Calibration', 'Validation (within range)'},...
    'VariableNames',{'MSE','Min.Rel.Error (%)','Max.Rel.Error (%)'});
display(perftable)
```

perftable = 2×3 table

	MSE	Min.Rel.Error (%)	Max.Rel.Error (%)
1 Calibration	5.5309e-04	0.0045	35.2767
2 Validation (within range)	5.1973e-04	0.0008	31.6867

5.3 Plate reader 2 experiment 2

PLATE Reader Operator pipeline

Step 1: data loading and preparation

Add PLATERO set of functions to your working directory:

```
my = version('-release');
if str2double(my(1:4))<2020
    addpath(genpath('rprev2020'))
else
    addpath(genpath('r2020'))
end
```

Now, load the data resulting from the calibration experiment. In this case, we are reading the data from the file "|Fluorescein_random_2020_rows_8rep_one_rep_per_sheet.xls|x". This data is organized by sheets, where each sheet has one repetition of the measurements.

```
filename = "300922_Tecan_Infinite200_8rep.xlsx";
colnames = {'WellID','Well','Concentration','G50','G60','G70','G80','G90','G120'};
[dataPR, indgfp] = readexperiment(filename,"A7:I103",[50:10:90,120],false,colnames,0);
size(dataPR)
```

```
ans = 1×2
        4608           5
```

```
datPRblk = dataPR(~indgfp,:);
datPRgfp = dataPR(indgfp,:);
disp(strcat("This data set has ", string(size(datPRblk,1)),...
    " BLK observations and ", string(size(datPRgfp,1)), ...
    " GFP observations."))
```

```
This data set has 768 BLK observations and 3840 GFP observations.
```

```
rng(0207)
[datagfp_cal, datagfp_val] = cvsplit(datPRgfp, 0.7);
disp(strcat("The calibration data set has ", string(size(datagfp_cal,1)),...
    " observations and the validation data set has ", ...
    string(size(datagfp_val,1)), " observations."))
```

```
The calibration data set has 2640 observations and the validation data set has 1200 observations.
```

```
data_cal_pr2 = [datPRblk; datagfp_cal];
save('calibration_PR2_30sept_6090.mat',"data_cal")
save('validation_PR2_30sept_6090.mat',"datagfp_val")
```

Test 1:Build model with new data

```
data_cal_pr2 = load("calibration_PR2_30sept_6090.mat").data_cal;
[blk_data, flu_data_PR2] = explore_data(data_cal_pr2, 0);
```

Explorative plot of missing data for each concentration level:

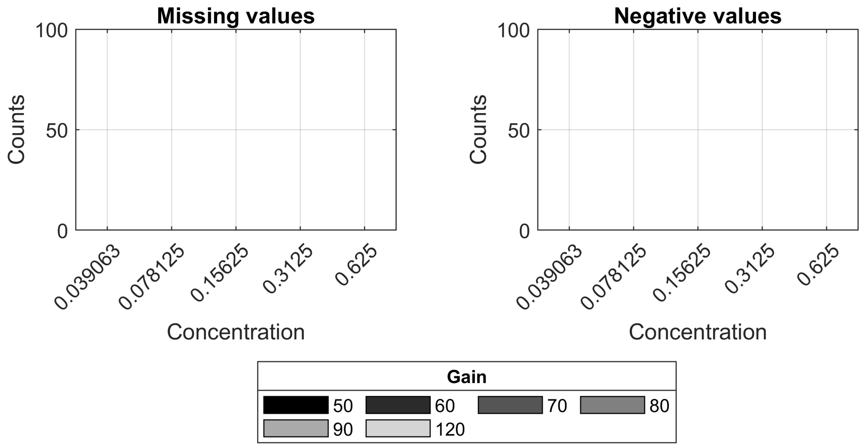

Concentration(s) had missing values
Concentration(s) had negative values
Gain(s) had missing values
Gain(s) had negative values
Explorative plot of the raw F_observed Fluorescein data:

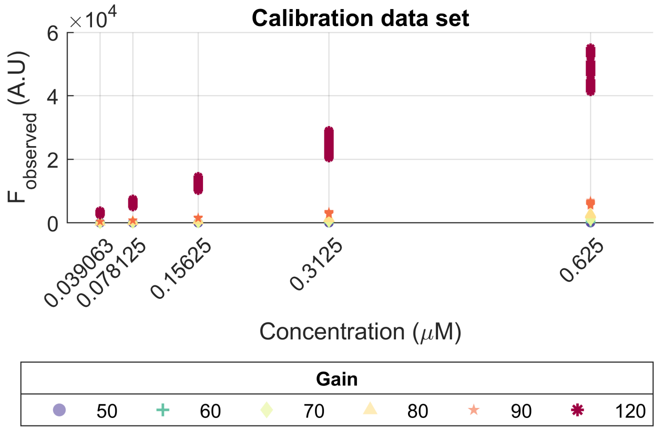

Explorative plot of the raw F_BLK data:

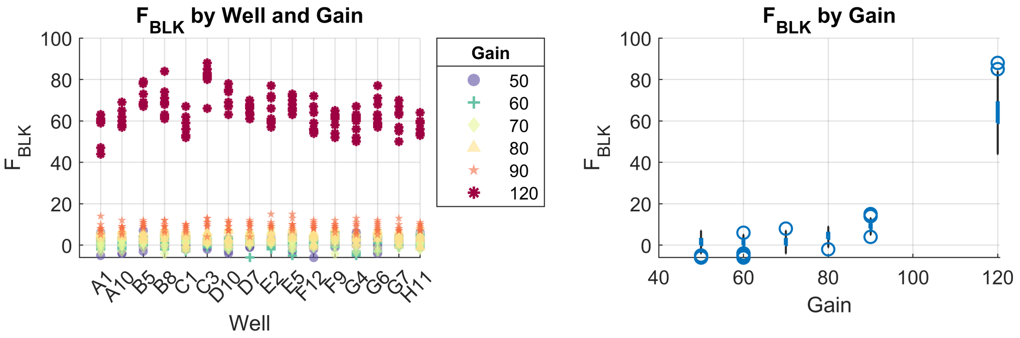


```
% Select gains 60 to 90
blk_data = blk_data(ismember(blk_data.Gain, 60:10:90),:);
flu_data_PR2 = flu_data_PR2(ismember(flu_data_PR2.Gain, 60:10:90),:);
[flu_data_PR2, modelPR2_g6090, calmetrics_PR2] = fit_platero_model(blk_data, flu_data_PR2,"PR2
```

```
Fit f_G and plot corrected data (F_reporter):
ANOVA on coefficient b_1 for all levels of concentration
```

Analysis of Variance

Source	Sum Sq.	d.f.	Mean Sq.	F	Prob>F
Concentration	0.00978	4	0.00244	2.34	0.0542
Well(Concentration)	0.02453	50	0.00049	0.47	0.9992
Error	0.40145	385	0.00104		
Total	0.43575	439			

Constrained (Type III) sums of squares.

```
ANOVA on coefficient b_2 for all levels of concentration
```

Analysis of Variance

Source	Sum Sq.	d.f.	Mean Sq.	F	Prob>F
Concentration	3.77559e-07	4	9.43897e-08	2.3	0.0584
Well(Concentration)	9.65461e-07	50	1.93092e-08	0.47	0.9992
Error	1.5811e-05	385	4.10676e-08		
Total	1.7154e-05	439			

Constrained (Type III) sums of squares.

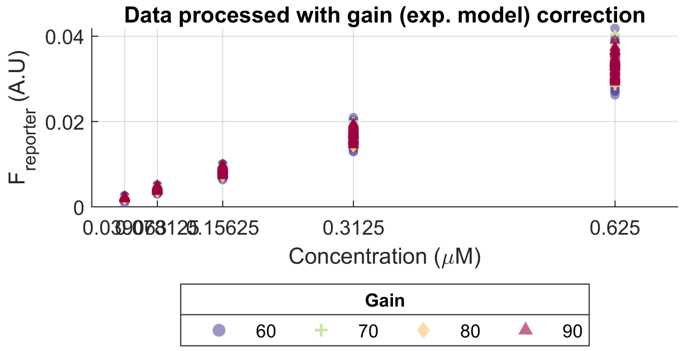


```
Fit f_UC and plot estimated concentration data (C):
```

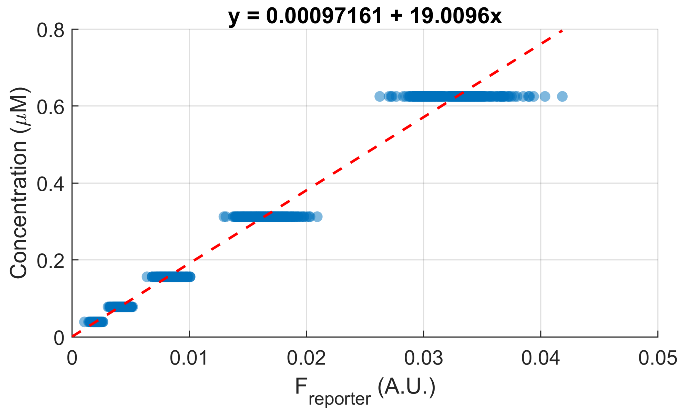


```
Analize the bias in the predictions and estimate the uncertainty in the predictions (s_Bias):
```

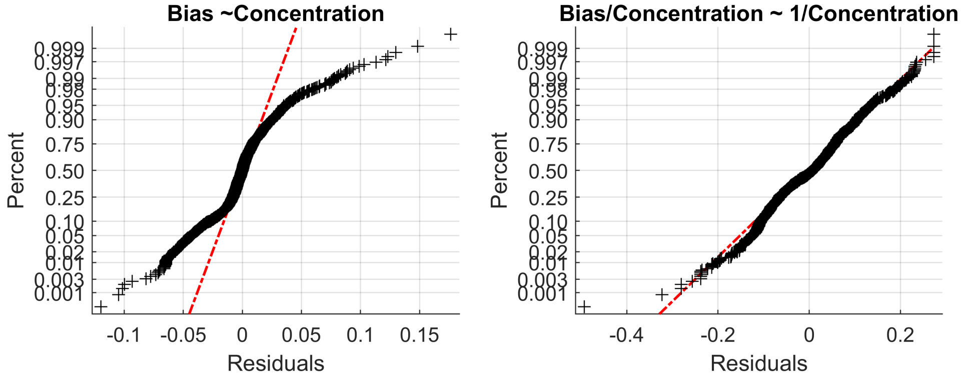

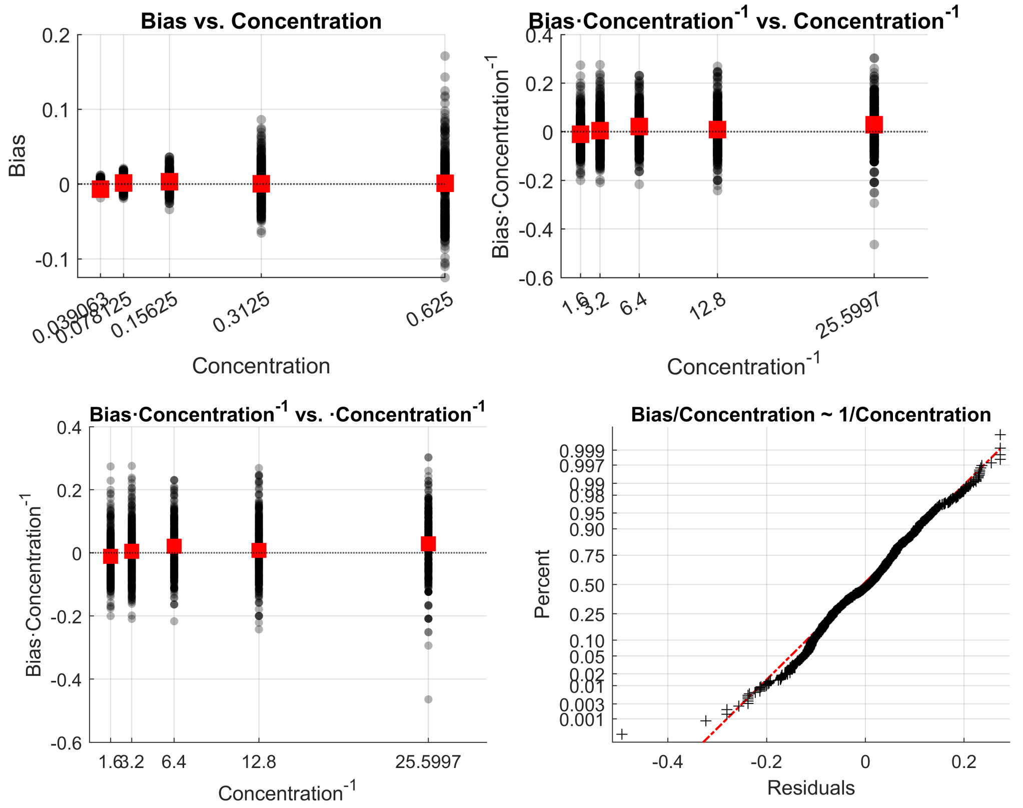


```
Compute error metrics for the Model Building step:
```

F_BLK (G = 60)	F_BLK (G = 70)	F_BLK (G = 80)	F_BLK (G = 90)	b1	b2	c0
1	2	5	9	0.19789	-0.00070272	0.00097161

Model Validation

Now, the coefficients obtained in the model fitting step (shown in the previous table), are used to predict the concentration values from the observed fluorescence values that were not used to fit the model.

```
% %%%%%%%%%%%%%% This code is for the dataset used in the paper %%%%%%%%%%
% %%% The goal is no other than to achieve a table with the following columns:
%
% %%%  F_obs | Gain | F_BLK(G level 1) | ... | F_BLK(G level g)
%
datagfp_val = load("validation_PR2_30sept_6090.mat").datagfp_val;
uG = unique(flu_data_PR2.Gain);
uC = unique(flu_data_PR2.Concentration);
data_val_pr2 = datagfp_val(ismember(datagfp_val.Gain, uG),:);
%flu_data_val = datagfp_val(datagfp_val.Concentration<0.3,:);
G = unique(data_val_pr2.Gain);
% Assign the correspoding F_BLK values to each observation F_obs
```

```
data_val_pr2.Fblk = repmat(modelPR2_g6090{:,1:length(G)}', size(data_val_pr2,1)/length(G),1);
% %%%%%%%%%%%%%%% End of data preparation step %%%%%%%%%%%%%%%%%%%%%%%%%%%
%
[data_val_pr2, valmetrics_inrange, vprocv] = use_platero_model(data_val_pr2, modelPR2_g6090, "
```

```
A 0 % of the observations was missing.
Plot the Validation dataset transformed to concentration units:
```

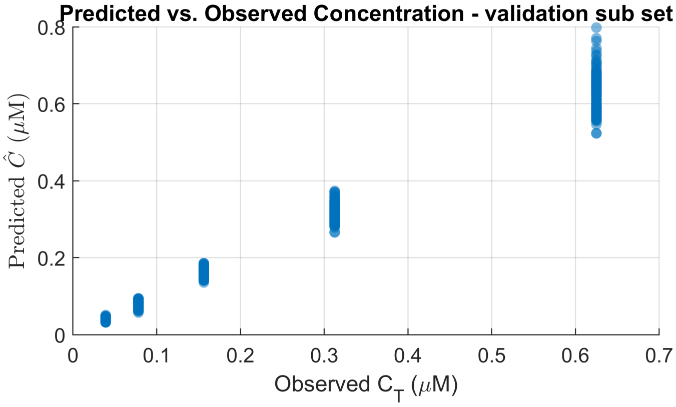


```
R&R Analysis:
R & R Analysis on measurements for C = 0.039063
```

Analysis of Variance

Source	Sum Sq.	d.f.	Mean Sq.	F	Prob>F
Reprod (Gain), C = (0.039063)	0.0001	3	0.00003	5.1	0.0022
Replicates, C = (0.039063)	0.00094	4	0.00023	34.87	0
Error	0.00102	152	0.00001		
Total	0.00206	159			

Constrained (Type III) sums of squares.

```
R & R Analysis on measurements for C = 0.078125
```

Analysis of Variance

Source	Sum Sq.	d.f.	Mean Sq.	F	Prob>F
Reprod (Gain), C = (0.078125)	0.00021	3	0.00007	4.12	0.0077
Replicates, C = (0.078125)	0.00623	4	0.00156	91.08	0
Error	0.0026	152	0.00002		
Total	0.00904	159			

Constrained (Type III) sums of squares.

```
R & R Analysis on measurements for C = 0.15625
```

Analysis of Variance

Source	Sum Sq.	d.f.	Mean Sq.	F	Prob>F
Reprod (Gain), C = (0.15625)	0.00076	3	0.00025	5.9	0.0008
Replicates, C = (0.15625)	0.00898	4	0.00225	52.07	0
Error	0.00656	152	0.00004		
Total	0.0163	159			

Constrained (Type III) sums of squares.

```
R & R Analysis on measurements for C = 0.3125
```

Analysis of Variance

Source	Sum Sq.	d.f.	Mean Sq.	F	Prob>F
Reprod (Gain), C = (0.3125)	0.00255	3	0.00085	4.44	0.005
Replicates, C = (0.3125)	0.04356	4	0.01089	56.88	0
Error	0.0291	152	0.00019		
Total	0.07522	159			

Constrained (Type III) sums of squares.

R & R Analysis on measurements for C = 0.625

Analysis of Variance

Source	Sum Sq.	d.f.	Mean Sq.	F	Prob>F
Reprod (Gain), C = (0.625)	0.01077	3	0.00359	3.94	0.0097
Replicates, C = (0.625)	0.23901	4	0.05975	65.57	0
Error	0.13852	152	0.00091		
Total	0.3883	159			

Constrained (Type III) sums of squares.

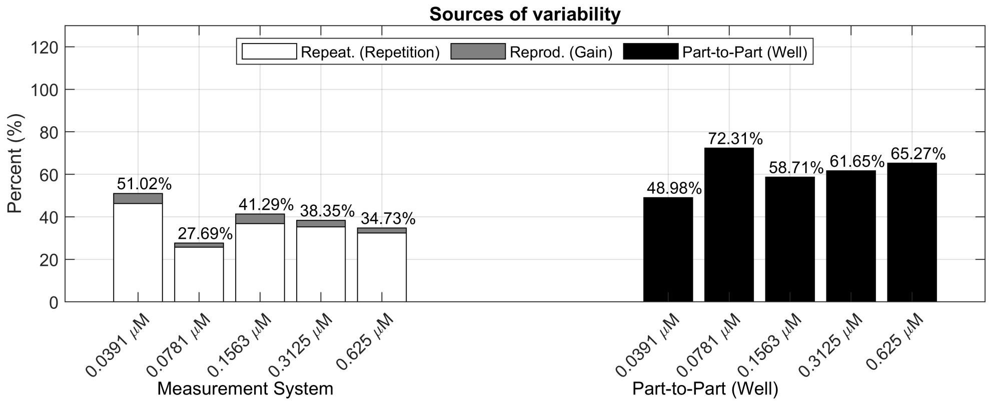

B&L Analysis:

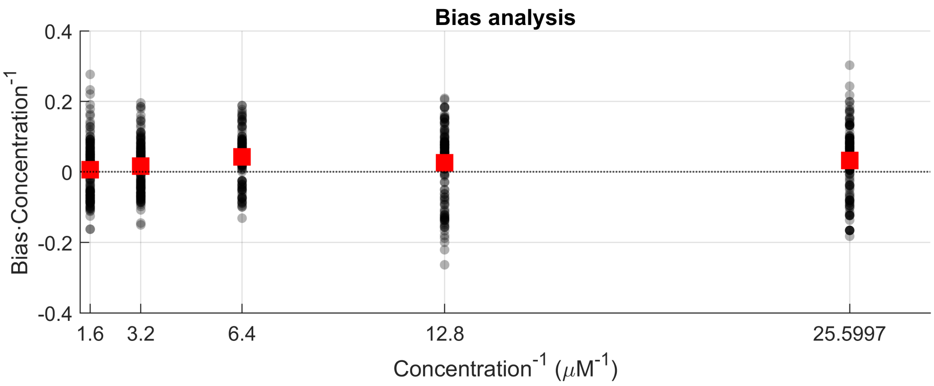


```
Linear regression model:
    y ~ 1 + x1
```

```
Estimated Coefficients:
                   Estimate        SE        tStat      pValue
                  __________    __________    ______    __________

    (Intercept)      0.01764     0.0043869    4.0211    6.3413e-05
    x1            0.00071458    0.00033201    2.1523      0.031675


Number of observations: 800, Error degrees of freedom: 798
Root Mean Squared Error: 0.082
R-squared: 0.00577,  Adjusted R-Squared: 0.00453
F-statistic vs. constant model: 4.63, p-value = 0.0317
-------------------------------------------------------------------
Contribution of model terms to the total bias variability:
Bias Model - linear term (%): 1.764 %
Bias Model - bias term (%): 4.9668 %

Confidence Intervals and Error metrics:
        pctgeci: 96.3750
            mse: 6.3208e-04
         relerr: [800×1 double]
    minrelerror: 6.1634e-04
    maxrelerror: 0.3026
```

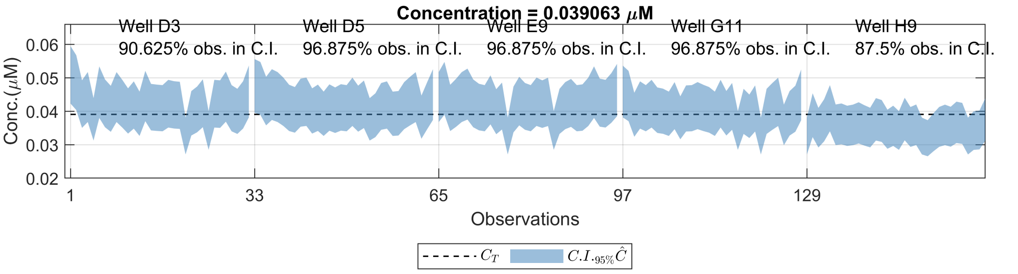

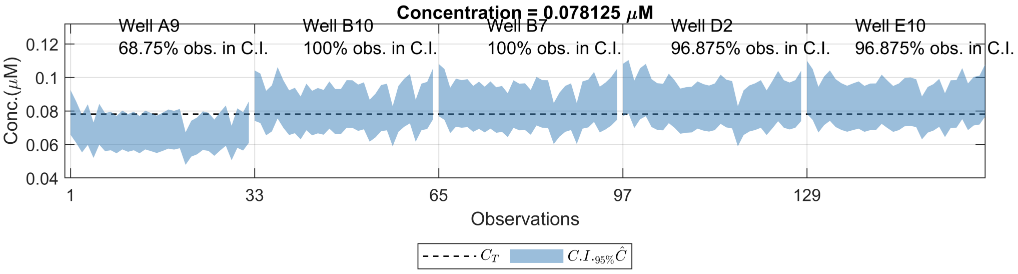

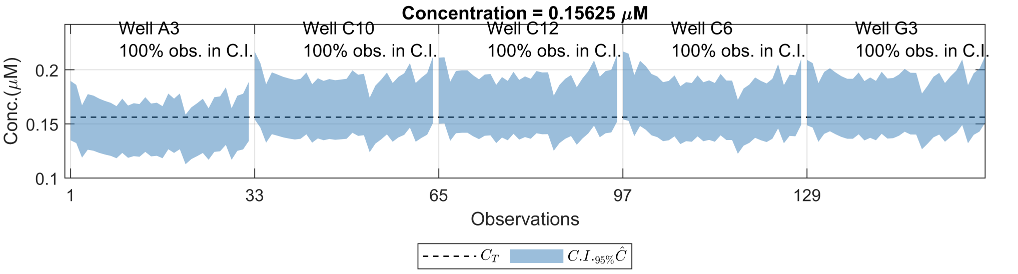

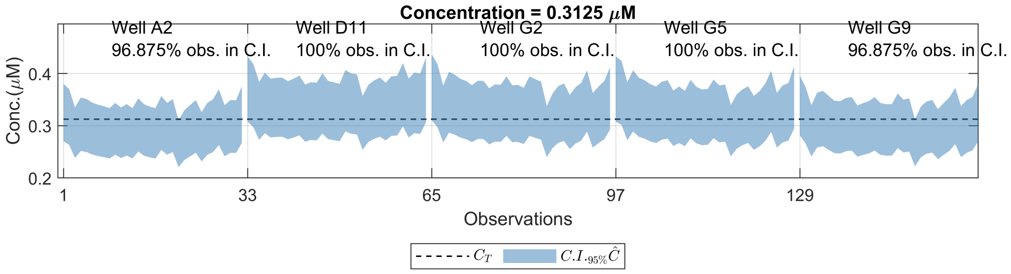

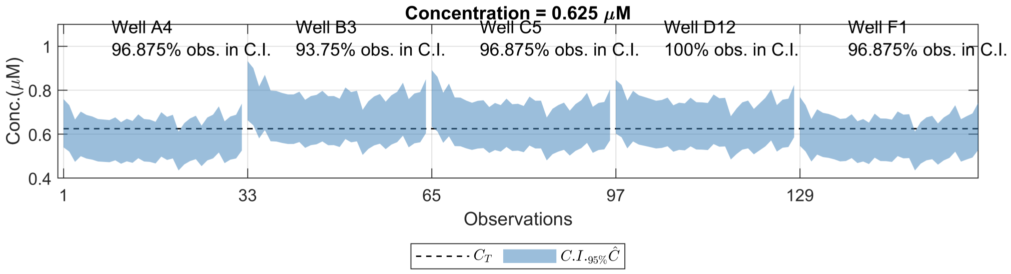


```
% Comparison between calibration-set and validation-set metrics
% load(strcat(dirdatasave,'cal_results.mat'))
perftable = table([calmetrics_PR2.mse;valmetrics_inrange.mse],...
    [calmetrics_PR2.minrelerror;valmetrics_inrange.minrelerror]*100,...
    [calmetrics_PR2.maxrelerror;valmetrics_inrange.maxrelerror]*100,...
    'RowNames',{'Calibration', 'Validation (within range)'},...
    'VariableNames',{'MSE','Min.Rel.Error (%)','Max.Rel.Error (%)'});
display(perftable)
```

perftable = 2×3 table

	MSE	Min.Rel.Error (%)	Max.Rel.Error (%)
1 Calibration	6.0951e-04	0.0004	46.4025
2 Validation (within range)	6.3208e-04	0.0616	30.2628

REFERENCES

George, E., Hunter, W. G., and Hunter, J. S. (2005). *Statistics for experimenters: Design, innovation, and discovery* (Wiley)
